# Unraveling the Multi-Omic Landscape of Extracellular Vesicles in Human Seminal Plasma

**DOI:** 10.3390/biom15060836

**Published:** 2025-06-07

**Authors:** Laura Governini, Alesandro Haxhiu, Enxhi Shaba, Lorenza Vantaggiato, Alessia Mori, Marco Bruttini, Francesca Loria, Natasa Zarovni, Paola Piomboni, Claudia Landi, Alice Luddi

**Affiliations:** 1 Department of Molecular and Developmental Medicine, University of Siena, 53100 Siena, Italy; laura.governini@unisi.it (L.G.); alesandro.haxhiu@student.unisi.it (A.H.); alice.luddi@unisi.it (A.L.); 2 Functional Proteomics Lab, Department of Life Sciences, University of Siena, 53100 Siena, Italy; enxhi.shaba@unisi.it (E.S.); lorenz.vantaggiato2@unisi.it (L.V.); 3 Tuscany Centre for Precision Medicine (CReMeP), Department of Medicine Surgery and Neurosciences, University of Siena, 53100 Siena, Italy; alessia.mori@unisi.it (A.M.); marco.bruttini@unisi.it (M.B.); 4 HansaBioMed Life Sciences Ltd., 12618 Tallinn, Estonia; francesca@hansabiomed.eu (F.L.);; 5 Department of Chemistry and Biotechnology, Tallinn University of Technology, 12618 Tallinn, Estonia

**Keywords:** extracellular vesicles, seminal plasma, male reproductive tract, reproduction, intercellular communication, proteomics, transcriptomics

## Abstract

Extracellular Vesicles (EVs) from seminal plasma have achieved attention due to their potential physiopathological role in male reproductive systems. This study employed a comprehensive proteomic and transcriptomic approach to investigate the composition and molecular signatures of EVs isolated from human seminal plasma. EVs from Normozoospermic (NORMO), OligoAsthenoTeratozoospermic (OAT), and Azoospermic (AZO) subjects were isolated using a modified polymer precipitation-based protocol and characterized for size and morphology. Comprehensive proteomic analysis, using both gel-free and gel-based approaches, revealed distinct protein profiles in each group (*p*<0.01), highlighting potential molecules and pathways involved in sperm function and fertility. The data are available via ProteomeXchange with identifiers PXD051361 and PXD051390, respectively. Transcriptomic analysis confirmed the trend of a general downregulation of AZO and OAT compared to NORMO shedding light on regulatory mechanisms of sperm development. Bioinformatic tools were applied for functional omics analysis; the integration of proteomic and transcriptomic data provided a comprehensive understanding of the cargo content and regulatory networks present in EVs. This study contributes to elucidating the key role of EVs in the paracrine communication regulating spermatogenesis. A full understanding of these pathways not only suggests potential mechanisms regulating male fertility but also offers new insights into the development of diagnostic tools targeting male reproductive disorders.

## 1. Introduction

Seminal fluid contains spermatozoa and secretions from seminal vesicles, accessory glands, and other cellular components of the male genital tract. Proteins, sugars, nucleic acids, growth factors, and lipids are abundant in the seminal plasma. Sperm cells, upon leaving the testis come in contact with this enriched milieu, which regulates their morphology and biochemical composition and modulates their metabolism [1]. Seminal plasma, which assists sperm in maintaining their shape after ejaculation by protecting the integrity of their membranes from oxidative damage, has an impact on post-testes sperm morphology. Decapacitation factors found in seminal plasma stabilize the membrane and prevent premature activation. It also regulates membrane fluidity and provides nutrients, both of which are critical for sperm function and, indirectly, for their shape. Seminal plasma is crucial for preserving the morphology of healthy sperm and ensuring their functional competence until fertilization [2,3,4].

Throughout this journey, spermatozoa also encounter Extracellular Vesicles (EVs), released by numerous cells throughout the male reproductive tract [5]. Based on their size, biogenesis and secretion path, and function, EVs can be roughly divided into three classes: exosomes, 30–150 nm sized vesicles originating from intracellular membranous compartments; microvesicles or ectosomes, sized from 150 to 1000 nm, shed from the cell membrane; and apoptotic bodies with a size varying from 1 to 1.5 µm, released from dying cells [6]. Exosomes and microvesicles can overlap in size and in overall biochemical composition, while they are likely to differ in terms of functionality and specific content mirroring the parent cell’s own function and homeostasis alterations. Exosomes are considered by many as the most relevant class of EVs function wise [7]. These nano-sized membranous vesicles originate from the endosomal system and are shown to transfer their content from cell to cell, inducing phenotypic changes and claiming a relevant role in intracellular communication. They contain a variety of bioactive molecules such as lipids, proteins, DNA, and RNA [8]. Their rich content makes them highly relevant in intercellular signalling and cell–cell communication, enabling them to play pivotal roles in maintaining physiological homeostasis [9]. However, in the context of cancer, these same mechanisms can have detrimental effects, facilitating the spread of pathological conditions [10]. In a male reproductive tract, most abundant populations of EVs are prostasomes and epididymosomes, released, respectively, from prostate and epididymis [11]. The protein content inside these vesicles stands for 3% of the total protein in seminal plasma [12]. Through specific cargo transportation, EVs emerged as important players that assist in sperm maturation, motility acquisition, survival capability throughout female reproductive tract, and, further on, the sperm’s ability to bind to zona pellucida and fertilize the oocyte [11,13,14]. This multifarious EV functionality is accomplished by the transfer of their content to the sperm cell, followed by either downregulating or upregulating a series of proteins expressed throughout different sites of sperm cell structure. The advancement of the transcriptomic and proteomic tools has allowed us to distinguish a heterogeneity regarding the RNA and protein content of the EVs released from different segments of the reproductive tract [15]. Epididymosomes contain RNA coding for numerous proteins responsible for sperm cell maturation, motility, and acquisition of fertilizing ability [16]. Meanwhile, prostasome-born molecules aid sperm cells in regulating the capacitation process, acrosome reactions, and immune suppression in the female reproductive tract [17]. Our study builds up further knowledge of the heterogeneity of EV composition and function in sperm plasma by investigating the differences in the EV-confined expression of several proteins and genes known to be involved in reproduction in subjects with different seminal parameters. The comprehension of the differential expression of EV-shuttled proteins and genes can pin down the molecular pathways involved in fertilization as well as feature a stepping stone for the establishment of EVs as non-invasive biomarkers of male infertility.

## 2. Materials and Methods

### 2.1. Human Biological Sample Collection and Analysis

Semen samples were collected from subjects undergoing semen analysis at the Unit of Medically Assisted Reproduction, Siena University Hospital “Le Scotte”, Italy. All participants were of Caucasian origin and signed a written informed consent to be enrolled in this study. This study’s protocol was approved by the Ethic Committee of the Siena University Hospital (CEAVSE, protocol number 18370, 2 October 2020).

Alcohol consumption, tobacco smoking, drug use, and obesity are identified as risk factors for reduced male fertility. Therefore, to minimize potential confounding factors affecting semen quality, a rigorous selection process for eligible participants was implemented. Subjects were excluded based on the following criteria: smoking; alcohol or drug abuse; presence of varicocele; history of cryptorchidism; systemic or autoimmune diseases; and endocrine disorders, defined as values outside the normal range for key male sex hormones, including follicle-stimulating hormone (FSH), luteinizing hormone (LH), and testosterone (T). The established reference ranges for adult males, based on the diagnostic standards of the Endocrinology Laboratory at Siena University Hospital “Le Scotte”, are as follows: FSH 1.0–9.0 mIU/mL; LH 1.5–7.5 mIU/mL; and T 240–950 ng/dl. Subjects with leukocytospermia (>1 × 10^6^ leukocytes/mL) or semen infection were also excluded from this study.

Semen samples were obtained by masturbation after 3–5 days of sexual abstinence. Semen analysis was performed after 30 min post-liquefaction, according to WHO 2021 guidelines [18]. Two blinded observers conducted the assessments, with the reported data representing the average of their observations. A volume of 10 µL from each semen sample was analyzed as per WHO protocols. An unbiased observer evaluated seminal parameters, and a second observer independently verified the results to ensure quality control. Specifically, 10 µL of semen was placed in a Makler chamber, where a blinded observer assessed sperm concentration and motility by counting total, motile, and non-motile sperm across 10 squares. Sperm vitality was evaluated by mixing the semen with eosin in a 1:1 ratio; after 30 s of incubation, the mixture was smeared onto a slide and examined under a light microscope at 400× magnification. Sperm concentration, motility, morphology, and vitality were assessed for each sample. After semen analysis from each subject, 15 semen samples with normal sperm parameters (Normozoospermia = NORMO; >5th centile of WHO 2021 guidelines [18] reference values), 12 semen samples with altered sperm parameter for concentration, motility and morphology (OligoAsthenoTeratozoospermia = OAT; <5th centile), and 12 semen samples with a total absence of spermatozoa (Azoospermia = AZO), were included in this study (Figure S1).

### 2.2. EV Isolation from Seminal Plasma

EVs from semen samples were isolated following an in-house optimized protocol using a chemical precipitation reagent ev-GAG^®^ (HansaBioMed Life Sciences, Tallinn, Estonia) (Figure S2). According to the proposed protocol [19,20], the samples underwent differential centrifugation steps. Firstly, centrifugation at 1600× *g* 10 min at 4 °C in order to pellet sperm cells, cell debris, and apoptotic bodies. Consecutively, a centrifugation at 16,000× *g* 10 min at 4 °C was followed to pellet microvesicles. Supernatants were stored at −80 °C until use. The supernatant was passed through a 0.22 µm filter. After that, 200 µL of ev-GAG^®^ was added to 400 µL of seminal plasma, in an optimized dilution ratio (reagent/sample 1:2). Samples were incubated for 5 min at 4 °C, followed by the centrifugation of 3500× *g* for 30 min at 4 °C. The pellet was washed three times with PBS. Each semen sample was processed in triplicate, to obtain three pellets: one was resuspended immediately in Lysis Buffer RTL (Qiagen, Hilden, Germany) for RNA extraction, and the others stored at −80 °C for proteomic analysis and for EV identification and characterization procedures.

### 2.3. EV Characterization

#### 2.3.1. Nanoparticle Tracking Analysis

The characterization of EVs and EV-depleted controls by Nanoparticle Tracking Analysis (NTA) was performed using ZetaView PMX-120 (Particle Metrix, Inning am Ammersee, Germany), as previously described [21].

#### 2.3.2. Cholesterol Quantification Assay

The total cholesterol content of EVs and EV-depleted controls was quantified by Cholesterol Quantification Assay Kit (HansaBioMed Life Sciences, Tallinn, Estonia), according to the manufacturers’ protocol. Fluorescence intensities were measured (λex = 540 nm; λem = 590 nm) using Tecan GENios Pro microplate reader (Männedorf, Switzerland).

#### 2.3.3. Enzyme-Linked Immunosorbent Assay

Tetraspanin (CD63 and CD9) expression of EVs and EV-depleted controls was analyzed by sandwich enzyme-linked immunosorbent ELISA assay, using ExoTEST^TM^ Ready to Use Kit (HansaBioMed Life Sciences Ltd.), according to the manufacturers’ protocol. Optical densities were measured at 450 nm using Tecan GENios Pro microplate reader. The results are reported as signal-to-noise ratios (SNRs).

#### 2.3.4. Transmission Electron Microscopy Analysis

Samples were prepared for transmission electron microscopy by the conventional negative staining procedure [22]. In brief, 3 μL aliquots of EVs suspension were sedimented for 2 min onto a 300 mesh, copper/carbon-coated grid and then negatively stained with 1% uranyl acetate and observed with a TEM Fei Tecnai G2 spirit at 80 Kv. The obtained digital images were processed with Image J software (version 1.53) acquisition.

### 2.4. Proteomic Analysis—Gel-Free Analysis

#### 2.4.1. EVs Preparation for Liquid Chromatography–Tandem Mass Spectrometry

EV pellets obtained by EV-GAG precipitation from NORMO seminal plasma were suspended in 10 µL of denaturing solution composed by 8 M Urea, 2 M Thiourea, 4% *w*/*v* 3-[(3-cholamidopropyl) dimethylammonia]-1-propanesulfonate hydrate (CHAPS) and 1% *w*/*v* dithioerythritol (DTE) and 10 µL of Laemmli buffer 2x: 100 mM Tris–HCl pH 6.8, 2% (*w*/*v*) SDS, 20% (*v*/*v*) glycerol, 4% (*v*/*v*) β-mercaptoethanol, and traces of bromophenol blue. The sample was heated at 95 °C for 7 min, and total proteins were loaded and resolved on a 10% polyacrylamide gel. Monodimensional gel was stained according to MS-compatible silver staining protocol [23]. Monodimensional resolved protein lane was cut into three parts. All three sections were cut into 1 mm^3^ gel pieces. Each part was destained and dehydrated, as reported for the MALDI-ToF/ToF protocol. Gel pieces were rehydrated, and proteins were reduced by 100 mM DTE in 25 mM AB for 1 h at room temperature and alkylated in 100 mM iodoacetamide in 25 mM AB for 1 h at room temperature in the dark. Following 25 mM AB for 10 min, gel pieces were dehydrated by 25 mM AB in 50% ACN and twice for 100% ACN for 10 min.

Then, proteins were digested by the trypsin solution overnight at 37 °C. The day after, the supernatant was recovered, and peptide extraction was performed in 3 steps. All supernatants were recovered and joined together. First step: gel pieces were covered by 50% ACN and 5% TFA under shaking for 30 min. The second step was performed with 50% ACN and 0.5% TFA, 30 min under shaking. The third step was performed in pure ACN after shaking for 20 min. After peptide extraction, final peptides were purified by ZipTips Millipore^®^ Micro-C18 (Merck KGaA, Darmstadt, Germania), following the manufacturer’s instructions. Eluted peptides were dried in speedvac.

#### 2.4.2. LC-MS/MS Analysis

Digested samples were reconstituted in 0.1% formic acid in water. LC-MS/MS analyses were performed using Q-Exactive HF-X Orbitrap mass spectrometer (Thermo Fisher Scientific, Waltham, MA, USA). Peptide separation was carried out at 35 °C using a PepMap RSLC C18 column, 75 μm × 15 cm, 2 μm, 100 Å (Thermo Fisher Scientific, Waltham, MA, USA) at a flow rate of 300 nl/min. The mobile phases A and B used for the analysis were 0.1% formic acid in water and 0.1% formic acid in acetonitrile, respectively. The gradient started with 5% of B and then it was increased up to 90% in 120 min. The experiment was performed using a data-dependent analysis (DDA) setting to select the “top twenty” most-abundant ions for MS/MS analysis. Protein identification was performed using Proteome Discover 2.5 (Thermo Fisher Scientific, Waltham, Massachusetts, USA). The mass spectrometry proteomics data were deposited to the ProteomeXchange Consortium [24] via the PRIDE [25] partner repository with the dataset identifier PXD051361 (reviewer_pxd051361@ebi.ac.uk).

### 2.5. Proteomic Analysis—Gel-Based Analysis

#### 2.5.1. EV Preparation for Gel-Based Proteomic Analysis

EV pellets prepared from NORMO, OAT, and AZO seminal plasma were solubilized in 100 µL of denaturing solution. After protein denaturation, samples were precipitated in cold acetone (1:4) overnight at −20 °C. The day after, samples were centrifuged at 20,000× *g* per 20 min. The obtained pellets were washed twice in cold acetone. After centrifugation at 20,000× *g* per 20 min, supernatants were discarded, and pellets were newly suspended in 100 µL of denaturing solution and traces of bromophenol blue.

#### 2.5.2. 2D Electrophoresis

Two-dimensional electrophoresis (2DE) was performed using the Immobiline polyacrylamide system, as previously described [26]. Immobilized nonlinear pH 3–10 gradient on strips 18 cm in length (Cytiva, Uppsala, Sweden) were employed in the first dimensional run carried out by Ettan™ IPGphor™ Manifold (GE Healthcare, Uppsala, Sweden) at 16 °C with the following electrical conditions: 200 V for 8 h, from 200 V to 3500 V in 2 h, 3500 V for 2 h, from 3500 V to 5000 V in 2 h, 5000 V for 3 h, from 5000 V to 8000 V in 1 h, 8000 V for 3 h, from 8000 V to 10,000 V in 1 h, and 10,000 V for 10 h for a total of 90,000 VhT. Analytical and MS-preparative strips were pre-rehydrated overnight with 350 µL of denaturing solution. Samples, added with 0.2% of carrier ampholytes for the analytical runs and 2% for the preparative ones, were loaded by cup at the cathodic ends of the IPGstrips. At the end of the first-dimensional run, strips were washed with deionized water and equilibrated with two buffers: the first composed of 6 M Urea, 2% *w*/*v* Sodium Dodecyl Sulphate (SDS), 2% *w*/*v* DTE, 30% *v*/*v* glycerol and 0.05 M Tris-HCl pH 6.8, for 12 min; the second one composed of 6 M Urea, 2% *w*/*v* SDS, 2.5% *w*/*v* Iodoacetamide, 30% *v*/*v* glycerol, 0.05 M Tris-HCl pH 6.8, and a trace of bromophenol blue, for 5 min. The second dimension was then performed at 40 mA/gel constant current on 9–16% SDS polyacrylamide linear gradient gels (size: 18 × 20 cm × 1.5 mm) at 9 °C. Analytical gels were stained with ammoniacal silver nitrate, while preparative gels underwent a mass spectrometry-compatible silver staining [8]; then, gels were digitized with Image Scanner III laser densitometer supplied with the LabScan 6.0 software (GE Healthcare). Two-dimensional image analysis was performed using Melanie 9 software (GeneBio, Geneva, Swiss). Gel comparison resulted in quantitative and qualitative protein differences, validated by statistical analysis.

Using Melanie 9 software, the ANOVA test was applied to compare the percentage of relative volume (%V) means of the 2DE protein spots among the groups. In particular, only differentially abundant spots with a *p*-value ≤ 0.05 and with at least a fold change of 2.5 in the ratio of the %V means, were considered differentially abundant.

#### 2.5.3. MALDI-TOF/TOF MS—Protein Identification

Differential electrophoretic spots were excised by MS-preparative gels and de-stained in a solution of 30 mM potassium ferricyanide and 100 mM sodium sulphate anhydrous. After 30 min in 200 mM ammonium bicarbonate (AB), spots were dehydrated in 100% acetonitrile (ACN). Spots were then rehydrated and digested in trypsin solution overnight at 37 °C. One microliter of the peptide solution was placed on the MALDI target, dried and covered with the matrix solution composed of 5 mg/mL α-cyano-4-hydroxycinnamic acid (CHCA) in 50% *v*/*v* ACN and 5% *v*/*v* trifluoroacetic acid (TFA) and dried again. MS analysis was performed with UltrafleXtreme™ MALDI-ToF/ToF instrument (Bruker Daltonics, Bremen, Germany) equipped with a 200 Hz smartbeam™ I laser in the positive reflector mode according to defined parameters: 80 ns of delay; ion source 1: 25 kV; ion source 2: 21.75 kV; lens voltage: 9.50 kV; reflector voltage: 26.30 kV; and reflector 2 voltage: 14.00 kV. The applied laser wavelength and frequency were 353 nm and 100 Hz, respectively, and the percentage was set to 46%. Final mass spectra were produced by averaging 1500 laser shots targeting five different positions within the spot. Spectra were acquired automatically, and the Flex Analysis software version 3.0 (Bruker, Bruker Corporation, Billerica, MA, USA) was used for their analysis and for assigning the peaks. The applied software generated a list of peaks up to 200, using a signal-to-noise ratio of 3 as the threshold for peak acceptance. Recorded spectra were calibrated using peptides arising from trypsin autoproteolysis as an internal standard. The resulting mass lists were filtered for contaminant removal: mass matrix-related ions, trypsin auto-lysis, and keratin peaks. Peptide Mass Fingerprinting (PMF) search was performed using MASCOT (Matrix Science Ltd., London, UK, http://www.matrixscience.com(accessed on 13 November 2024) setting up the following search parameters: *Homo sapiens* as taxonomy, Swiss-Prot/TrEMBL as databases, 50 ppm as mass tolerance, one admissible missed cleavage site, carbamidomethylation (iodoacetamide alkylation) of cysteine as fixed modification, and oxidation of methionine as a variable modification. The mass spectrometry proteomics data were deposited to the ProteomeXchange Consortium [24] via the PRIDE [25] partner repository with the dataset identifier PXD051390 (reviewer_pxd051390@ebi.ac.uk).

#### 2.5.4. Heatmap and PCA of Proteomic Gel-Based Results

Multivariate analysis was performed by uploading, on XLStat software version 2024.2.1 (XLSTAT-life Science-Paris, France), a matrix built with the %V of the differential spots obtained by differential gel-based analysis (rows) in each 2D gel (columns). In particular, PCA was performed by applying Spearman correlation to simplify the amount of data (%V variables) by linear transformation, visualizing the samples (2D gels) in a two-dimensional plane on the basis of the differential spot patterns. The clustering of protein spots by heatmap was performed using Ward’s clustering method and Euclidean distance.

#### 2.5.5. Enrichment Analysis of Gel-Based and Gel-Free Proteomic Results by MetaCore

Enrichment analysis was performed, submitting the accession number of the identified proteins to the MetaCore™ version 22.1 building tool (Clarivate Analytics, Philadelphia, PA, USA. Date: 18 April 2023). MetaCore has a manually annotated database storing exhaustive information about genes, proteins, chemical compounds, their interactions, molecular functions, and the processes involved. MetaCore enables powerful manual data analysis reporting composed of ontologies mapped to canonical pathways and networks. In order to build a hypothetical network connecting two experimental proteins directly or indirectly using one or more MetaCore database proteins, we used the algorithm’s “shortest path” that builds a network including only closely related proteins and introducing a maximum of one nonexperimental protein prioritized according to their statistical significance (*p* ≤ 0.001). Networks were visualized graphically as nodes (proteins) and edges (links between proteins), and relevant biological processes were then prioritized according to their statistical significance (*p* ≤ 0.001 and FDR) reporting the specific proteins involved.

### 2.6. Transcriptome Profiling

#### 2.6.1. Total RNA Isolation and Quality Assessment

EV pellet samples were resuspended in 350 µL Lysis Buffer RTL and proceeded by the extraction of RNA using the Qiacube automatic extractor following the manufacturer’s instructions (Qiagen, Hilden, Germany). RNA from each sample was eluted in 30 µL of water.

The integrity and quantity of the extracted EV RNA were analyzed and confirmed at first through the Qubit™ RNA High Sensitivity Assay (quantification range: 4–200 ng), used with the Qubit 4 Fluorometer (Invitrogen™, ThermoFisher Scientific, Waltham, MA, USA). Then, a more accurate study of the RNA quality was assessed using the Agilent 2100 Bioanalyzer instrument with the RNA 6000 Pico Chip (Agilent Technologies, Amstelveen, The Netherlands) according to the manufacturer’s protocol. After passing those quality controls, each RNA sample was used for cDNA library construction and subsequent RNA-Seq.

#### 2.6.2. Library Preparation and RNA-Seq

A total amount of 10 ng RNA per samples was depleted from rRNA with NEBNext rRNA Depletion kit v2 New En(New England Biolabs Inc., Ipswich, MA, USA) gland Biolabs Inc., Ipswich, MA, USA) according to the manufacturer’s instruction; then, libraries were prepared using NEBNext Ultra II Directional RNA library prep kit for Illumina with Unique Dual Index UMI adaptors (New England Biolabs Inc., Ipswich, MA, USA) following the manufacturer’s protocol. RNA fragmentation time, adaptor dilution, and PCR cycles were optimized according to protocol suggestions. Quality controls of individual libraries were assessed with Agilent DNA High Sensitivity Kit on Agilent 2100 Bioanalyzer, and quantification and normalization were performed with Qubit™ DNA High Sensitivity Assay on Qubit 4 Fluorometer. RNA-seq was performed in 100 cycles of single-end sequencing on Illumina NovaSeq 6000 platform (Illumina Inc., San Diego, CA, USA) at a sequence depth of 30 million of reads per samples.

#### 2.6.3. RNA-Seq Data Alignment and Identification of DEGs

Raw FASTQ files were inspected for quality with FastQC (v0.11.9) [27] and MultiQC (v1.13) software [28]. Sequencing reads were trimmed for adapters, polyG and polyA with fastp (v0.23.2) tool [29], then aligned to GENCODE 42 reference genome (GRCh38.p13, primary assembly) [30] with STAR (v2.7.10b) aligner [31], using basic workflow and ENCODE standard options for bulk RNA-seq pipeline, as reported in the user manual. Aligned reads were indexed with SAMtools (v1.11) [32], then deduplicated with UMI-tools (v1.1.2) software [33] using a minimum mapping quality of 10 and splicing plus read length as criteria for uniqueness. Deduplicated BAM files were inspected for quality with QualiMap2 (v.2.2.2-dev) software [34] and used to quantify strand-specific gene expression with the featureCounts (v2.0.1) tool [35]. The final count matrix was used to determine the differential gene expression of detected protein-coding transcripts among groups. All subsequent analyses were performed with DESeq2 (v1.38.3) [36] and tidyverse (v2.0.0) [37] packages of R (v4.2.2) statistical environment inside RStudio (v2022.7.2.576) IDE [38]. Only features with at least 10 mean counts in any of the experimental groups were kept. Intersections between retained features were computed and plotted with VennDiagram (v1.7.3) package [39]. Pearson correlation of average gene expression among groups was assessed on log2-transformed normalized counts (after addition of 1 pseudocount) and plotted with GGally (v2.1.2) [40] and ggpointdensity (v0.1.0) [41] packages. Dimensionality reduction with PCA and unsupervised hierarchical clustering based on Pearson distance were performed to assess sample similarity and were plotted using ggrepel (v0.9.3) [42] and pheatmap (v1.0.12) [43] packages. Volcano plots of differentially expressed genes (Wald test FDR < 0.01) were plotted with EnhancedVolcano (v1.16.0) package [44]. Intersections of up- or downregulated genes among groups were computed and plotted with ggupset (v0.3.0) package [45]. Gene enrichment analysis was performed with Enrichr (v3.1) package [46] on available Gene Ontology [47] and KEGG databases (v2021) [48]. All software and tools used for transcriptomic analysis are listed in Table S1.

### 2.7. Gene Expression Analysis by Droplets Digital PCR (ddPCR)

Gene expression for selected candidates was assessed using One-Step RT-ddPCR Advanced Kit for Probes assay (Bio-RAD Laboratories, Hercules, CA, USA) (Table S2). The preparation of the reaction mix for 1 sample is as follows: 5.5 µL Supermix, 2.2 µL Reverse Transcriptase, 1.1 µL mM DTT, 1.1 µL Probe FAM, 1.1 µL Probe HEX, 8 µL H20 and 3 µL RNA and proceeded with the droplet generation following the manufacturer instructions (Bio-RAD Laboratories, Hercules, CA, USA). The 96-well PCR plate was placed in a thermal cycler with the following cycling conditions: 1 h at 42 °C, 10 min at 95 °C, 30 s at 95 °C, 1 min at 58 °C, 10 min at 98 °C. Finally, the 96-well plate was loaded into the QX200 Droplet Reader (Bio-Rad, CA, USA), to identify the fluorescence intensity of each droplet for FAM/HEX fluorophore. Data were analyzed using the QuantaSoftTM Analysis Pro software, version 1.0 (Bio-Rad, CA, USA). The expression of the target genes was normalized based on the expression of *Glyceraldehyde 3-phosphate dehydrogenase* (*GAPDH*) and *Ro60-Associated Y4* (*RNY4*).

Statistical analysis, for gene expression, was performed using the GraphPad Prism 5.0 (GraphPad Software, San Diego, CA, USA). Statistical significance was evaluated by using nonparametric tests. Differences among groups of data were tested by the Mann–Whitney test for two groups or Kruskal–Wallis one-way analysis of variance followed by the Dunn’s post hoc test for multiple comparisons. Correlation was determined by using Spearman’s test. Statistical significance was set at *p* ≤ 0.05.

## 3. Results

### 3.1. Assessment of EV Recovery and Enrichment from Seminal Plasma

A total of 39 subjects were enrolled in this study. The mean age was 36.2 ± 6.3 years (range: 20–45 years); the BMI ranged between 18 and 25 (normal weight). For this study, subjects were characterized based on principal sperm parameters such as concentration, total motility, vitality, and morphology, according to WHO 2021 guidelines [18]. Seminal fluids were collected from adult men with Normozoospermia (NORMO, n = 15), OligoAsthenoTeratozoospermia (OAT, n = 12) and Azoospermia (AZO, n = 12); semen characteristics are reported in Table S3. The OAT and AZO are nonobstructive conditions.

**Figure 1:**
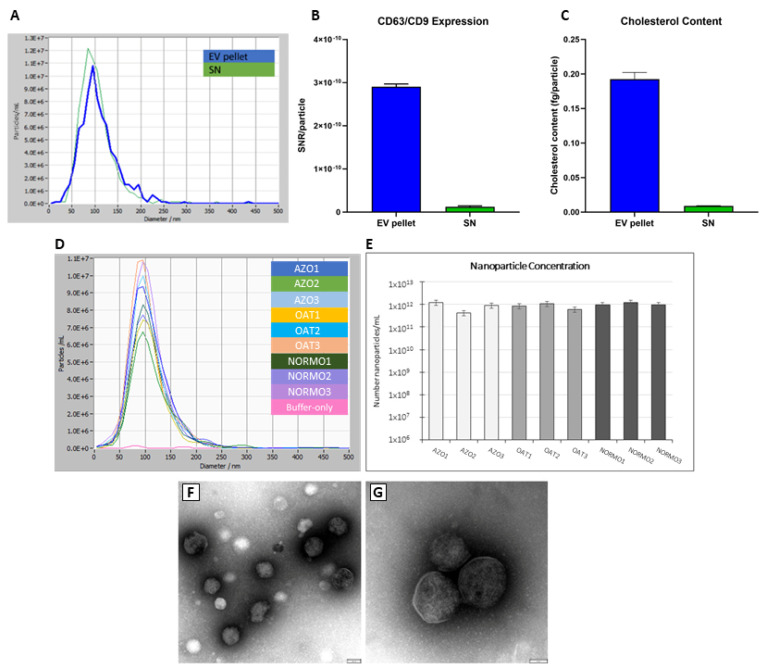
Characterization of EVs isolated by EV-GAG-based protocol from seminal fluids. The assessment of EV content was first performed on three proband samples from three NORMO subjects (**A**–**C**); (**A**) NTA-estimated particle size distributions, (**B**) cholesterol content (expressed in fg per particle), (**C**) tetraspanin CD63/CD9 expression, expressed as signal-to-noise ratio (SNR). EV-pellet indicates pellet obtained by EV-GAG-mediated precipitation from seminal fluid, and SN- indicates post-precipitation supernatant. (**D**,**E**) NTA was used to compare the size and concentration of particles isolated from normozoospermic (NORMO), oligoasthenoteratozoospermic (OAT), and azoospermic (AZO) subjects. (**F**,**G**) Ultrastructural characterization of EVs from NORMO samples by TEM.

In order to recover EVs from seminal plasma samples, we have opted for a precipitation-based protocol employing the commercially available reagent EV-GAG^®^ (Hansabiomed Life Sciences) with a mode of action that is based on interactions with EV surface-displayed glycosaminoglycans (GAGs) [20]. We have previously assessed the optimal precipitation reagent-to-sample volume ratio in order to obtain the best EV yield and purity trade-off. The scheme of in-house optimized protocol used for EV isolation is reported in the Section 2 (Figure S2). Before proceeding with the comparative assessment of samples derived from subjects with different seminal parameters, we used several proband samples from the collected cohort of NORMO subjects to qualify our isolation protocol and confirm the enrichment of EVs in recovered pellets (Figure 1).

The isolated EVs (pellet), and the EV-depleted controls (supernatant of the EV-GAG precipitation step), obtained from three NORMO individuals, were characterized for their EV content (Figure 1A–C). Reliable protein quantification was not feasible due to the interference of the precipitation reagent with conventional assays such as BCA. Although NTA could be performed, it was not consistent with other independently measured EV parameters. NTA also demonstrated particles in the EV-depleted controls. The amount of nanosized particles was similar between an EV pellet and a supernatant (approximately 60% versus 40%) (Figure 1A). Although the size of particles peaked around 100 nm diameter in both sample fractions, a broad size distribution was observed. This was indicative of the presence of both smaller and larger objects, possibly due to precipitation reagent residues or aggregation events, respectively. There was a slight shift towards smaller sizes in EV-depleted controls. We observed comparable particle concentration ranging from 4.2 × 10^11^/mL to 1.3 × 10^12^/mL, and homologous size distribution, revolving around the predicted 100 nm peak, in all proband samples (Figure 1E), and subsequently, across the whole set of seminal fluid EV isolates from NORMO, OAT, and AZO (Figure 1D). We attempted Western blot analysis, but the EV content in the semen samples was too low to meet the sensitivity of this method (in our experience being 10^9^ vesicles) even after EV fraction pooling, further suggesting that NTA estimates might be misleading in determining the accurate EV concentrations.

On the other hand, a sandwich ELISA assay assessing the EV-displayed tetraspanins CD63 and CD9 showed 10-fold higher immunoreactivity in resuspended EV pellets with respect to respective EV-depleted controls (Figure 1C). In addition, the quantification of cholesterol in the isolated EVs showed an over 20-fold increased cholesterol content per particle with respect to the depleted controls (Figure 1B). This data supports the effective enrichment of EVs in the pellets obtained by using the isolation protocol. The structural identity of isolated EVs was confirmed by TEM analysis, evidencing the typical EV size and morphology across the NORMO samples (Figure 1F,G). We noted a vesicle-feature-characteristic round-shaped structure and a thin electron dense membrane, thus confirming that the EV-GAG^®^ precipitation method allows the recovery of intact vesicles. Vesicles are heterogeneous in size, with diameters ranging from 50 to 150 nm. Besides the polymer residues, the EV preparations appear pure.

### 3.2. Characterization of a Normal Protein Cargo in Sperm EVs

**Figure 2:**
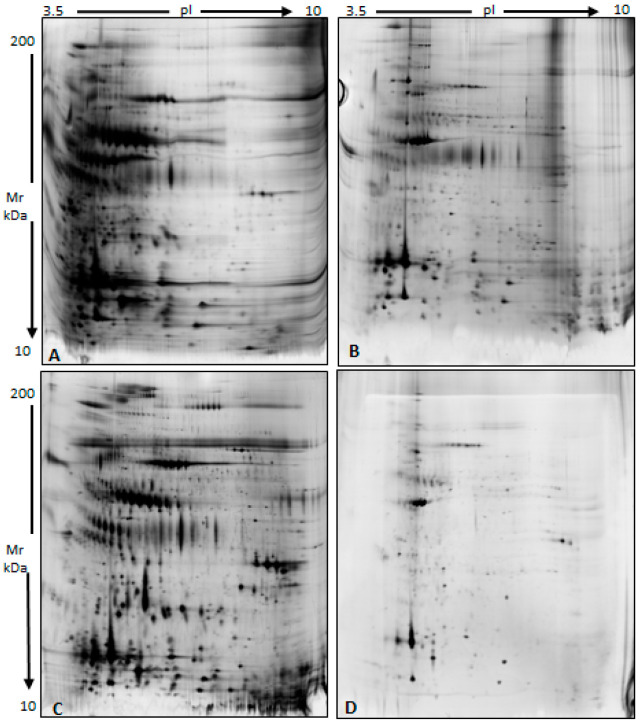
Representative 2D electropherograms from samples collected during the EV isolation protocol set-up. (**A**) EV pellet obtained without PBS washes; (**B**) EV pellet washed 3 times in PBS; (**C**) supernatant of the evGAG precipitation; (**D**) third PBS wash.

In order to evaluate the impact of the selected EV isolation protocol on the sample composition, specificity of EV isolation, and protein recovery rate [49], we ultimately used 2D electrophoresis to visualize the protein profiles of the NORMO EVs with respect to non-fractionated NORMO donor-derived sperm plasma samples. We could appreciate significant differences between the gel profiles of different fractions of the same seminal fluid sample when the isolated EV pellets, with or without washing with PBS (Figure 2), were compared to the residual fractions, namely the seminal fluid supernatant (EV depleted) and wash-aways. It is worth noting that all the EV pellets used for subsequent omics and gene expression analysis in the current study were washed extensively after isolation (see Section 2, Figure S2). As observed in Figure 2B, the electrophoretic map of the EV pellet washed three times with PBS does not show the typical chain of spots referred to as the albumin, transferrin, and immunoglobulins that are present in the electrophoretic map of the seminal plasma supernatant obtained after EV precipitation (Figure 2C) and are still visible in the original EV pellet obtained without washing (Figure 2A). This indicates that the final EV-purified sample is not contaminated by the most abundant seminal plasma proteins. At the same time, washing with PBS did not modify the presence of the characteristic protein spots in the gel (Figure 2B,C).

**Figure 3:**
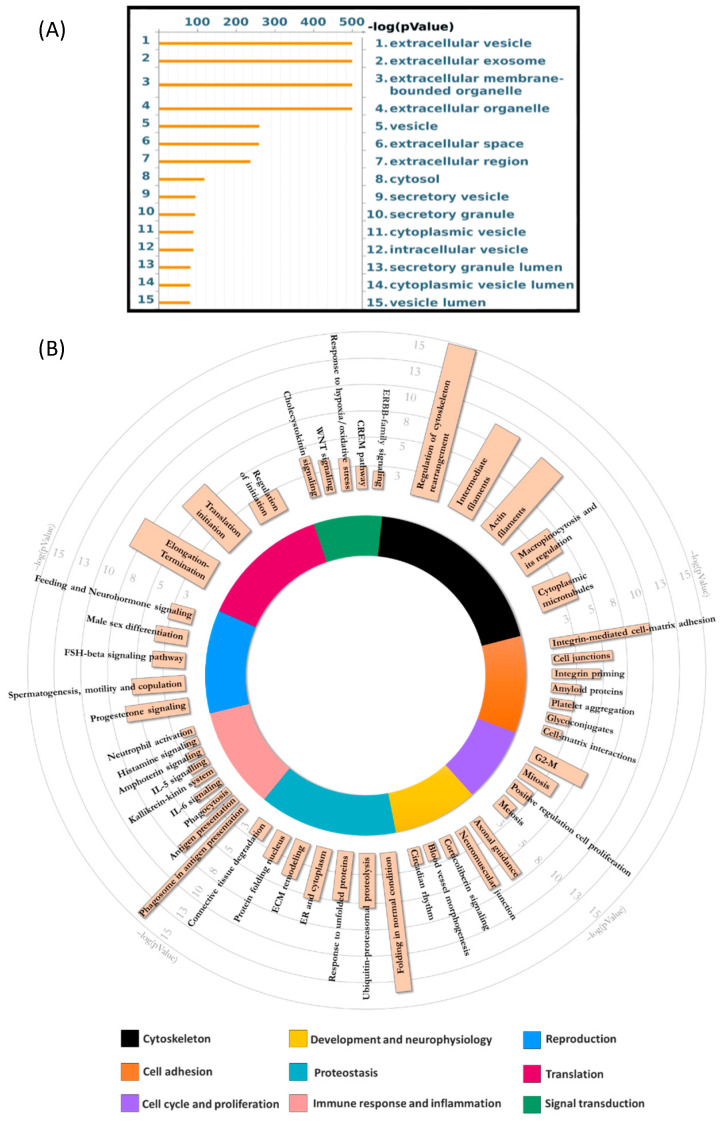
Enrichment analysis of NORMO EV protein cargo by Gene Ontology. (**A**) GO Cellular localization: representative scheme of the top 15 most abundant GO terms. The x-axis indicates the −log(*p*-Value), while the y-axis indicates different GO terms ordered by −log(*p*-value). (**B**) GO Biological Processes: visual representation of enriched GO Biological Processes (BP) terms for shotgun proteomic data of EVs isolated from human seminal fluid of NORMO subjects. Coloured bars constituting the circle graph represent the nine most enriched general BP terms. The length of the coloured bars indicates the mean of the −log(*p*-value) of all related specific BP terms. Salmon pink bars represent enriched specific BP terms, and the length of the bars indicate the −log(*p*-value) of the term. Therefore, the longer the bars, the higher the significance.

To characterize the proteomic content of seminal fluid EVs from NORMO samples, we combined and compared results from several proteomic approaches, both gel-free and gel-based ones. The gel-free approach by LC-MS/MS identified a total of 1133 proteins with an FDR value of *p* < 0.01 (Table S4). To understand the functions and the molecular pathways in which these proteins are involved, we performed an enrichment analysis by entering the accession numbers of the identified proteins into the MetaCore software (Clarivate Analytics). Available online: https://clarivate.com/cortellis/solutions/early-research-intelligence-solutions/ (accessed on 10 September 2024). Cellular localization GO analysis confirmed the association of identified proteins to EVs (Figure 3A).

As reported by the process network analysis (MetaCore), the proteins identified in NORMO sperm EVs are involved in numerous functions related to reproduction (Table S5, Figure 3B). These include cytoskeleton regulation, cell adhesion, cell cycle, development, and neurophysiology, proteostasis mechanisms, immune response and inflammation, progesterone signalling and spermatogenesis, protein translation, and signal transduction.

### 3.3. Differential Proteomic Analysis of EVs from Subjects with Different Sperm Parameters

**Figure 4:**
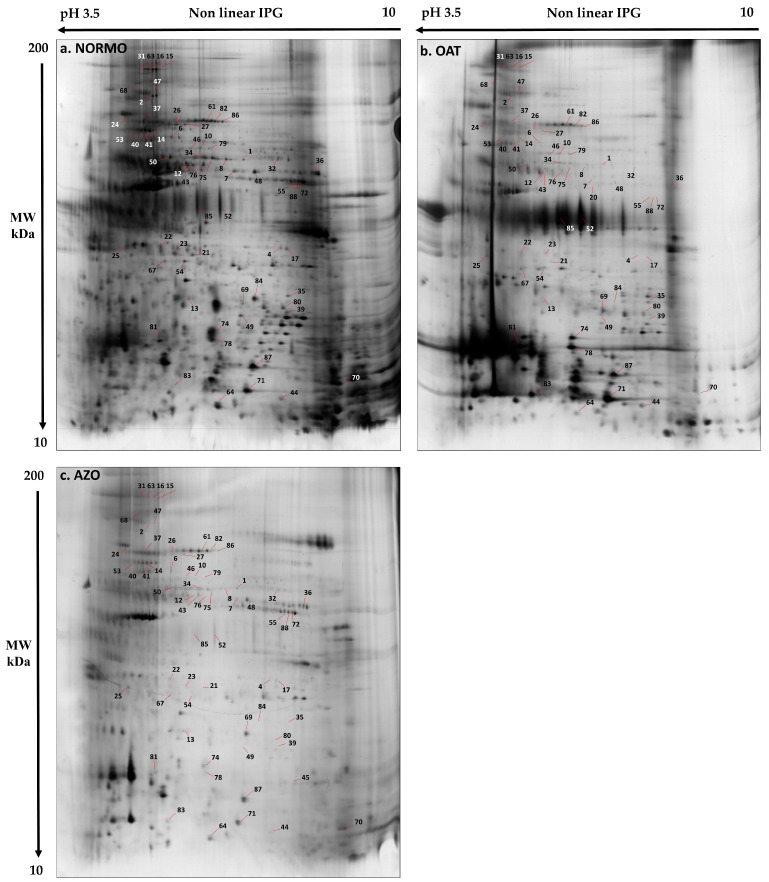
Proteomic differential analysis. Representative electropherograms of EV samples obtained from (**a**) NORMO, (**b**) OAT, and (**c**) AZO seminal fluid. Numbers indicate differential spots found by the comparative analysis.

A comparative 2DE analysis of NORMO, OAT, and AZO EV samples was performed to highlight differentially expressed protein species specific for each group (Figure 4). The gel images showed an average of 1690 spots, and the comparative analysis extrapolated a total of 80 differentially abundant spots according to the ANOVA test (*p* < 0.05), among the three conditions, with a fold change >2.5.

Mass spectrometry (MALDI-ToF–ToF/MS) of selected spots identified 47 proteins (enlisted in Table S6). Overall, differential proteomic analysis evidenced three groups of proteins: highly abundant proteins in NORMO EVs with respect to OAT and AZO (green), highly abundant proteins in OAT EVs with respect to NORMO and AZO (blue), and highly abundant proteins in AZO with respect to OAT (red); there are no proteins highly abundant in AZO with respect to NORMO.

**Figure 5:**
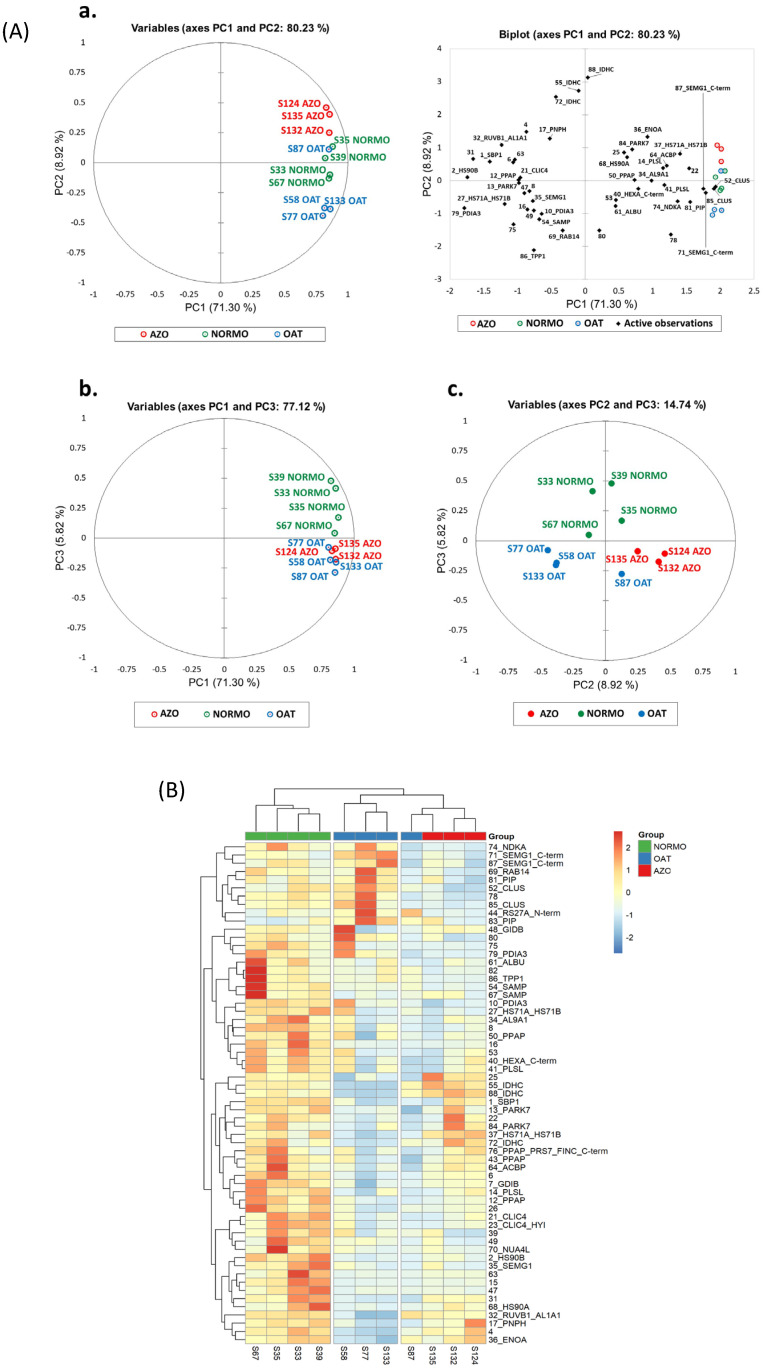
Proteomic differential analysis. (**A**) Principal component analysis performed by %V data of the differentially abundant spots. PCA by PC1 and PC2 and the most representative spots in sample distribution (panel **a**). PCA graph by PC1 and PC3 (panel **b**). PCA graph by PC2 and PC3 (panel **c**). (**B**) Heatmap analysis performed by %V data of the differentially abundant spots, by Euclidean distance. Green bar highlights NORMO samples, blue bars the OAT samples, and the red bars the AZO samples, as reported in the legend. Spot abundance ranges from red (highly abundant) to blue (lower abundant).

The principal component analysis (PCA) (Figure 5A) of most representative spots highlighted the clustering of OAT and AZO samples, revealing their similar protein pattern with respect to the NORMO condition. Samples were clustered close to each other, distributing the three conditions along PC2. PC1 shows a sufficient percentage of the total variability of the scatter plot. Figure 5A, panel b, shows the PCA distribution from another point of view (PC1: 72.31% and PC3: 5.44%) that highlights the peculiarity of the NORMO condition with respect to OAT and AZO, while panel c reports the distribution between PC2 (8.92%) and PC3 (5.44%). The NORMO condition remains distant from AZOO and OAT along PC2, while AZOO and OAT were mostly distinguished by PC3.

Coherently, the heatmap analysis gave us an instantaneous visualization of the spot abundance trend in the three conditions showing a prevalent protein abundance in NORMO samples that is well distinguished from OAT and AZO (Figure 5B). OAT and AZO samples belong to the same principal cluster suggesting a similar protein pattern behaviour. However, it is possible to appreciate two sub-clusters distinguishing OAT from AZO samples with one OAT that shows a differential protein profile similar to that of AZO.

**Figure 6:**
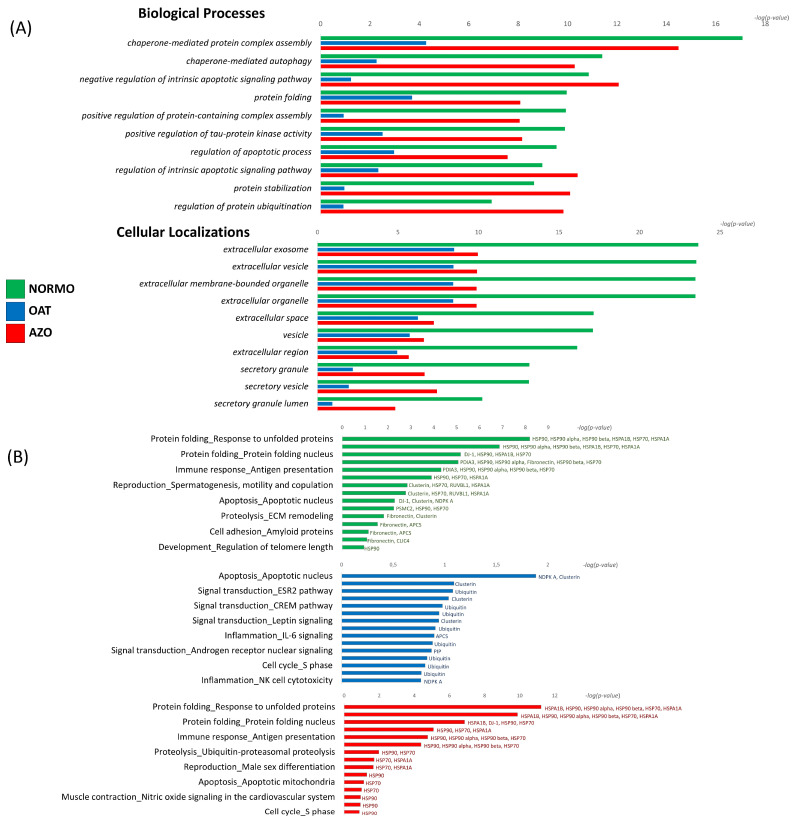
MetaCore analysis. (**A**) MetaCore enrichment analysis of the three groups of proteins (highly abundant in NORMO-green; highly abundant in OAT-blue; highly abundant in AZO-red). All groups reported the comparison of GO Biological Processes and Cellular Localization. (**B**) Individual Process network analysis by MetaCore of the highly abundant proteins in NORMO (green), OAT (blue), and AZO (red) EV samples, respectively. Process network terms were reported on the left part of the histograms, indicating the statistical significance. At the right side of the histograms were the proteins that mostly influenced that process network.

Differentially expressed proteins for NORMO, OAT, and AZO were subsequently uploaded onto MetaCore software(Clarivate Analytics). Available online: https://clarivate.com/cortellis/solutions/early-research-intelligence-solutions/ (accessed on 10 September 2024). for Gene Ontology (GO) Biological Processes and Cellular Localization enrichment analysis (Figure 6A), to obtain notions on the principal cellular mechanisms that can be reflected or affected by sperm EV content. Biological Process from GO analysis evidenced the significant involvement of highly abundant proteins in NORMO EVs in positive regulation of (chaperone-mediated) protein complex assembly, autophagy, protein folding, stabilization and ubiquitination, negative regulation of intrinsic apoptotic signalling pathway, and positive regulation of tau-protein kinase activity. For some of these terms, such as the regulation of apoptosis, protein stabilization, and regulation of protein ubiquitination, highly abundant proteins in AZO samples showed a higher significance with respect to proteins expressed in OAT and, to some extent, with respect to NORMO samples. Regarding the cellular localization from GO analysis, the statistical significance for all the reported terms regarding the Extracellular Vesicles, was higher for the abundant sperm EV proteins from NORMO, with respect to OAT and AZO samples.

Furthermore, we performed the “process networks” analysis by MetaCore, for the three group of proteins taken individually, in order to highlight peculiar molecular processes that the different EV cargos may modulate (Figure 5B).

This analysis confirmed the likelihood of the involvement of abundant proteins in NORMO sperm EV samples in protein folding. HSP90 and its proteoforms alpha and beta, HSPA1A and B, HSP70, and DJ-1, are the major proteins representative of this process. The proteins enriched in NORMO sperm EV samples involved in immune response involve PDIA3, HSP90 alpha and beta, HSP70, and fibronectin. As expected, NORMO Es abundant proteins such as clusterin, HSP70, RUVBL1, ASPA1A, fibronectin, and CLIC4 are involved in reproduction-related processes such a spermatogenesis, motility and copulation, male sex differentiation, and progesterone signalling. We also notice the enrichment of proteins involved in apoptosis (DJ-1, clusterin, NDPK A) and proteolysis (PSMC2, HSP90, HSP70, fibronectin, and clusterin) (Figure 5B-green/NORMO). The enrichment in fibronectin and APCS indicate the involvement in inflammation by IL6 signalling and cell adhesion by amyloid proteins, while HSP90 is implicated in numerous biological processes, including the regulation of telomere length.

The proteins enriched in OAT sperm EV samples did not display a high statistical value, indicating their involvement in process networks. However, clusterin and ubiquitin are involved in processes such as apoptosis, inflammation, signal transduction by ESR2, CREM pathways, and leptin signalling in addition to the regulation of the cell cycle and DNA damage (Figure 5B-blue/OAT). Another protein highly abundant in OAT sperm EVs, NDPA K, indicates its possible involvement in apoptosis and NK cell toxicity.

Regarding the highly abundant proteins found in AZO sperm EV samples (with respect to the OAT EVs), HSP90 and its proteoforms alpha and beta have the strongest potential impact on the modulation of biological processes such as protein folding and immune response, proteolysis, regulation of telomere length, muscle contraction by nitric oxide signalling, signal transduction by androgen receptor nuclear signalling, and the cell cycle (Figure 6B—in red). Interestingly, DJ-1, also called PARK7, is highly abundant in AZO with respect OAT EVs and indicates the possible role in the nucleus protein folding. Moreover, we observed similar network processes for NORMO and AZO EVs, but proteins in AZO were low in abundance with respect to the NORMO EVs, suggesting that AZO EVs could lose the functionality reported for the NORMO vesicles.

### 3.4. Differential Gene Expression Analysis of EVs from Subjects with Different Sperm Parameters

**Figure 7:**
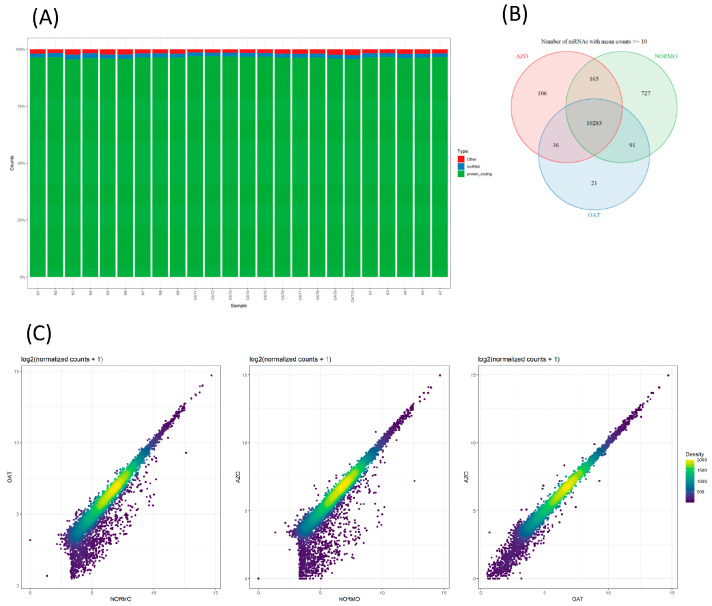
EV RNA cargo: a comparative transcriptomic analysis of RNA-Seq datasets from NORMO, OAT and AZO. (**A**) Histograms illustrate the differentially expressed RNA counts: in green protein-coding; in blue lncRNA; and in red other RNA. (**B**) Venn diagram highlighting the overlap of protein-coding transcripts among the three independent datasets. (**C**) Visualization of transcriptome differences, by scatter plots, in EVs between NORMO and OAT; NORMO and AZO; OAT and AZO. Values depicted are the log 2 transformation of cross-sample normalized RNA-seq read counts + 1 [log_2_(N + 1)]. The colour gradient, from purple to yellow, shows the density of points: yellow represents areas with the highest density of genes (many genes with similar expression levels in both conditions), while purple indicates lower density.

The sperm EV isolates from the same NORMO, OAT, and AZO sample cohort were in parallel analyzed for the composition of their RNA cargo. The RNA isolated from EV pellets was sufficient to yield about ~28 M raw reads per sample during the RNA sequencing run, with an average of ~26 M aligned reads per replicate, which is close enough to the 30 M specified in the experimental guidelines developed by the ENCODE consortium to define optimal bulk RNA sequencing experiments. Unfortunately, the deduplication step drastically reduced this number of reads to ~3 M. This is not unusual for a very low input RNA samples that need a high number of PCR amplification cycles at initial steps and are likely to produce a high number of PCR artefacts. Approximately 90% of deduplicated reads were attributed to human genes using GENCODE annotation on GRCh38 reference genome, obtaining ultimately an average of ~2.6 M counts per sample. The whole count matrix comprised a total of 35,056 different detected features, most of which were very lowly expressed. Most reads were associated with protein-coding transcripts (Figure 7A). After first filtering out the non-protein-coding transcripts, and then all the transcripts with low counts (lower than 10), the number of features dropped to 17,724 and 11,429, respectively. Of the latter, 10,283 were commonly detected in all sample groups (Figure 7B).

The correlation of gene expression between OAT and AZO groups is very strong (R = 0.990), with observed clear tendency of those two groups to show a general lower expression with respect to NORMO group (R NORMO/OAT = 0.952; R NORMO/AZO = 0.925) (Figure 7C).

**Figure 8:**
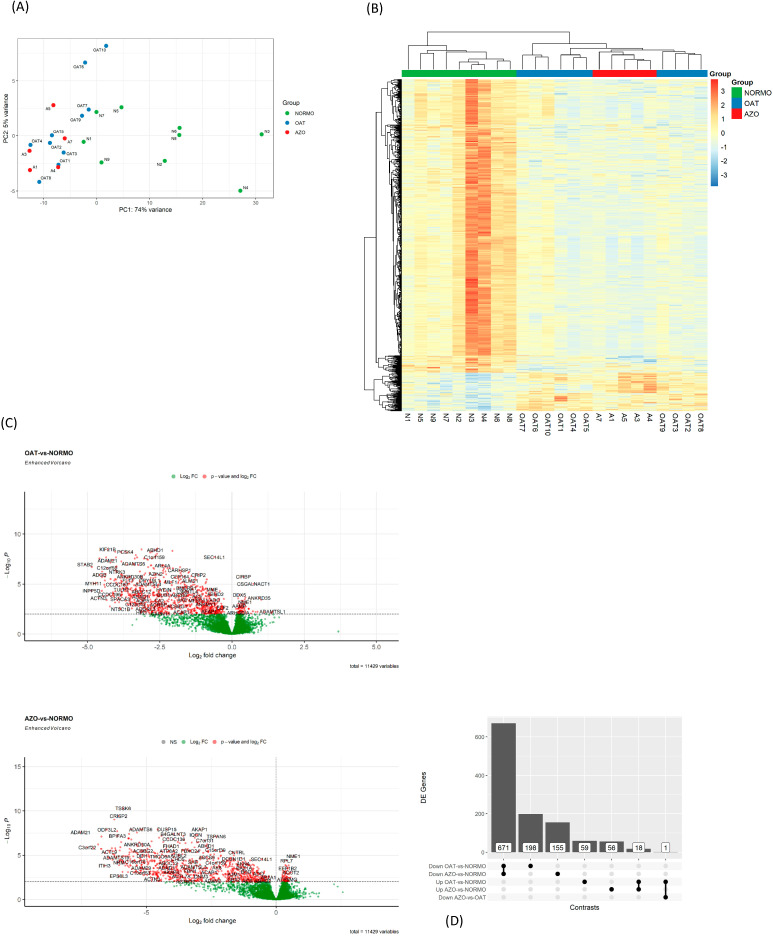
Differential gene expression between NORMO, OAT, and AZO. (**A**) Principal component analysis (PCA) showing distinct separation of NORMO with respect to the other two groups. (**B**) Heatmap with hierarchical clustering represents the expression levels from three groups: AZO (red), OAT (blue), and NORMO (green). The colour scale indicates gene expression intensity, with red representing higher expression and blue indicating lower expression. (**C**) The Volcano plot contrasting gene expression between OAT and NORMO (upper panel) and AZO vs. NORMO (lower panel) groups. The log2 fold changes on the x-axis show the magnitude of expression differences, while the −log10 *p*-values on the y-axis indicate statistical significance. Genes that are significantly differentially expressed, surpassing set thresholds for fold change and *p*-value, are labelled and coloured to stand out from non-significant ones (green) The fold change threshold has been set to zero. (**D**) The image presents a bar chart displaying the number of differentially expressed (DE) genes between three study groups: AZO, NORMO, and OAT. The chart shows comparisons between groups, indicating the direction of expression change. The tallest bar represents many genes downregulated in OAT compared to NORMO, while the smallest bar indicates a single gene downregulated in AZO compared to OAT.

Principal component analysis (PCA) (Figure 8A) and unsupervised hierarchical clustering (Figure 8B) fully agree in separating the whole NORMO group in a dedicated cluster, while AZO samples seem to form a subcluster of the major OAT group. Although some NORMO samples (e.g., N3 and N4) show relatively higher expression levels, this can be attributed to minimal inter-individual variability in EV content, which is a well-documented phenomenon in EV research. Several studies have shown that even in healthy individuals, EV cargo, such as proteins or small RNAs, can exhibit considerable biological variability, influenced by physiological factors and intra-subject dynamics [50]. Despite this variability, the overall expression trend remains consistent across the NORMO group. Differential gene expression analysis confirmed the trend of the general downregulation of AZO and OAT groups with respect to NORMO (Figure 8C,D), with several acknowledged: some members of the *ADAM* (A Disintegrin And Metalloprotease) gene family encodes a diverse group of transmembrane cell-surface proteins with adhesion and proteolytic functions; *CRISP2* (Cysteine-Rich Secretory Protein 2) that plays an important role in the morphology and motion of male ejaculated spermatozoa. The extreme similarity between OAT and AZO gene expression impaired the differential expression analysis between these two groups at the *p*-value adjustment step: the *p*-value distribution was nearly flat, lacking the expected peak of highly significant low *p*-values. This yielded a strong inflation of FDR values, leaving only one gene surviving, *CSGALNACT1*, higher in OAT versus AZO and, interestingly, contemporarily higher in OAT versus NORMO comparison as well.

**Figure 9:**
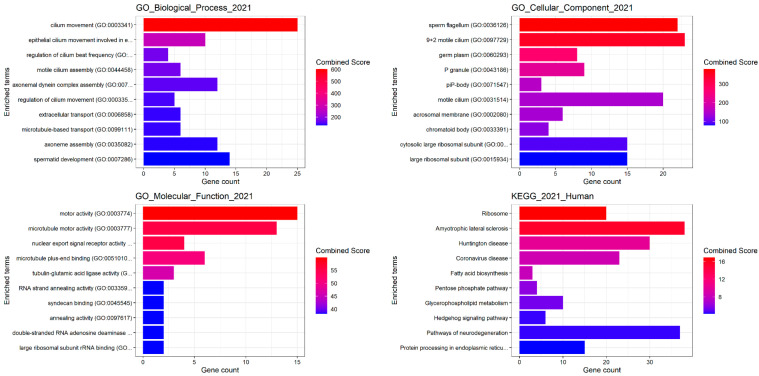
Gene enrichment analysis for the biological process ontology (GO) terms. The y-axis lists the GO terms. The top 100 differentially expressed genes (DEGs) were included. The x-axis quantifies the gene count associated with each term. The bars are coloured based on a combined score, indicated by the heatmap legend on the right, with red denoting higher scores and blue representing lower scores. KEGG pathway. Chart from a KEGG pathway enrichment analysis. The enriched terms along the y-axis represent various biological pathways. The x-axis shows the gene count that correlates with each pathway.

The gene enrichment analysis on differentially expressed genes showed important GO for Biological processes, cellular components, and molecular functions especially involved in sperm motility. The top 100 differentially expressed genes (DEGs) were included in the analysis (Figure 9).

**Figure 10:**
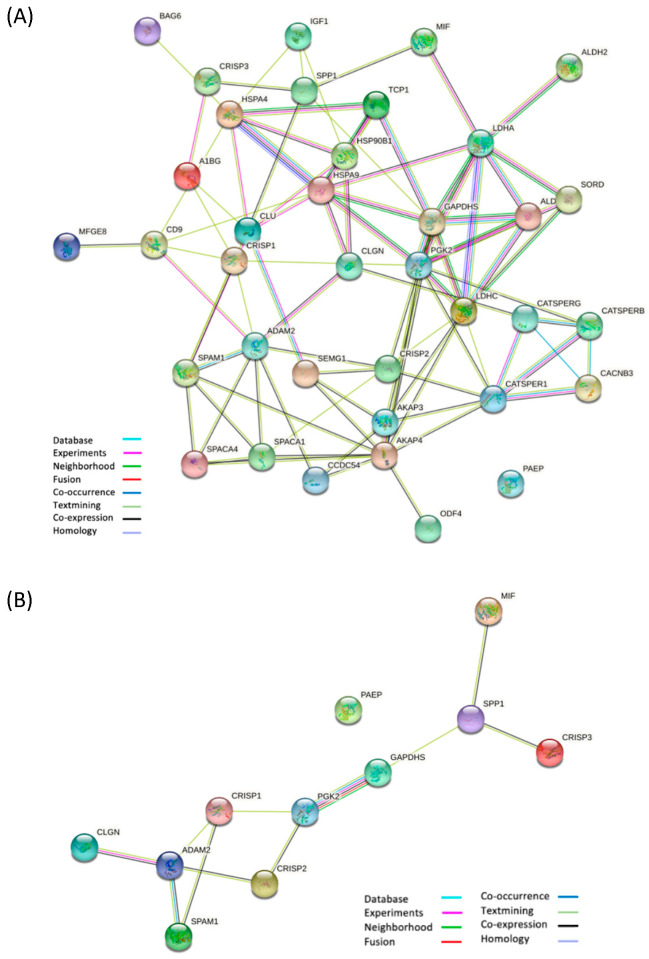
Network of interactions of proteins involved in the fertilization pathway generated by STRING. (**A**) A general overview. Specifically Alpha-1B-Glycoprotein (A1BG), Disintegrin and metalloproteinase domain-containing protein 2 (ADAM2), A-Kinase Anchor Protein 3, 4 (AKAP3, AKAP4), Aldehyde dehydrogenase 2 (ALDH2), Aldolase A (ALDOA), BAG cochaperone 6 (BAG6), Voltage-dependent L-type calcium channel subunit beta-3 (CACNB3), Cation channel sperm-associated protein 1, beta, gamma (CATSPER1, CATSPERB, CATSPERG), CD9 antigen (CD9), Calmegin (CLGN), Clusterin (CLU), Cysteine-rich secretory protein 1, 2, 3 (CRISP1, CRISP2, CRISP3), Glyceraldehyde-3-phosphate dehydrogenase, testis-specific (GAPDHS), Heat shock protein family A member 4 (HSPA4), Endoplasmin (HSP90B1), Stress-70 protein, mitochondrial (HSPA9), Insulin-like growth factor 1 (IGF1), Lactate dehydrogenase A, C (LDHA, LDHC), Lactadherin (MFGE8), Macrophage migration inhibitory factor (MIF), Outer dense fibre protein 4 (ODF4), Glycodelin (PAEP), Phosphoglycerate kinase 2 (PGK2), Sperm acrosome membrane-associated protein 1, 4 (SPACA1, SPACA4), Semenogelin 1 (SEMG1), Sorbitol dehydrogenase (SORD), Coiled-coil domain containing 54 (CCDC54), Sperm Adhesion Molecule 1 (SPAM1), Osteopontin (SPP1), and complex protein 1 subunit alpha (TCP1) are shown. (**B**) Network of selected and investigated interactions of ADAM2, CLGN, CRISP1, CRISP2, CRISP3, MIF, GAPDHS, PGK2, SPAM1, SPP1, and PAEP proteins generated by STRING. Each node represents a protein, while edges delineate the type of interconnection (network caption).

To validate the results obtained by RNA-Seq, we went to highlight the differences in gene expression in the three groups (NORMO vs. OAT vs. AZO) by ddPCR analysis. A preliminary in silico analysis was the starting point of our study. To this end, we selected from the literature proteins with a pivotal role in sperm maturation, acquisition of sperm fertilizing ability, and, ultimately, fertilization. Known and predicted functional links among them were assessed on the STRING database (Figure 10A). The associations in STRING include direct (physical) interactions, as well as indirect (functional) interactions; both are specific and biologically meaningful. All examined proteins showed strong interactions with each other except for PAEP, a protein with very important roles in fertilization, but surprisingly, the only one not to interact directly with any of the above proteins. Interactions among proteins belonging to the CRISP family (CRISP1, CRISP2, CRISP3), proteins responsible for sperm-egg binding and interaction with the oocyte-ZP (ADAM2, CLGN, SPAM1), sperm-specific glycolytic enzymes (GAPDHS, PGK2), and proteins involved in the regulation of sperm motility (MIF, SPP1) were analyzed in more detail. Moreover, PAEP, with well-known sperm capacitation inhibitory activity, was further examined with the aim to assess the effective lack of interactions with other proteins having a similar function. We conducted a gene expression analysis focused on a string of specific biological pathways related to the male reproductive tract (Figure 10B), including CRISP1-2-3, ADAM2, CLGN, PAEP, SPAM1, GAPDHS, PGK2, MIF, and SPP1.

**Figure 11:**
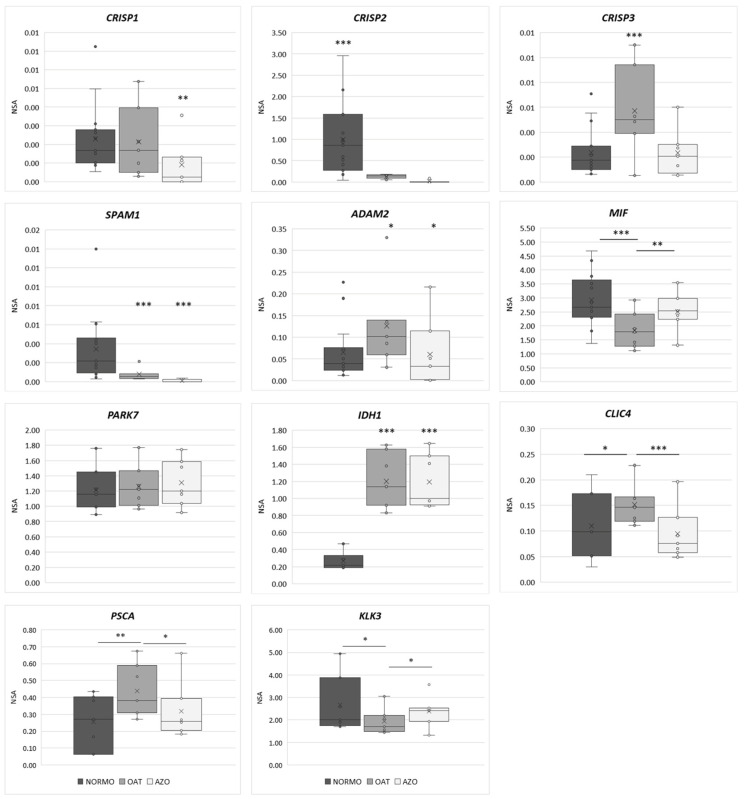
Gene expression analysis by ddPCR. Relative expression of Cysteine Rich Secretory Protein 1-2-3 (CRISP1-2-3), Sperm Adhesion Molecule 1 (SPAM1), ADAM Metallopeptidase Domain 2 (ADAM2), Macrophage Migration Inhibitory Factor (MIF), Parkinsonism-associated deglycase (PARK7), Ocitrate dehydrogenase (NADP(+)) 1, cytosolic (IDH1), Chloride intracellular channel 4 (CLIC4), Prostate stem cell antigen (PSCA), and Kallikrein-related peptidase 3 (KLK3) in seminal EVs of normozoospermic subjects. Normalization of target genes was performed using GAPDH and RNY4 as reference genes. Gene expression was performed by ddPCR. Graphical diagrams are plotted as box-whisker plots, where boxes show the interquartile range with median and mean values, and whiskers represent min and max confidence intervals. Outliers, plotted as individual dots, represent out-of-range values. * *p* < 0.05; ** *p* < 0.01; *** *p* < 0.001.

The transcript levels were normalized against reference genes, *GAPDH* and *RNY4*, known for stable and constitutive expression in various tissues. The relative expression levels of all investigated gene are shown in Figure 11.

*HSPA4*, a specific marker for EVs, showed no significant variation among the three samples. This result of this EV marker concords with the data obtained by NTA, which state that the concentration of the EVs does not change depending on semen parameters.

*CRISP1* and *CRISP3* were associated with seminal EVs, showing comparable levels, with an occasional outlier. In contrast, *CRISP2* displayed markedly higher expression levels compared to *CRISP1* and *CRISP3*, despite belonging to the same gene family. In the analysis of *MIF* expression levels among the three groups, it was notable that NORMO subjects displayed the highest expression of the latter. This difference was highly significant compared to OAT (*p* < 0.001). Conversely, OAT subjects showed the lowest expression of this gene, and there was a significant difference compared to AZO (*p* < 0.01). Interestingly, *CLGN*, *PAEP*, *SPP1*, and *PGK2* were expressed in the NORMO group, whereas OAT and AZO displayed no detectable expression of these genes.

We observed remarkably statistically uniform expression levels of *PARK7*. The selection of genes studied for their expression, guided by prior proteomic analyses that indicated a significant difference between the three groups, exerted a similar profiling in gene expression analysis as well.

In our comparative analysis of gene expression between NORMO, OAT, and AZO, we observed notable differences on the markers of prostasome: KLK3 and PSCA. The expression of *PSCA* was significantly higher in OAT compared to AZO (*p* < 0.05). Notably, when comparing OAT to NORMO, the difference in the expression was even more pivotal, with a higher level of significance (*p* < 0.01). These findings suggest that *PSCA* and *KLK3*, which are markers of the EVs, indicate that, throughout different conditions of male seminal parameters, the contribution of EVs is produced by different parts of the male reproductive tract.

## 4. Discussion

The examination of the factors influencing sperm quality and male fertility, along with the discovery of molecular indicators facilitating precise and accessible diagnosis of various male infertility conditions, has long been a central focus in reproductive medicine. Current research has primarily focused on analyzing spermatozoa, seminal plasma, and, in more invasive cases, testicular tissue through omics-based studies. Seminal plasma, in particular, holds promise as a non-invasive source for investigating sperm fertilization potential. Several studies reported evidence of a correlation between the proteome of seminal fluid and the alteration of sperm parameters [1,51]. It has been recognized that seminal plasma can influence the spermatozoa behaviour by instigating substantial modifications in the sperm membrane, mediated also through binding of specific proteins. The presence of certain bioactive molecules within seminal plasma is essential for promoting the sperm motility, facilitating capacitation, and creating a conducive immune environment within the uterus for the sperm transit. Consequently, the exploration of biomarkers related to male fertility within the bioactive molecules of seminal plasma is a pertinent area of research. These bioactive molecules may exist either freely in seminal plasma or enclosed within EVs.

In this study, we have first set up a robust and reliable protocol for EV isolation from semen samples, with the subsequent multiparameter and quantitative characterization of isolated vesicles. We have further characterized the transcriptomic and proteomic profile of EVs isolated from NORMO, OAT, and AZO men and confirmed their potential value as a proxy for a male fertility status. The in-depth morphological characterization of EVs recovered from NORMO subjects and from men with altered semen parameters (both OAT and AZO) highlighted that the overall concentration and size of the collected EVs does not vary significantly across different cohorts of subjects. Although the overall proteomic composition featured common protein species, the differential proteomic analysis highlighted significantly altered amounts and proportions of several proteins. NORMO EVs overall contained the highest amount of proteins with respect to OAT and AZO. On the other hand, AZO EVs presented the lowest content of a majority of protein species except for several proteins such as SBP1, HS90A/B, PARK7, PNPH, ENOA, HS71A/B, PPAP, IDHC, and ACBP, that displayed higher abundancy in AZO samples with respect to OAT. Our results suggested that EVs in the vesicular population of seminal plasma are normally released by numerous cells in the male reproductive tract, but their content is impoverished in OAT, and especially in AZO subjects, with respect to a normal (NORMO) condition, as also suggested by enrichment analysis. Indeed, proteins in AZO vesicles belong to the same process networks of the NORMO proteins but are present in smaller quantities, suggesting that AZO EVs could lose the functionality reported for the NORMO vesicles. Multivariate analysis showed the differential EV protein pattern distinctive of the three sample groups. A GO analysis of biological processes associated with differentially expressed EV proteins suggested that those that were found to be altered in OAT and in AZO EVs were almost exclusively involved in mechanisms that orchestrate reproductive processes, such as protein folding, inflammation, immune responses, proteolysis, and signal transduction. It is acknowledged that all these mechanisms normally cooperate for a successful pregnancy, that is indeed precluded by sperm alterations and deficiency in OAT and AZO subjects.

Fertilization in mammals is a complex process involving the interaction of male and female gametes. CRISPs play a crucial role in making this interaction possible and are highly expressed in male reproductive tissues, including the testis, prostate, and epididymis [52]. CRISPs mainly expressed in the male reproductive tract are CRISP1 and CRISP3 synthesized by the epididymis, CRISP2 of testicular origin, and CRISP3 originating from the prostate and seminal vesicles. CRISP1 is associated with the sperm surface with two different affinities during maturation and is responsible for regulating the capacitation process and sperm–ZP interaction. CRISP2 and 3 are also involved in sperm–egg interaction, supporting the existence of functional redundancy and cooperation between homologous proteins that ensures successful fertilization. CRISP proteins, therefore, accompany spermatozoa along their transit through the male and female reproductive tracts and could be excellent candidates for future research on infertility and contraception [53]. Spermatozoa leaving the testis contain CRISP2 within the head and the tail. During the process of sperm maturation within the epididymis, CRISP1 and CRISP3 bind weakly or strongly to the sperm surface transported to sperm by epididymosomes or in a soluble form. In the female reproductive tract, weakly associated proteins are partially released from sperm cells during capacitation, whereas the intracellular or tightly bound population remains in the cells and relocalizes to the equatorial segment of spermatozoa that have undergone the acrosomal reaction. Thus, each sperm CRISP protein participates in more than one stage of fertilization and cooperates with other CRISP homologs at each stage of fertilization. Based on published data, the tissue-specific expression of CRISP1 in the epididymis and CRISP2 in the testis provides important information about the source and role of EVs released along the male reproductive tract. Our data, showing higher levels of *CRISP2* in the seminal fluid of those in the NORMO group, which emphasizes the role of testicular EVs, supports this. The significance of describing the EV populations found in seminal fluid in relation to their original tissue is highlighted by these findings. This characterization could show the dynamic molecular changes that spermatozoa undergo as they pass through the male reproductive system and clarify the precise contributions of various reproductive segments to the composition of EVs.

Our data identified specific proteins of which downregulation, evidenced in EVs from OAT and AZO subjects, may result in the failure in supporting successful pregnancies. These results confirm and expand prior emerging literature which suggest that seminal fluid EVs may not only influence spermatozoa function but also exert modulatory effects on the female reproductive tract, contributing to multiple physiological processes that collectively support a successful pregnancy [54,55]. For instance, proteomic results, as well as transcriptomic readouts, reported a low abundance of PARK7 in OAT EVs with respect to NORMO and AZO EVs. PARK7 protein has been extensively studied in the context of neurodegenerative disorders, and it is known to be expressed not only in the brain but also in other tissues and organs, including male epididymis and testis. Although its precise function in sperm is not fully understood, PARK7 is associated with responding to oxidative stress. Studies in humans have indicated a positive correlation between PARK7 and aspects of sperm health such as plasma membrane integrity, motility, and superoxide dismutase activity. Furthermore, previous investigations have explored PARK7’s relevance to male fertility, revealing its downregulation in conditions like oligozoospermia, asthenozoospermia, and varicocele [56,57].

Furthermore, the concordance between proteomic and transcriptomic data is evident in the example of the downregulation of CLIC4 at both the protein and mRNA levels, observed in EVs from OAT and AZO subjects, compared to NORMO. This protein is found in spermatozoa mainly confined to the anterior perimeter of the sperm head and the flagellum. CLIC proteins are known to interact with PP1Y2, a key enzyme involved in regulating sperm maturation and motility, and may play a role in signal transduction [58]. The presence of CLIC proteins within the sperm head implies a potential involvement in crucial gamete fertilization events, such as sperm-egg membrane fusion and the acrosome reaction. Adding to their significance, CLIC4 is expressed at all the stages of development in Xenopus laevis Embryo, suggesting an essential role during early development. This raises the possibility that CLIC4 proteins may serve as cargo destined for the egg itself, indicating a broader functional relevance beyond the specific context of sperm function. The identification of CLIC4 within the EVs from NORMO but not from OAT and AZO subjects may indicate the multilevel involvement of sperm EVs in supporting the fertilization process.

## 5. Conclusions

Seminal EVs secreted along the male reproductive tract have been demonstrated to be involved in the process of sperm maturation and could represent a new appealing therapeutic and diagnostic tool in the field of human reproduction for male fertility/infertility.

In this study, an exhaustive omics analysis (transcriptomic and proteomic) was carried out on the EVs derived from human seminal plasma, providing a comprehensive understanding of the cargo content, and regulatory biological networks. Our findings strongly support the evidence that seminal EV-associated protein networks may be connected to the molecular mechanisms involved in sperm maturation and motility. This study contributes to elucidating the key role of EVs in the paracrine communication regulating spermatogenesis. A full understanding of these pathways not only suggests potential mechanisms regulating male fertility but also offers new insights into the development of diagnostic tools targeting male reproductive disorders.

## Data Availability

The original contributions presented in this study are included in the article/Supplementary Material. Further inquiries can be directed to the corresponding authors.

## Supplementary Materials

The following supporting information can be downloaded at: https://www.mdpi.com/article/10.3390/biom15060836/s1, Figure S1—Flowchart of the study; Figure S2—Schematic representation of the in-house optimized protocol used for the isolation of EVs from seminal plasma; Table S1—Software and tools used for Transcriptomic analysis; Table S2—Probes assay used for gene expression analysis performed by ddPCR; Table S3—Semen characteristics of groups NORMO, OAT, AZO; Table S4—List of total proteins identified through gel-free approach by LC-MS/MS; Table S5—Process network analysis (MetaCore) of NORMO EVs protein cargo by GO; Table S6—Differential proteomic analysis results.

## Abbreviations

The following abbreviations are used in this manuscript:

EVs	Extracellular Vesicles
NORMO	Normozoospermia
OAT	OligoAsthenoTeratozoospermia
AZO	Azoospermia
NTA	Nanoparticle Tracking Analysis
PCR	Polymerase Chain Reaction
*GAPDH*	*Glyceraldehyde**-3-Phosphate Dehydrogenase*
*RNY4*	*RNA, Ro60-Associated Y4*
*HSPA70*	*Heat shock protein family A (Hsp70) member 4*
*CRISP1*	*Cysteine Rich Secretory Protein 1*
*CRISP2*	*Cysteine Rich Secretory Protein 2*
*CRISP3*	*Cysteine Rich Secretory Protein 3*
*SPP1*	*Secreted Phosphoprotein 1*
*MIF*	*Macrophage Migration Inhibitory Factor*
*SPAM1*	*Sperm Adhesion Molecule 1*
*ADAM2*	*ADAM Metallopeptidase Domain 2*
*PGK2*	*Phosphoglycerate Kinase 2*
*CLGN*	*Calmegin*
*PAEP*	*Progestagen-Associated Endometrial Protein*
*CLIC4*	*Chloride intracellular channel 4*
*KLK3*	*Kallikrein-related peptidase 3*
*IDH1*	*Ocitrate dehydrogenase (NADP(+)) 1, cytosolic*
*PSCA*	*Prostate stem cell antigen*
*PARK7*	*Parkinsonism-associated deglycase*

## References

[1] Candenas L., Chianese R. (2020). Exosome Composition and Seminal Plasma Proteome: A Promising Source of Biomarkers of Male Infertility. Int. J. Mol. Sci..

[2] Moura A.A., Memili E., Portela A.M.R., Viana A.G., Velho A.L.C., Bezerra M.J.B., Vasconselos F.R. (2018). Seminal plasma proteins and metabolites: Effects on sperm function and potential as fertility markers. Anim. Reprod..

[3] Szczykutowicz J., Kałuża A., Kaźmierowska-Niemczuk M., Ferens-Sieczkowska M. (2019). The Potential Role of Seminal Plasma in the Fertilization Outcomes. BioMed Res. Int..

[4] Macanovic B., Vucetic M., Jankovic A., Stancic A., Buzadzic B., Garalejic E., Korac A., Korac B., Otasevic V. (2015). Correlation between Sperm Parameters and Protein Expression of Antioxidative Defense Enzymes in Seminal Plasma: A Pilot Study. Dis. Markers.

[5] Ayaz A., Houle E., Pilsner J.R. (2021). Extracellular vesicle cargo of the male reproductive tract and the paternal preconception environment. Syst. Biol. Reprod. Med..

[6] Tschuschke M., Kocherova I., Bryja A., Mozdziak P., Angelova Volponi A., Janowicz K., Sibiak R., Piotrowska-Kempisty H., Iżycki D., Bukowska D. (2020). Inclusion Biogenesis, Methods of Isolation and Clinical Application of Human Cellular Exosomes. J. Clin. Med..

[7] Jeppesen D.K., Zhang Q., Franklin J.L., Coffey R.J. (2023). Extracellular vesicles and nanoparticles: Emerging complexities. Trends Cell Biol..

[8] Mashouri L., Yousefi H., Aref A.R., Ahadi A.M., Molaei F., Alahari S.K. (2019). Exosomes: Composition, biogenesis, and mechanisms in cancer metastasis and drug resistance. Mol. Cancer.

[9] Tamessar C.T., Trigg N.A., Nixon B., Skerrett-Byrne D.A., Sharkey D.J., Robertson S.A., Bromfield E.G., Schjenken J.E. (2021). Roles of male reproductive tract extracellular vesicles in reproduction. Am. J. Reprod. Immunol..

[10] Dai J., Su Y., Zhong S., Cong L., Liu B., Yang J., Tao Y., He Z., Chen C., Jiang Y. (2020). Exosomes: Key players in cancer and potential therapeutic strategy. Signal Transduct. Target. Ther..

[11] Baskaran S., Panner Selvam M.K., Agarwal A. (2020). Exosomes of male reproduction. Advances in Clinical Chemistry.

[12] Vickram A.S., Srikumar P.S., Srinivasan S., Jeyanthi P., Anbarasu K., Thanigaivel S., Nibedita D., Jenila Rani D., Rohini K. (2021). Seminal exosomes—An important biological marker for various disorders and syndrome in human reproduction. Saudi J. Biol. Sci..

[13] Sullivan R. (2016). Epididymosomes: Role of extracellular microvesicles in sperm maturation. Front Biosci (Sch. Ed).

[14] Kalluri R., LeBleu V.S. (2020). The biology, function, and biomedical applications of exosomes. Science.

[15] Machtinger R., Laurent L.C., Baccarelli A.A. (2016). Extracellular vesicles: Roles in gamete maturation, fertilization and embryo implantation. Hum. Reprod. Update.

[16] Méar L.O., Tsai P.-S., Tamessar C.T., Schjenken J.E., Nixon B. (2024). Epididymosomes: Composition and Functions for Sperm Maturation. Advances in Anatomy, Embryology and Cell Biology.

[17] Saez F., Sullivan R. (2016). Prostasomes, post-testicular sperm maturation and fertility. Front Biosci (Landmark Ed).

[18] WHO Laboratory Manual for the Examination and Processing of Human Semen. https://www.who.int/publications-detail-redirect/9789240030787.

[19] Barceló M., Mata A., Bassas L., Larriba S. (2018). Exosomal microRNAs in seminal plasma are markers of the origin of azoospermia and can predict the presence of sperm in testicular tissue. Hum. Reprod..

[20] Mercadal M., Herrero C., López-Rodrigo O., Castells M., de la Fuente A., Vigués F., Bassas L., Larriba S. (2020). Impact of Extracellular Vesicle Isolation Methods on Downstream miRNA Analysis in Semen: A Comparative Study. Int. J. Mol. Sci..

[21] Fortunato D., Mladenović D., Criscuoli M., Loria F., Veiman K.-L., Zocco D., Koort K., Zarovni N. (2021). Opportunities and Pitfalls of Fluorescent Labeling Methodologies for Extracellular Vesicle Profiling on High-Resolution Single-Particle Platforms. Int. J. Mol. Sci..

[22] Luddi A., Zarovni N., Maltinti E., Governini L., De Leo V., Cappelli V., Quintero L., Paccagnini E., Loria F., Piomboni P. (2019). Clues to Non-Invasive Implantation Window Monitoring: Isolation and Characterisation of Endometrial Exosomes. Cells.

[23] Sinha P., Poland J., Schnölzer M., Rabilloud T. (2001). A new silver staining apparatus and procedure for matrix-assisted laser desorption/ionization-time of flight analysis of proteins after two-dimensional electrophoresis. Proteomics.

[24] Deutsch E.W., Bandeira N., Perez-Riverol Y., Sharma V., Carver J.J., Mendoza L., Kundu D.J., Wang S., Bandla C., Kamatchinathan S. (2023). The ProteomeXchange consortium at 10 years: 2023 update. Nucleic Acids Res..

[25] Perez-Riverol Y., Bai J., Bandla C., García-Seisdedos D., Hewapathirana S., Kamatchinathan S., Kundu D.J., Prakash A., Frericks-Zipper A., Eisenacher M. (2022). The PRIDE database resources in 2022: A hub for mass spectrometry-based proteomics evidences. Nucleic Acids Res..

[26] Ponchia R., Bruno A., Renzi A., Landi C., Shaba E., Luongo F.P., Haxhiu A., Artini P.G., Luddi A., Governini L. (2021). Oxidative Stress Measurement in Frozen/Thawed Human Sperm: The Protective Role of an In Vitro Treatment with Myo-Inositol. Antioxidants.

[27] Wingett S.W., Andrews S. (2018). FastQ Screen: A tool for multi-genome mapping and quality control. F1000Research.

[28] Ewels P., Magnusson M., Lundin S., Käller M. (2016). MultiQC: Summarize analysis results for multiple tools and samples in a single report. Bioinformatics.

[29] Chen S., Zhou Y., Chen Y., Gu J. (2018). fastp: An ultra-fast all-in-one FASTQ preprocessor. Bioinformatics.

[30] Frankish A., Diekhans M., Ferreira A.-M., Johnson R., Jungreis I., Loveland J., Mudge J.M., Sisu C., Wright J., Armstrong J. (2019). GENCODE reference annotation for the human and mouse genomes. Nucleic Acids Res..

[31] Dobin A., Davis C.A., Schlesinger F., Drenkow J., Zaleski C., Jha S., Batut P., Chaisson M., Gingeras T.R. (2013). STAR: Ultrafast universal RNA-seq aligner. Bioinformatics.

[32] Danecek P., Bonfield J.K., Liddle J., Marshall J., Ohan V., Pollard M.O., Whitwham A., Keane T., McCarthy S.A., Davies R.M. (2021). Twelve years of SAMtools and BCFtools. Gigascience.

[33] Smith T., Heger A., Sudbery I. (2017). UMI-tools: Modeling sequencing errors in Unique Molecular Identifiers to improve quantification accuracy. Genome Res..

[34] Okonechnikov K., Conesa A., García-Alcalde F. (2016). Qualimap 2: Advanced multi-sample quality control for high-throughput sequencing data. Bioinformatics.

[35] Liao Y., Smyth G.K., Shi W. (2014). featureCounts: An efficient general purpose program for assigning sequence reads to genomic features. Bioinformatics.

[36] Love M.I., Huber W., Anders S. (2014). Moderated estimation of fold change and dispersion for RNA-seq data with DESeq2. Genome Biol..

[37] Wickham H., Averick M., Bryan J., Chang W., McGowan L.D., François R., Grolemund G., Hayes A., Henry L., Hester J. (2019). Welcome to the Tidyverse. J. Open Source Softw..

[38] Allaire J.J. (2011). *RStudio: Integrated Development Environment for R*. UseR! 2011 Conference. https://www.r-project.org/conferences/useR-2011/abstracts/180111-allairejj.pdf.

[39] Chen VennDiagram: Generate High-Resolution Venn and Euler Plots. *Software: VennDiagram (Generate High-Resolution Venn and Euler Plots) (R-Packages)*. https://cran.r-project.org/web/packages/VennDiagram/index.html.

[40] Schloerke B., Cook D., Larmarange J., Briatte F., Marbach M., Thoen E., Elberg A., Toomet O., Crowley J., Hofmann H. (2024). GGally: Extension to “ggplot2”. Software: GGally (Extension to ‘Ggplot2’) (R-Packages). https://cran.r-project.org/web/packages/GGally/index.html.

[41] Kremer L.P.M., Anders S. Ggpointdensity: A Cross Between a 2D Density Plot and a Scatter Plot. *Software: Ggpointdensity (A Cross Between a 2D Density Plot and a Scatter Plot) (R-Packages)*. https://cran.r-project.org/web/packages/ggpointdensity/index.html.

[42] Slowikowski K., Schep A., Hughes S., Dang T.K., Lukauskas S., Irisson J.-O., Kamvar Z.N., Ryan T., Christophe D., Hiroaki Y. Ggrepel: Automatically Position Non-Overlapping Text Labels with “Ggplot2”. *Software: Ggrepel (Automatically Position Non-Overlapping Text Labels with ‘Ggplot2’) (R-Packages)*. https://cran.r-project.org/web/packages/ggrepel/index.html.

[43] Kolde R. Pheatmap: Pretty Heatmaps. *Software: Pheatmap (Pretty Heatmaps) (R-Rackages)*. https://cran.r-project.org/web/packages/pheatmap/index.html.

[44] Blighe K., Rana S., Lewis M. EnhancedVolcano: Publication-Ready Volcano Plots with Enhanced Colouring and Labeling. https://bioconductor.org/packages/devel/bioc/vignettes/EnhancedVolcano/inst/doc/EnhancedVolcano.html.

[45] Ahlmann-Eltze C. Ggupset: Combination Matrix Axis for “Ggplot2” to Create “UpSet” Plots 2020. *Software: Ggupset (Combination Matrix Axis for ‘Ggplot2’ to Create ‘UpSet’ Plots) (R-Packages)*. https://cran.r-project.org/web/packages/ggupset/index.html.

[46] Jawaid W. (2023). EnrichR: Provides an R Interface to “Enrichr”. *Software: EnrichR (Provides an R Interface to ‘Enrichr’) (R-Packages)*. https://cran.r-project.org/web/packages/enrichR/index.html.

[47] Aleksander S.A., Balhoff J., Carbon S., Cherry J.M., Drabkin H.J., Ebert D., Feuermann M., Gaudet P., Harris N.L., Gene Ontology Consortium (2023). The Gene Ontology knowledgebase in 2023. Genetics.

[48] Kanehisa M., Furumichi M., Sato Y., Kawashima M., Ishiguro-Watanabe M. (2023). KEGG for taxonomy-based analysis of pathways and genomes. Nucleic Acids Res..

[49] Shaba E., Landi C., Carleo A., Vantaggiato L., Paccagnini E., Gentile M., Bianchi L., Lupetti P., Bargagli E., Prasse A. (2021). Proteome Characterization of BALF Extracellular Vesicles in Idiopathic Pulmonary Fibrosis: Unveiling Undercover Molecular Pathways. Int. J. Mol. Sci..

[50] Combe M., Isaac K.S., Plews J.R., Sokolenko S. (2025). Quantifying extracellular vesicle heterogeneity: The effect of process conditions on protein cargo for skin therapy. Stem Cell Res. Ther..

[51] Cannarella R., Barbagallo F., Crafa A., La Vignera S., Condorelli R.A., Calogero A.E. (2020). Seminal Plasma Transcriptome and Proteome: Towards a Molecular Approach in the Diagnosis of Idiopathic Male Infertility. Int. J. Mol. Sci..

[52] Koppers A.J., Reddy T., O’Bryan M.K. (2011). The role of cysteine-rich secretory proteins in male fertility. Asian J. Androl..

[53] Da Ros V.G., Muñoz M.W., Battistone M.A., Brukman N.G., Carvajal G., Curci L., Gómez-ElIas M.D., Cohen D.B.J., Cuasnicu P.S. (2015). From the epididymis to the egg: Participation of CRISP proteins in mammalian fertilization. Asian J. Androl..

[54] Tamessar C.T., Anderson A.L., Bromfield E.G., Trigg N.A., Parameswaran S., Stanger S.J., Weidenhofer J., Zhang H.-M., Robertson S.A., Sharkey D.J. (2024). The efficacy and functional consequences of interactions between human spermatozoa and seminal fluid extracellular vesicles. Reprod. Fertil..

[55] Mateo-Otero Y., Yeste M., Roca J., Llavanera M., Bucci D., Galeati G., Spinaci M., Barranco I. (2022). Seminal extracellular vesicles subsets modulate gene expression in cumulus cells of porcine in vitro matured oocytes. Sci. Rep..

[56] An C.-N., Jiang H., Wang Q., Yuan R.-P., Liu J.-M., Shi W.-L., Zhang Z.-Y., Pu X.-P. (2011). Down-regulation of DJ-1 protein in the ejaculated spermatozoa from Chinese asthenozoospermia patients. Fertil. Steril..

[57] Recuero S., Delgado-Bermúdez A., Mateo-Otero Y., Garcia-Bonavila E., Llavanera M., Yeste M. (2021). Parkinson Disease Protein 7 (PARK7) Is Related to the Ability of Mammalian Sperm to Undergo In Vitro Capacitation. Int. J. Mol. Sci..

[58] Myers K., Somanath P.R., Berryman M., Vijayaraghavan S. (2004). Identification of chloride intracellular channel proteins in spermatozoa. FEBS Lett..

