# Supplementary material for: Unraveling the Multi-Omic Landscape of Extracellular Vesicles in Human Seminal Plasma
Source: Biomolecules. 2025 Jun 7;15(6):836. doi: 10.3390/biom15060836 (PMC12190863; doi:10.3390/biom15060836)
Supplement: Supplementary file 1 [file biomolecules-15-00836-s001.zip › biomolecules-3612311_Supplementary Figure S1-S2.pdf]

SUPPLEMENTARY FIGURES

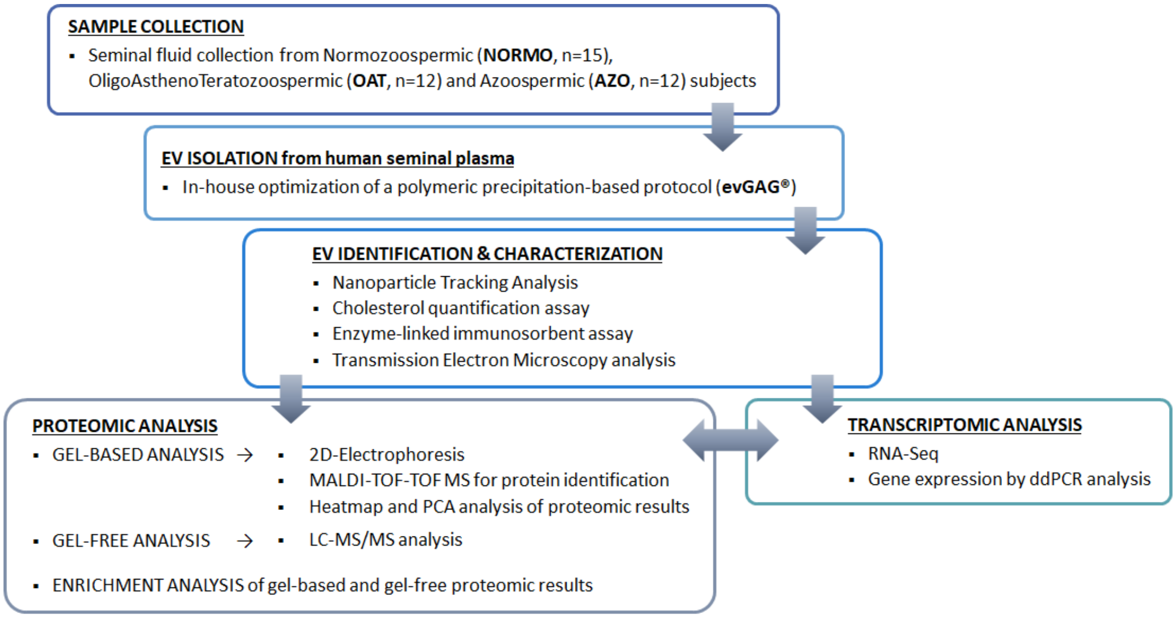

Figure S1 – Flowchart of the study

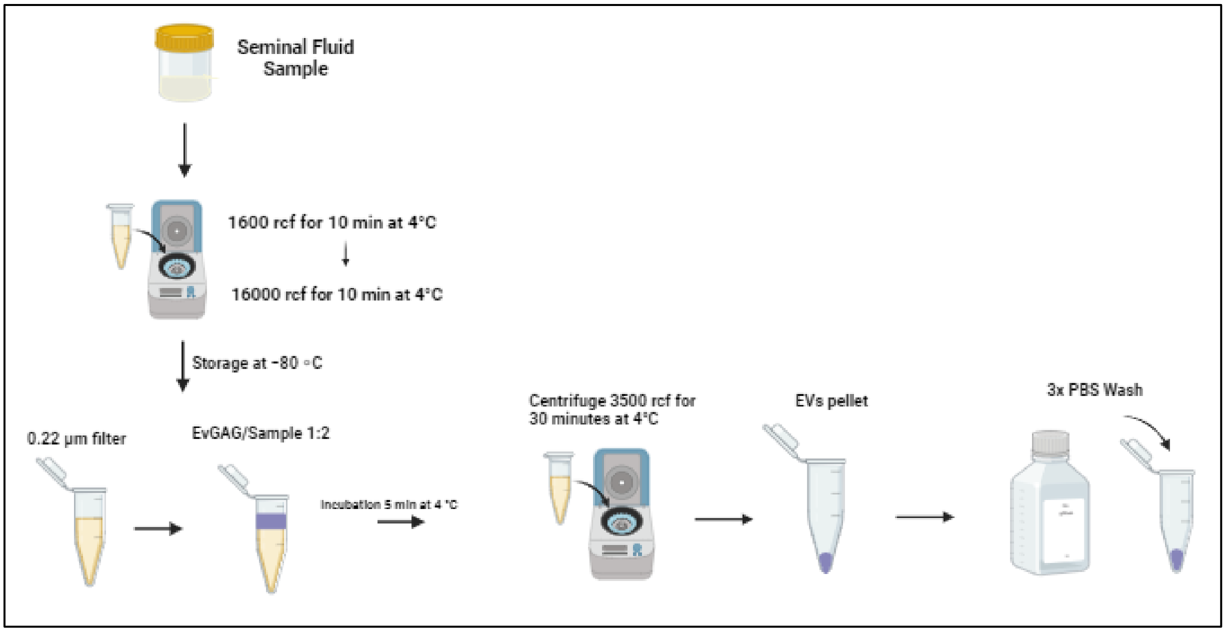

Figure S2 - Schematic representation of the in-house optimized protocol used for the isolation of EVs from seminal plasma
