# Supplementary material for: Unraveling the Multi-Omic Landscape of Extracellular Vesicles in Human Seminal Plasma
Source: Biomolecules. 2025 Jun 7;15(6):836. doi: 10.3390/biom15060836 (PMC12190863; doi:10.3390/biom15060836)
Supplement: Supplementary file 1 [file biomolecules-15-00836-s001.zip › biomolecules-3612311_Supplementary Table S6.pdf]

Supplemetary Table S6

Table S6 - Differential proteomic analysis results. Table reports the spot number, UniProt protein name, abbreviation, and accession number (AC). In the table are also reported statistical results by ANOVA analysis (p<0.05). NORMO, OAT and AZO %V means values are reported, together with the fold-change of the highly abundant proteins in NORMO vs OAT and AZO, highly abundant in OAT vs NORMO and AZO and highly abundant in AZO vs OAT. In the right part of the table are reported the identification data such as Mascot Search Results with the score, matched peptides, coverage (%) and peptide sequence in case of tandem mass spectrometry for protein identification confirmation.

Spot nr	Protein name	Entry name *HUMAN*	AC	Anova test				Ratio					p/MW	Mascot Search Results		
								UP IN NORMO		UP IN OAT		UP IN AZO				
				Anova (p)	NORMO	OAT	AZO	N/OAT	N/AZO	OAT/N	OAT/AZO	AZO/OAT		Score	Matched peptides	Coverage (%)
1	**Methanethiol oxidase**	SBP1	Q13228	5,05E-03	0,0165	0,0065	0,0117	2,53	1,41	0,39	0,56	1,80	5,93 - 2928	245	17/23	46%
2	**Heat shock protein HSP 90-beta**	HS90B	P08238	1,02E-04	0,0183	0,0027	0,0059	6,70	3,12	0,15	0,47	2,15	4,97 - 3554	135	12/15	19%
7	**Rab GDP dissociation inhibitor beta**	GDIB	P50395	1,66E-03	0,0501	0,0206	0,0315	2,43	1,59	0,41	0,65	1,53	6,11 - 1087	230	18/19	39%
10	**Protein disulfide-isomerase A3**	PDIA3	P30101	6,40E-03	0,0286	0,0210	0,0117	1,37	2,45	0,73	1,79	0,56	5,98 - 7146	224	19/23	37%
12	**Prostatic acid phosphatase**	PPAP	P15309	7,44E-04	0,0337	0,0073	0,0108	4,62	3,12	0,22	0,68	1,48	5,83 - 4880	148	10/14	29%
13	**Parkinson disease protein 7**	PARK7	Q99497	1,57E-02	0,0230	0,0096	0,0179	2,41	1,28	0,42	0,53	1,88	6,33 - 0050	90	7/10	39%
14	**Plastin-2**	PLSL	P13796	5,68E-03	0,1350	0,0473	0,0761	2,86	1,77	0,35	0,62	1,61	5,29 - 0814	381	27/30	47%
17	**Purine nucleoside phosphorylase**	PNPH	P00491	2,04E-02	0,0341	0,0088	0,0309	3,86	1,10	0,26	0,29	3,50	6,45 - 2325	216	14/15	51%
21	**Chloride intracellular channel protein 4**	CLIC4	Q9Y696	3,63E-03	0,0291	0,0093	0,0114	3,14	2,56	0,32	0,82	1,22	5,45 - 8982	95	7/10	31%
23	**Chloride intracellular channel protein 4**	CLIC4	Q9Y696	5,15E-03	0,0516	0,0208	0,023	2,49	2,24	0,40	0,90	1,11	5,45 - 8982	137	114	9/18 41% KPADLQNLAPGTHPPFITFNSEVK
	**Putative hydroxypyruvate isomerase**	HYI	Q5T013										5,36 - 0501		74	6/18 28%
27	**Heat shock 70 kDa protein 1A / Heat shock 70 kDa protein 1B**	HS71A / HS71B	P0DMV8 / P0DMV9	1,11E-02	0,0237	0,0126	0,0073	1,89	3,23	0,53	1,71	0,58	5,48 - 70294	107	8/11	17%
32	**RuvB-like 1**	RUVB1	Q9Y265	2,42E-02	0,0182	0,0081	0,0142	2,25	1,28	0,44	0,57	1,76	6,02 - 50538	123	77	7/18 26% ALESSIAPIVIFASNR
	**Retinal dehydrogenase 1**	AL1A1	P00352										6,30 - 55454		61	7/18 13% TIPIDGNFFTYTR
34	**4-trimethylaminobutyraldehyde dehydrogenase**	AL9A1	P49189	2,07E-02	0,0990	0,0485	0,0533	2,04	1,86	0,49	0,91	1,10	5,69 - 54679	156	12/14	21%
35	**Semenogelin-1**	SEMG1	P04279	9,57E-03	0,0366	0,0103	0,013	3,56	2,81	0,28	0,79	1,27	9,30 - 52157	138	10/16	32%
36	**Alpha-enolase**	ENOA	P06733	2,59E-02	0,0905	0,0368	0,0825	2,46	1,10	0,41	0,45	2,24	7,01 - 47481	425	30/33	68%
37	**Heat shock 70 kDa protein 1A / Heat shock 70 kDa protein 1B**	HS71A / HS71B	P0DMV8 / P0DMV9	4,19E-02	0,1293	0,0643	0,156	2,01	0,83	0,50	0,41	2,43	5,48 - 70294	97	9/19	21%
40	**Beta-hexosaminidase subunit alpha (C-term fragment)**	HEXA	P06865	4,81E-02	0,1045	0,0449	0,0541	2,33	1,93	0,43	0,83	1,21	5,04 - 61120	63	5/9	11%
41	**Plastin-2**	PLSL	P13796	6,17E-02	0,1330	0,0647	0,0686	2,05	1,94	0,49	0,94	1,06	5,29 - 70814	157	17/40	31%
43	**Prostatic acid phosphatase**	PPAP	P15309	4,04E-02	0,0413	0,0167	0,03	2,47	1,38	0,40	0,56	1,80	5,83 - 4880	184	12/16	37%
44	**Ubiquitin-40S ribosomal protein S27a (N-term fragment)**	RS27A	P62979	2,32E-02	0,0253	0,0387	0,0204	0,65	1,24	1,53	1,90	0,53	9,68 - 8296	44	2/2	18% IQDKEGIPPDQQR
48	**Rab GDP dissociation inhibitor beta**	GDIB	P50395	1,04E-01	0,0703	0,0337	0,0509	2,09	1,38	0,48	0,66	1,51	6,11 - 1087	369	26/29	57%
50	**Prostatic acid phosphatase**	PPAP	P15309	9,17E-02	0,0801	0,0493	0,0428	1,62	1,87	0,62	1,15	0,87	5,83 - 44880	146	11/16	33%

**52**	**Clusterin**	CLUS	P10909	2,89E-02	0,5542	0,6979	0,1992	0,79	2,78	1,26	3,50	0,29	5,89 - 53031	217	16/21	38%
**54**	**Serum amyloid P-component**	SAMP	P02743	1,14E-02	0,0477	0,0134	0,0064	3,57	7,43	0,28	2,08	0,48	6,10 - 25485	108	7/13	30%
**55**	**Isocitrate dehydrogenase [NADP] cytoplasmic**	IDHC	O75874	1,95E-02	0,0446	0,0139	0,0631	3,20	0,71	0,31	0,22	4,53	6,53 - 46915	302	25/42	68%
**61**	**Albumin**	ALBU	P02768	9,42E-02	0,0721	0,0360	0,0312	2,00	2,31	0,50	1,15	0,87	5,92 - 71317	146	13/20	20%
**64**	**Acyl-CoA-binding protein**	ACBP	P07108	6,25E-02	0,1345	0,0494	0,1064	2,73	1,26	0,37	0,46	2,16	6,12 - 10038	77	6/8	62%
															TKPSDEEMLFIYGHYK	
**67**	**Serum amyloid P-component**	SAMP	P02743	6,31E-02	0,0411	0,0130	0,0204	3,17	2,01	0,32	0,64	1,57	6,10 - 25371	79	4/4	19%
**68**	**Heat shock protein HSP 90-alpha**	HS90A	P07900	5,90E-02	0,0932	0,0247	0,0565	3,78	1,65	0,26	0,44	2,29	4,94 - 85006	163	15/18	18%
**69**	**Ras-related protein Rab-14**	RAB14	P61106	1,54E-01	0,0323	0,0356	0,0164	0,91	1,97	1,10	2,17	0,46	5,85 - 24110	88	5/8	33%
**70**	**NADH dehydrogenase [ubiquinone] 1 alpha subcomplex subunit 4-like**	NUA4L	Q9NRX3	4,73E-02	0,1116	0,0257	0,0297	4,35	3,76	0,23	0,86	1,16	9,94 - 10073	42	3/3	22%
															MAGASLGARFYR	
**71**	**Semenogelin-1 C-term fragment**	SEMG1	P04279	4,85E-02	0,2590	0,4892	0,1563	0,53	1,66	1,89	3,13	0,32	9,30 - 52157	115	8/11	16%
**72**	**Isocitrate dehydrogenase [NADP] cytoplasmic**	IDHC	O75874	4,57E-02	0,0392	0,0096	0,0449	4,10	0,87	0,24	0,21	4,69	6,53 - 46915	335	24/27	54%
**74**	**Nucleoside diphosphate kinase A**	NDKA	P15531	1,23E-01	0,1916	0,1565	0,065	1,22	2,95	0,82	2,41	0,42	5,83 - 17309	76	5/6	31%
**76**	**Prostatic acid phosphatase**	PPAP	P15309	2,40E-01	0,0316	0,0165	0,0267	1,91	1,18	0,52	0,62	1,61	5,83 - 44880	172	125	12/37 34%
	**Fibronectin C-term fragment**	FINC	P02751										5,32 - 275742		81	16/37 7%
	**26S proteasome regulatory subunit 7**	PRS7	P35998										5,71 - 49002		48	7/37 16%
**79**	**Protein disulfide-isomerase A3**	PDIA3	P30101	1,87E-01	0,0068	0,0055	0,0025	1,23	2,74	0,81	2,23	0,45	5,98 - 57146	128	10/12	18%
**81**	**Prolactin-inducible protein**	PIP	P12273	2,39E-01	0,1814	0,2210	0,1144	0,82	1,59	1,22	1,93	0,52	8,26 - 16847	143	8/14	48%
**83**	**Prolactin-inducible protein**	PIP	P12273	7,50E-02	0,0500	0,1581	0,0664	0,32	0,75	3,16	2,38	0,42	8,26 - 16847	134	8/16	62%
**84**	**Parkinson disease protein 7**	PARK7	Q99497	1,78E-01	0,0752	0,0314	0,0868	2,40	0,87	0,42	0,36	2,76	6,33 - 20050	240	17/20	79%
**85**	**Clusterin**	CLUS	P10909	1,12E-01	0,4892	0,6414	0,1577	0,76	3,10	1,31	4,07	0,25	5,89 - 53031	204	16/21	31%
**86**	**Tripeptidyl-peptidase 1**	TPP1	O14773	7,32E-02	0,0429	0,0194	0,0045	2,21	9,56	0,45	4,32	0,23	6,01 - 61723	93	7/11	20%
**87**	**Semenogelin-1 C-term fragment**	SEMG1	P04279	2,23E-01	0,2629	0,3048	0,1555	0,86	1,69	1,16	1,96	0,51	9,30 - 52157	93	7/12	13%
															HQHGSHGGLDIVIIEQEDDSDR	
**88**	**Isocitrate dehydrogenase [NADP] cytoplasmic**	IDHC	O75874	5,33E-03	0,0448	0,0142	0,0719	3,15	0,62	0,32	0,20	5,05	6,53 - 46915	335	24/27	54%
