# Supplementary material for: Unraveling the Multi-Omic Landscape of Extracellular Vesicles in Human Seminal Plasma
Source: Biomolecules. 2025 Jun 7;15(6):836. doi: 10.3390/biom15060836 (PMC12190863; doi:10.3390/biom15060836)
Supplement: Supplementary file 1 [file biomolecules-15-00836-s001.zip › biomolecules-3612311_Supplementary Tables S1new.pdf]

SUPPLEMENTARY TABLES

Table S1 - Software and tools used for Transcriptomic analysis

Software and tools	***Ref.***
FASTQC	27
MultiQC	28
fastp	29
GENCODE	30
STAR	31
SAMtools	32
UMI-tools	33
QualiMap2	34
featureCounts	35
DESeq2	36
tidyverse	37
R & RStudio	38
VennDiagram	39
GGally	40
ggpointdensity	41
ggrepel	42
pheatmap	43
EnhancedVolcano	44
ggupset	45
Enrichr	46
Gene Ontology	47
KEGG	48
