# Supplementary material for: Unraveling the Multi-Omic Landscape of Extracellular Vesicles in Human Seminal Plasma
Source: Biomolecules. 2025 Jun 7;15(6):836. doi: 10.3390/biom15060836 (PMC12190863; doi:10.3390/biom15060836)
Supplement: Supplementary file 1 [file biomolecules-15-00836-s001.zip › biomolecules-3612311_Supplementary Tables S2-S3-S4-S5.pdf]

SUPPLEMENTARY TABLES

Table S2 – Probes assay used for gene expression analysis performed by ddPCR

	Name	*Acron.*	*Channel*	*Assay ID*
Reference Genes	*Glyceraldehyde-3-Phosphate Dehydrogenase*	***GAPDH***	Hex	dHsaCPE5031597
	RNA, Ro60-Associated Y4	***RNY4***	Fam	dHsaCNS187520158
	Heat shock protein family A (Hsp70) member 4	***HSPA70***	Hex	dHsaCPE5056147
Target Genes	*Cysteine Rich Secretory Protein 1*	***CRISP1***	Fam	dHsaCPE5053698
	Cysteine Rich Secretory Protein 2	***CRISP2***	Hex	dHsaCPE5053565
	Cysteine Rich Secretory Protein 3	***CRISP3***	Fam	dHsaCPE5053616
	Secreted Phosphoprotein 1	***SPP1***	Hex	dHsaCPE5045617
	Macrophage Migration Inhibitory Factor	***MIF***	Hex	dHsaCPE5191454
	Sperm Adhesion Molecule 1	***SPAM1***	Fam	dHsaCPE5055574
	ADAM Metallopeptidase Domain 2	***ADAM2***	Hex	dHsaCPE5034017
	Phosphoglycerate Kinase 2	***PGK2***	Fam	dHsaCPE5028836
	Calmegin	***CLGN***	Hex	dHsaCPE5045721
	Progestagen Associated Endometrial Protein	***PAEP***	Fam	dHsaCPE5026616
	Chloride intracellular channel 4	***CLIC4***	Hex	dHsaCPE5026589
	Kallikrein related peptidase 3	***KLK3***	Fam	dHsaCPE5026548
	Ocitrate dehydrogenase (NADP(+)) 1, cytosolic	***IDH1***	Hex	dHsaCPE5037211
	Prostate stem cell antigen	***PSCA***	Fam	dHsaCPE5040139
	Parkinsonism associated deglycase	***PARK7***	Fam	dHsaCPE5027748

Table S3– Semen characteristics of groups NORMO, OAT, AZO:

	Reference value (WHO, 2021) 5th centile	Normozoospermic (**NORMO**, n=15)	OligoAstheno Teratozoospermic (**OAT**, n=12)	Azoospermic (**AZO**, n=12)
Semen Volume *(ml)*	*1.4*	3.3 ± 1.0	2.9 ± 0.8	2.0 ± 0.7 *
Sperm Concentration *($x10^6$ sperm/ml)*	*16*	76.4 ± 21.7	5.8 ± 2.9 ***	*Total absence of sperm*
Sperm Total Motility *(%)*	*42*	58.3 ± 8.0	34.7 ± 4.5 ***	
Sperm Vitality *(%)*	*54*	80.3 ± 6.9	68.6 ± 8.8 **	
Sperm Morphology *(%)*	*4*	14.3 ± 3.4	2.4 ± 0.8 ***	

* $p< 0.05$; ** $p<0.01$; *** $p<0.001$

Table S4 – List of total proteins (n=1133) identified through gel-free approach by LC-MS/MS

Protein FDR Confidence	Accession	Description	Coverage [%]	# Peptides	# Unique Peptides	# Razor Peptides	# AAs	MW [kDa]	calc. pI
High	P02788	Lactotransferrin OS=Homo sapiens OX=9606 GN=LTF PE=1 SV=6	71	76	76	0	710	78.1	8.12
High	Q02383	Semenogelin-2 OS=Homo sapiens OX=9606 GN=SEMG2 PE=1 SV=1	80	59	54	0	582	65.4	9.07
High	P04279	Semenogelin-1 OS=Homo sapiens OX=9606 GN=SEMG1 PE=1 SV=2	85	60	55	5	462	52.1	9.29
High	P02751	Fibronectin OS=Homo sapiens OX=9606 GN=FN1 PE=1 SV=5	39	73	73	0	2477	272.2	5.5
High	P63261	Actin, cytoplasmic 2 OS=Homo sapiens OX=9606 GN=ACTG1 PE=1 SV=1	73	31	1	30	375	41.8	5.48
High	P60709	Actin, cytoplasmic 1 OS=Homo sapiens OX=9606 GN=ACTB PE=1 SV=1	73	31	1	30	375	41.7	5.48
High	P02768	Albumin OS=Homo sapiens OX=9606 GN=ALB PE=1 SV=2	72	50	50	0	609	69.3	6.28
High	P13796	Plastin-2 OS=Homo sapiens OX=9606 GN=LCP1 PE=1 SV=6	66	42	35	7	627	70.2	5.43
High	P07900	Heat shock protein HSP 90-alpha OS=Homo sapiens OX=9606 GN=HSP90AA1 PE=1 SV=5	47	44	30	14	732	84.6	5.02
High	P49327	Fatty acid synthase OS=Homo sapiens OX=9606 GN=FASN PE=1 SV=3	30	61	61	0	2511	273.3	6.44
High	E7ER45	Maltase-glucoamylase, intestinal OS=Homo sapiens OX=9606 GN=MGAM PE=1 SV=2	23	42	42	0	2753	311.8	5.41
High	O15230	Laminin subunit alpha-5 OS=Homo sapiens OX=9606 GN=LAMA5 PE=1 SV=8	20	62	62	0	3695	399.5	7.02
High	P12277	Creatine kinase B-type OS=Homo sapiens OX=9606 GN=CKB PE=1 SV=1	78	25	25	0	381	42.6	5.59
High	P08238	Heat shock protein HSP 90-beta OS=Homo sapiens OX=9606 GN=HSP90AB1 PE=1 SV=4	44	40	24	2	724	83.2	5.03
High	P55268	Laminin subunit beta-2 OS=Homo sapiens OX=9606 GN=LAMB2 PE=1 SV=2	32	48	48	0	1798	195.9	6.52
High	P10909	Clusterin OS=Homo sapiens OX=9606 GN=CLU PE=1 SV=1	50	30	30	0	449	52.5	6.27
High	P15309	Prostatic acid phosphatase OS=Homo sapiens OX=9606 GN=ACP3 PE=1 SV=3	33	21	21	0	386	44.5	6.24
High	P35579	Myosin-9 OS=Homo sapiens OX=9606 GN=MYH9 PE=1 SV=4	26	46	35	11	1960	226.4	5.6
High	P15311	Ezrin OS=Homo sapiens OX=9606 GN=EZR PE=1 SV=4	49	39	27	12	586	69.4	6.27
High	P54652	Heat shock-related 70 kDa protein 2 OS=Homo sapiens OX=9606 GN=HSPA2 PE=1 SV=1	51	34	19	15	639	70	5.74
High	P13797	Plastin-3 OS=Homo sapiens OX=9606 GN=PLS3 PE=1 SV=4	48	32	25	0	630	70.8	5.6
High	P14618	Pyruvate kinase PKM OS=Homo sapiens OX=9606 GN=PKM PE=1 SV=4	63	32	14	18	531	57.9	7.84
High	P11142	Heat shock cognate 71 kDa protein OS=Homo sapiens OX=9606 GN=HSPA8 PE=1 SV=1	47	31	17	1	646	70.9	5.52
High	A0A0G2JIW1	Heat shock 70 kDa protein 1B OS=Homo sapiens OX=9606 GN=HSPA1B PE=1 SV=1	39	29	17	10	642	70.1	5.66
High	Q9NR99	Matrix-remodeling-associated protein 5 OS=Homo sapiens OX=9606 GN=MXRA5 PE=1 SV=3	19	46	46	0	2828	312	8.32
High	P11021	Endoplasmic reticulum chaperone BiP OS=Homo sapiens OX=9606 GN=HSPA5 PE=1 SV=2	51	33	31	0	654	72.3	5.16
High	P80303	Nucleobindin-2 OS=Homo sapiens OX=9606 GN=NUCB2 PE=1 SV=3	42	22	21	1	420	50.2	5.12
High	P68133	Actin, alpha skeletal muscle OS=Homo sapiens OX=9606 GN=ACTA1 PE=1 SV=1	46	24	5	0	377	42	5.39
High	P14625	Endoplasmin OS=Homo sapiens OX=9606 GN=HSP90B1 PE=1 SV=1	35	37	35	0	803	92.4	4.84
High	P13639	Elongation factor 2 OS=Homo sapiens OX=9606 GN=EEF2 PE=1 SV=4	43	35	35	0	858	95.3	6.83
High	P78371	T-complex protein 1 subunit beta OS=Homo sapiens OX=9606 GN=CCT2 PE=1 SV=4	60	29	29	0	535	57.5	6.46
High	Q66K79	Carboxypeptidase Z OS=Homo sapiens OX=9606 GN=CPZ PE=1 SV=2	40	23	23	0	652	73.6	7.97
High	A0A494C0G5	Agrin OS=Homo sapiens OX=9606 GN=AGRN PE=1 SV=1	24	37	3	34	1940	203	6.55
High	O00468	Agrin OS=Homo sapiens OX=9606 GN=AGRN PE=1 SV=6	22	36	2	0	2068	217.2	6.39
High	P68371	Tubulin beta-4B chain OS=Homo sapiens OX=9606 GN=TUBB4B PE=1 SV=1	54	21	5	16	445	49.8	4.89
High	P11047	Laminin subunit gamma-1 OS=Homo sapiens OX=9606 GN=LAMC1 PE=1 SV=3	22	36	36	0	1609	177.5	5.12
High	P12273	Prolactin-inducible protein OS=Homo sapiens OX=9606 GN=PIP PE=1 SV=1	77	12	12	0	146	16.6	8.05
High	Q00796	Sorbitol dehydrogenase OS=Homo sapiens OX=9606 GN=SORD PE=1 SV=4	60	19	19	0	357	38.3	7.97
High	P04406	Glyceraldehyde-3-phosphate dehydrogenase OS=Homo sapiens OX=9606 GN=GAPDH PE=1 SV=3	56	17	16	1	335	36	8.46
High	P34931	Heat shock 70 kDa protein 1-like OS=Homo sapiens OX=9606 GN=HSPA1L PE=1 SV=2	38	22	8	0	641	70.3	6.02
High	P11216	Glycogen phosphorylase, brain form OS=Homo sapiens OX=9606 GN=PYGB PE=1 SV=5	28	27	19	7	843	96.6	6.86
High	P30101	Protein disulfide-isomerase A3 OS=Homo sapiens OX=9606 GN=PDIA3 PE=1 SV=4	45	24	18	5	505	56.7	6.35
High	P55072	Transitional endoplasmic reticulum ATPase OS=Homo sapiens OX=9606 GN=VCP PE=1 SV=4	44	34	34	0	806	89.3	5.26
High	Q5JP53	Tubulin beta chain OS=Homo sapiens OX=9606 GN=TUBB PE=1 SV=1	42	18	2	0	426	47.7	4.81
High	P68104	Elongation factor 1-alpha 1 OS=Homo sapiens OX=9606 GN=EEF1A1 PE=1 SV=1	39	18	18	0	462	50.1	9.01
High	P00558	Phosphoglycerate kinase 1 OS=Homo sapiens OX=9606 GN=PGK1 PE=1 SV=3	50	23	20	3	417	44.6	8.1
High	P06727	Apolipoprotein A-IV OS=Homo sapiens OX=9606 GN=APOA4 PE=1 SV=4	53	23	23	0	396	45.3	5.38
High	Q02809	Procollagen-lysine,2-oxoglutarate 5-dioxygenase 1 OS=Homo sapiens OX=9606 GN=PLOD1 PE=1 SV=2	42	27	27	0	727	83.5	6.95
High	P15144	Aminopeptidase N OS=Homo sapiens OX=9606 GN=ANPEP PE=1 SV=4	28	27	27	0	967	109.5	5.48
High	O75369	Filamin-B OS=Homo sapiens OX=9606 GN=FLNB PE=1 SV=2	17	35	35	0	2602	278	5.73
High	P05164	Myeloperoxidase OS=Homo sapiens OX=9606 GN=MPO PE=1 SV=1	30	19	19	0	745	83.8	8.97
High	Q8WUM4	Programmed cell death 6-interacting protein OS=Homo sapiens OX=9606 GN=PDCD6IP PE=1 SV=1	39	32	32	0	868	96	6.52
High	Q14697	Neutral alpha-glucosidase AB OS=Homo sapiens OX=9606 GN=GANAB PE=1 SV=3	30	28	28	0	944	106.8	6.14
High	Q96KP4	Cytosolic non-specific dipeptidase OS=Homo sapiens OX=9606 GN=CNDP2 PE=1 SV=2	52	22	22	0	475	52.8	5.97
High	A0A087WVQ6	Clathrin heavy chain OS=Homo sapiens OX=9606 GN=CLTC PE=1 SV=1	16	25	25	0	1679	191.9	5.69
High	P22314	Ubiquitin-like modifier-activating enzyme 1 OS=Homo sapiens OX=9606 GN=UBA1 PE=1 SV=3	23	21	21	0	1058	117.8	5.76
High	H7C144	Alpha-actinin-4 OS=Homo sapiens OX=9606 GN=ACTN4 PE=1 SV=2	29	24	15	9	906	104.3	5.5
High	O75874	Isocitrate dehydrogenase [NADP] cytoplasmic OS=Homo sapiens OX=9606 GN=IDH1 PE=1 SV=2	48	19	18	1	414	46.6	7.01
High	P46940	Ras GTPase-activating-like protein IQGAP1 OS=Homo sapiens OX=9606 GN=IQGAP1 PE=1 SV=1	18	25	25	0	1657	189.1	6.48
High	P50395	Rab GDP dissociation inhibitor beta OS=Homo sapiens OX=9606 GN=GDI2 PE=1 SV=2	51	21	14	7	445	50.6	6.47
High	P49368	T-complex protein 1 subunit gamma OS=Homo sapiens OX=9606 GN=CCT3 PE=1 SV=4	48	25	25	0	545	60.5	6.49
High	P53396	ATP-citrate synthase OS=Homo sapiens OX=9606 GN=ACLY PE=1 SV=3	26	26	26	0	1101	120.8	7.33
High	H3BR70	Pyruvate kinase OS=Homo sapiens OX=9606 GN=PKM PE=1 SV=1	45	19	1	0	366	40.2	8.03
High	P50990	T-complex protein 1 subunit theta OS=Homo sapiens OX=9606 GN=CCT8 PE=1 SV=4	42	21	21	0	548	59.6	5.6
High	P62805	Histone H4 OS=Homo sapiens OX=9606 GN=H4C1 PE=1 SV=2	59	11	11	0	103	11.4	11.36
High	P98160	Basement membrane-specific heparan sulfate proteoglycan core protein OS=Homo sapiens OX=9606 GN=HSPG2 PE=1 SV=4	8	28	28	0	4391	468.5	6.51
High	P19367	Hexokinase-1 OS=Homo sapiens OX=9606 GN=HK1 PE=1 SV=3	30	27	27	0	917	102.4	6.8
High	P08473	Neprilysin OS=Homo sapiens OX=9606 GN=MME PE=1 SV=2	33	24	24	0	750	85.5	5.73
High	Q71U36	Tubulin alpha-1A chain OS=Homo sapiens OX=9606 GN=TUBA1A PE=1 SV=1	38	15	2	13	451	50.1	5.06
High	P04264	Keratin, type II cytoskeletal 1 OS=Homo sapiens OX=9606 GN=KRT1 PE=1 SV=6	31	21	18	3	644	66	8.12
High	P07205	Phosphoglycerate kinase 2 OS=Homo sapiens OX=9606 GN=PGK2 PE=1 SV=3	46	20	17	0	417	44.8	8.54

High	P49221	Protein-glutamine gamma-glutamyltransferase 4 OS=Homo sapiens OX=9606 GN=TGM4 PE=1 SV=2	36	22	21	1	684	77.1	6.76
High	P0DPH8	Tubulin alpha-3D chain OS=Homo sapiens OX=9606 GN=TUBA3D PE=1 SV=1	38	15	3	0	450	49.9	5.1
High	P02787	Serotransferrin OS=Homo sapiens OX=9606 GN=TF PE=1 SV=3	34	20	20	0	698	77	7.12
High	P00352	Retinal dehydrogenase 1 OS=Homo sapiens OX=9606 GN=ALDH1A1 PE=1 SV=2	34	18	18	0	501	54.8	6.73
High	P68366	Tubulin alpha-4A chain OS=Homo sapiens OX=9606 GN=TUBA4A PE=1 SV=1	33	14	4	0	448	49.9	5.06
High	P48643	T-complex protein 1 subunit epsilon OS=Homo sapiens OX=9606 GN=CCT5 PE=1 SV=1	39	20	19	1	541	59.6	5.66
High	Q03167	Transforming growth factor beta receptor type 3 OS=Homo sapiens OX=9606 GN=TGFBR3 PE=1 SV=3	23	17	17	0	851	93.4	5.71
High	P12821	Angiotensin-converting enzyme OS=Homo sapiens OX=9606 GN=ACE PE=1 SV=1	19	20	20	0	1306	149.6	6.39
High	P39060	Collagen alpha-1(XVIII) chain OS=Homo sapiens OX=9606 GN=COL18A1 PE=1 SV=5	10	16	16	0	1754	178.1	6.01
High	P04083	Annexin A1 OS=Homo sapiens OX=9606 GN=ANXA1 PE=1 SV=2	55	16	16	0	346	38.7	7.02
High	P07237	Protein disulfide-isomerase OS=Homo sapiens OX=9606 GN=P4HB PE=1 SV=3	28	15	15	0	508	57.1	4.87
High	H3BPK7	Alanine--tRNA ligase OS=Homo sapiens OX=9606 GN=AARS1 PE=1 SV=4	17	17	17	0	999	110.1	5.67
High	P08758	Annexin A5 OS=Homo sapiens OX=9606 GN=ANXA5 PE=1 SV=2	39	13	13	0	320	35.9	5.05
High	Q14204	Cytoplasmic dynein 1 heavy chain 1 OS=Homo sapiens OX=9606 GN=DYNC1H1 PE=1 SV=5	5	24	24	0	4646	532.1	6.4
High	M0R1F0	Prostate-specific antigen (Fragment) OS=Homo sapiens OX=9606 GN=KLK3 PE=1 SV=1	42	8	7	1	262	28.6	7.44
High	Q9Y230	RuvB-like 2 OS=Homo sapiens OX=9606 GN=RUVBL2 PE=1 SV=3	45	18	18	0	463	51.1	5.64
High	P06858	Lipoprotein lipase OS=Homo sapiens OX=9606 GN=LPL PE=1 SV=1	29	16	16	0	475	53.1	8.15
High	P35908	Keratin, type II cytoskeletal 2 epidermal OS=Homo sapiens OX=9606 GN=KRT2 PE=1 SV=2	26	18	12	4	639	65.4	8
High	Q14393	Growth arrest-specific protein 6 OS=Homo sapiens OX=9606 GN=GAS6 PE=1 SV=3	24	15	15	0	678	74.9	5.69
High	P06733	Alpha-enolase OS=Homo sapiens OX=9606 GN=ENO1 PE=1 SV=2	36	14	14	0	434	47.1	7.39
High	Q86VP6	Cullin-associated NEDD8-dissociated protein 1 OS=Homo sapiens OX=9606 GN=CAND1 PE=1 SV=2	20	21	21	0	1230	136.3	5.78
High	Q99832	T-complex protein 1 subunit eta OS=Homo sapiens OX=9606 GN=CCT7 PE=1 SV=2	34	18	18	0	543	59.3	7.65
High	F5H2J1	Myosin-IIIb (Fragment) OS=Homo sapiens OX=9606 GN=MYO3B PE=1 SV=1	17	17	16	1	1105	126	7.68
High	P12814	Alpha-actinin-1 OS=Homo sapiens OX=9606 GN=ACTN1 PE=1 SV=2	19	15	7	0	892	103	5.41
High	Q04609	Glutamate carboxypeptidase 2 OS=Homo sapiens OX=9606 GN=FOLH1 PE=1 SV=1	25	16	16	0	750	84.3	6.98
High	P07686	Beta-hexosaminidase subunit beta OS=Homo sapiens OX=9606 GN=HEXB PE=1 SV=3	31	16	15	0	556	63.1	6.76
High	P07355	Annexin A2 OS=Homo sapiens OX=9606 GN=ANXA2 PE=1 SV=2	46	16	16	0	339	38.6	7.75
High	P06865	Beta-hexosaminidase subunit alpha OS=Homo sapiens OX=9606 GN=HEXA PE=1 SV=2	26	16	15	1	529	60.7	5.16
High	P50991	T-complex protein 1 subunit delta OS=Homo sapiens OX=9606 GN=CCT4 PE=1 SV=4	41	18	17	0	539	57.9	7.83
High	P16278	Beta-galactosidase OS=Homo sapiens OX=9606 GN=GLB1 PE=1 SV=2	21	13	13	0	677	76	6.57
High	P13861	cAMP-dependent protein kinase type II-alpha regulatory subunit OS=Homo sapiens OX=9606 GN=PRKAR2A PE=1 SV=2	30	11	11	0	404	45.5	5.07
High	O75083	WD repeat-containing protein 1 OS=Homo sapiens OX=9606 GN=WDR1 PE=1 SV=4	23	11	11	0	606	66.2	6.65
High	A0A7I2V2R5	Dipeptidyl peptidase 4 OS=Homo sapiens OX=9606 GN=DPP4 PE=4 SV=1	20	17	17	0	734	84.5	5.87
High	E9PLK3	Aminopeptidase OS=Homo sapiens OX=9606 GN=NPEPPS PE=1 SV=1	22	17	17	0	915	102.9	5.6
High	P61019	Ras-related protein Rab-2A OS=Homo sapiens OX=9606 GN=RAB2A PE=1 SV=1	56	12	12	0	212	23.5	6.54
High	P13645	Keratin, type I cytoskeletal 10 OS=Homo sapiens OX=9606 GN=KRT10 PE=1 SV=6	25	18	15	3	584	58.8	5.21
High	P30153	Serine/threonine-protein phosphatase 2A 65 kDa regulatory subunit A alpha isoform OS=Homo sapiens OX=9606 GN=PPP2R1A PE=1 SV=4	26	13	9	4	589	65.3	5.11
High	O43175	D-3-phosphoglycerate dehydrogenase OS=Homo sapiens OX=9606 GN=PHGDH PE=1 SV=4	33	16	16	0	533	56.6	6.71
High	P07864	L-lactate dehydrogenase C chain OS=Homo sapiens OX=9606 GN=LDHC PE=1 SV=4	39	10	9	1	332	36.3	7.46
High	P25705	ATP synthase subunit alpha, mitochondrial OS=Homo sapiens OX=9606 GN=ATP5F1A PE=1 SV=1	26	12	12	0	553	59.7	9.13
High	Q8IZP9	Adhesion G-protein coupled receptor G2 OS=Homo sapiens OX=9606 GN=ADGRG2 PE=1 SV=2	10	9	9	0	1017	111.5	7.55
High	O00299	Chloride intracellular channel protein 1 OS=Homo sapiens OX=9606 GN=CLIC1 PE=1 SV=4	67	15	15	0	241	26.9	5.17
High	O00159	Unconventional myosin-Ic OS=Homo sapiens OX=9606 GN=MYO1C PE=1 SV=4	15	15	15	0	1063	121.6	9.41
High	P04075	Fructose-bisphosphate aldolase A OS=Homo sapiens OX=9606 GN=ALDOA PE=1 SV=2	37	13	12	1	364	39.4	8.09
High	P54107	Cysteine-rich secretory protein 1 OS=Homo sapiens OX=9606 GN=CRISP1 PE=1 SV=1	35	9	9	0	249	28.5	5.91
High	Q15833	Syntaxin-binding protein 2 OS=Homo sapiens OX=9606 GN=STXBP2 PE=1 SV=2	29	14	14	0	593	66.4	6.55
High	Q13200	26S proteasome non-ATPase regulatory subunit 2 OS=Homo sapiens OX=9606 GN=PSMD2 PE=1 SV=3	19	15	15	0	908	100.1	5.2
High	P17987	T-complex protein 1 subunit alpha OS=Homo sapiens OX=9606 GN=TCP1 PE=1 SV=1	27	13	13	0	556	60.3	6.11
High	P14550	Aldo-keto reductase family 1 member A1 OS=Homo sapiens OX=9606 GN=AKR1A1 PE=1 SV=3	45	12	11	1	325	36.6	6.79
High	P35052	Glypican-1 OS=Homo sapiens OX=9606 GN=GPC1 PE=1 SV=2	30	14	14	0	558	61.6	7.3
High	P09543	2',3'-cyclic-nucleotide 3'-phosphodiesterase OS=Homo sapiens OX=9606 GN=CNP PE=1 SV=2	32	16	15	1	421	47.5	9.07
High	A0A2R8Y5S7	Radixin OS=Homo sapiens OX=9606 GN=RDX PE=1 SV=1	20	17	5	0	590	69.3	6.27
High	Q9Y4L1	Hypoxia up-regulated protein 1 OS=Homo sapiens OX=9606 GN=HYOU1 PE=1 SV=1	15	13	12	1	999	111.3	5.22
High	P16152	Carbonyl reductase [NADPH] 1 OS=Homo sapiens OX=9606 GN=CBR1 PE=1 SV=3	48	12	12	0	277	30.4	8.32
High	P30041	Peroxiredoxin-6 OS=Homo sapiens OX=9606 GN=PRDX6 PE=1 SV=3	54	14	14	0	224	25	6.38
High	Q08431	Lactadherin OS=Homo sapiens OX=9606 GN=MFGE8 PE=1 SV=3	39	12	12	0	387	43.1	8.15
High	E5RI60	A disintegrin and metalloproteinase with thrombospondin motifs 1 OS=Homo sapiens OX=9606 GN=ADAMTS1 PE=1 SV=2	21	13	13	0	705	77.4	7.68
High	P06744	Glucose-6-phosphate isomerase OS=Homo sapiens OX=9606 GN=GPI PE=1 SV=4	24	12	12	0	558	63.1	8.32
High	P08133	Annexin A6 OS=Homo sapiens OX=9606 GN=ANXA6 PE=1 SV=3	30	18	18	0	673	75.8	5.6
High	P00367	Glutamate dehydrogenase 1, mitochondrial OS=Homo sapiens OX=9606 GN=GLUD1 PE=1 SV=2	24	10	10	0	558	61.4	7.8
High	Q9Y265	RuvB-like 1 OS=Homo sapiens OX=9606 GN=RUVBL1 PE=1 SV=1	33	12	12	0	456	50.2	6.42
High	Q8IYJ3	Synaptotagmin-like protein 1 OS=Homo sapiens OX=9606 GN=SYTL1 PE=1 SV=1	21	10	10	0	562	61.8	5.48
High	P31948	Stress-induced-phosphoprotein 1 OS=Homo sapiens OX=9606 GN=STIP1 PE=1 SV=1	26	14	14	0	543	62.6	6.8
High	C9JIZ6	Prosaposin OS=Homo sapiens OX=9606 GN=PSAP PE=1 SV=2	17	8	8	0	527	58.4	5.17
High	P38606	V-type proton ATPase catalytic subunit A OS=Homo sapiens OX=9606 GN=ATP6V1A PE=1 SV=2	27	14	14	0	617	68.3	5.52
High	O75487	Glypican-4 OS=Homo sapiens OX=9606 GN=GPC4 PE=1 SV=4	28	11	11	0	556	62.4	6.68
High	P46459	Vesicle-fusing ATPase OS=Homo sapiens OX=9606 GN=NSF PE=1 SV=3	23	17	17	0	744	82.5	6.95
High	P34913	Bifunctional epoxide hydrolase 2 OS=Homo sapiens OX=9606 GN=EPHX2 PE=1 SV=2	19	9	9	0	555	62.6	6.28
High	P48723	Heat shock 70 kDa protein 13 OS=Homo sapiens OX=9606 GN=HSPA13 PE=1 SV=1	23	9	9	0	471	51.9	5.76
High	Q13217	DnaJ homolog subfamily C member 3 OS=Homo sapiens OX=9606 GN=DNAJC3 PE=1 SV=1	30	15	15	0	504	57.5	6.15
High	P05787	Keratin, type II cytoskeletal 8 OS=Homo sapiens OX=9606 GN=KRT8 PE=1 SV=7	23	11	9	0	483	53.7	5.59
High	O43854	EGF-like repeat and discoidin I-like domain-containing protein 3 OS=Homo sapiens OX=9606 GN=EDIL3 PE=1 SV=1	29	14	14	0	480	53.7	7.28
High	P62937	Peptidyl-prolyl cis-trans isomerase A OS=Homo sapiens OX=9606 GN=PPIA PE=1 SV=2	41	8	8	0	165	18	7.81
High	Q6PCB0	von Willebrand factor A domain-containing protein 1 OS=Homo sapiens OX=9606 GN=VWA1 PE=1 SV=1	26	9	9	0	445	46.8	7.68

High	P49189	4-trimethylaminobutyraldehyde dehydrogenase OS=Homo sapiens OX=9606 GN=ALDH9A1 PE=1 SV=3	24	11	11	0	494	53.8	5.87
High	P25311	Zinc-alpha-2-glycoprotein OS=Homo sapiens OX=9606 GN=AZGP1 PE=1 SV=2	34	10	10	0	298	34.2	6.05
High	Q5JQC9	A-kinase anchor protein 4 OS=Homo sapiens OX=9606 GN=AKAP4 PE=1 SV=1	17	15	15	0	854	94.4	6.96
High	J3KS22	L-xylulose reductase (Fragment) OS=Homo sapiens OX=9606 GN=DCXR PE=1 SV=8	39	10	10	0	223	23.8	8.21
High	Q5T7F0	Neuropilin OS=Homo sapiens OX=9606 GN=NRP1 PE=1 SV=1	22	10	10	0	704	79	5.86
High	E9PFZ2	Ceruloplasmin OS=Homo sapiens OX=9606 GN=CP PE=1 SV=1	18	14	14	0	946	108.8	5.77
High	Q14152	Eukaryotic translation initiation factor 3 subunit A OS=Homo sapiens OX=9606 GN=EIF3A PE=1 SV=1	14	14	14	0	1382	166.5	6.79
High	P16403	Histone H1.2 OS=Homo sapiens OX=9606 GN=H1-2 PE=1 SV=2	35	10	3	7	213	21.4	10.93
High	A0A5F9ZHB4	Dipeptidase OS=Homo sapiens OX=9606 GN=DPEP3 PE=1 SV=1	15	6	6	0	513	56.4	8.75
High	P12429	Annexin A3 OS=Homo sapiens OX=9606 GN=ANXA3 PE=1 SV=3	46	13	13	0	323	36.4	5.92
High	P21266	Glutathione S-transferase Mu 3 OS=Homo sapiens OX=9606 GN=GSTM3 PE=1 SV=3	37	10	10	0	225	26.5	5.54
High	P07737	Profilin-1 OS=Homo sapiens OX=9606 GN=PFN1 PE=1 SV=2	47	7	7	0	140	15	8.27
High	A0A0G2JN42	Mucin-6 OS=Homo sapiens OX=9606 GN=MUC6 PE=1 SV=1	4	12	12	0	2439	256.9	7.39
High	P05154	Plasma serine protease inhibitor OS=Homo sapiens OX=9606 GN=SERPINA5 PE=1 SV=3	33	13	13	0	406	45.6	9.26
High	P00491	Purine nucleoside phosphorylase OS=Homo sapiens OX=9606 GN=PNP PE=1 SV=2	39	9	9	0	289	32.1	6.95
High	Q16610	Extracellular matrix protein 1 OS=Homo sapiens OX=9606 GN=ECM1 PE=1 SV=2	29	12	12	0	540	60.6	6.71
High	Q9BZQ8	Protein Niban 1 OS=Homo sapiens OX=9606 GN=NIBAN1 PE=1 SV=1	15	12	12	0	928	103.1	4.78
High	Q99536	Synaptic vesicle membrane protein VAT-1 homolog OS=Homo sapiens OX=9606 GN=VAT1 PE=1 SV=2	28	9	9	0	393	41.9	6.29
High	Q01813	ATP-dependent 6-phosphofructokinase, platelet type OS=Homo sapiens OX=9606 GN=PFKP PE=1 SV=2	16	12	11	1	784	85.5	7.55
High	Q3LXA3	Triokinase/FMN cyclase OS=Homo sapiens OX=9606 GN=TKFC PE=1 SV=2	28	12	12	0	575	58.9	7.49
High	A0A7I2V599	60 kDa heat shock protein, mitochondrial OS=Homo sapiens OX=9606 GN=HSPD1 PE=4 SV=1	16	9	9	0	537	57.8	7.77
High	E5RIV1	N-myc downstream-regulated gene 1 protein (Fragment) OS=Homo sapiens OX=9606 GN=NDRG1 PE=1 SV=1	25	3	3	0	193	21.2	4.65
High	P35237	Serpin B6 OS=Homo sapiens OX=9606 GN=SERPINB6 PE=1 SV=3	32	10	10	0	376	42.6	5.27
High	P21281	V-type proton ATPase subunit B, brain isoform OS=Homo sapiens OX=9606 GN=ATP6V1B2 PE=1 SV=3	20	10	10	0	511	56.5	5.81
High	P06396	Gelsolin OS=Homo sapiens OX=9606 GN=GSN PE=1 SV=1	19	16	10	5	782	85.6	6.28
High	P34932	Heat shock 70 kDa protein 4 OS=Homo sapiens OX=9606 GN=HSPA4 PE=1 SV=4	18	13	10	3	840	94.3	5.19
High	Q13228	Methanethiol oxidase OS=Homo sapiens OX=9606 GN=SELENBP1 PE=1 SV=2	26	11	11	0	472	52.4	6.37
High	Q06830	Peroxiredoxin-1 OS=Homo sapiens OX=9606 GN=PRDX1 PE=1 SV=1	37	11	9	2	199	22.1	8.13
High	P02647	Apolipoprotein A-I OS=Homo sapiens OX=9606 GN=APOA1 PE=1 SV=1	35	8	8	0	267	30.8	5.76
High	P23526	Adenosylhomocysteinase OS=Homo sapiens OX=9606 GN=AHCY PE=1 SV=4	26	10	9	1	432	47.7	6.34
High	P17174	Aspartate aminotransferase, cytoplasmic OS=Homo sapiens OX=9606 GN=GOT1 PE=1 SV=3	31	12	12	0	413	46.2	7.01
High	Q9NR45	Sialic acid synthase OS=Homo sapiens OX=9606 GN=NANS PE=1 SV=2	21	7	7	0	359	40.3	6.74
High	Q5QNW6	Histone H2B type 2-F OS=Homo sapiens OX=9606 GN=H2BC18 PE=1 SV=3	45	8	4	4	126	13.9	10.32
High	P13489	Ribonuclease inhibitor OS=Homo sapiens OX=9606 GN=RNH1 PE=1 SV=2	19	7	7	0	461	49.9	4.82
High	P11766	Alcohol dehydrogenase class-3 OS=Homo sapiens OX=9606 GN=ADH5 PE=1 SV=4	25	10	10	0	374	39.7	7.49
High	A0A024R6I7	Alpha-1-antitrypsin OS=Homo sapiens OX=9606 GN=SERPINA1 PE=1 SV=1	26	10	10	0	418	46.7	5.59
High	Q08380	Galectin-3-binding protein OS=Homo sapiens OX=9606 GN=LGALS3BP PE=1 SV=1	18	10	10	0	585	65.3	5.27
High	P10412	Histone H1.4 OS=Homo sapiens OX=9606 GN=H1-4 PE=1 SV=2	29	8	1	0	219	21.9	11.03
High	P60842	Eukaryotic initiation factor 4A-I OS=Homo sapiens OX=9606 GN=EIF4A1 PE=1 SV=1	17	5	1	5	406	46.1	5.48
High	P06576	ATP synthase subunit beta, mitochondrial OS=Homo sapiens OX=9606 GN=ATP5F1B PE=1 SV=3	19	7	7	0	529	56.5	5.4
High	P12036	Neurofilament heavy polypeptide OS=Homo sapiens OX=9606 GN=NEFH PE=1 SV=4	15	10	8	0	1026	112.4	6.18
High	X6RFL8	Ras-related protein Rab-14 (Fragment) OS=Homo sapiens OX=9606 GN=RAB14 PE=1 SV=1	47	8	7	0	181	20.4	6.33
High	H3BTI0	Cysteine-rich secretory protein LCCL domain-containing 2 OS=Homo sapiens OX=9606 GN=CRISPLD2 PE=1 SV=1	20	8	8	0	496	55.8	8.07
High	P16870	Carboxypeptidase E OS=Homo sapiens OX=9606 GN=CPE PE=1 SV=1	21	9	9	0	476	53.1	5.14
High	P31947	14-3-3 protein sigma OS=Homo sapiens OX=9606 GN=SFN PE=1 SV=1	31	8	4	0	248	27.8	4.74
High	A6NKB8	Aminopeptidase B OS=Homo sapiens OX=9606 GN=RNPEP PE=1 SV=1	17	10	10	0	611	68.1	6.01
High	Q9NQW7	Xaa-Pro aminopeptidase 1 OS=Homo sapiens OX=9606 GN=XPNPEP1 PE=1 SV=3	20	10	9	1	623	69.9	5.67
High	O60701	UDP-glucose 6-dehydrogenase OS=Homo sapiens OX=9606 GN=UGDH PE=1 SV=1	20	11	11	0	494	55	7.12
High	P45974	Ubiquitin carboxyl-terminal hydrolase 5 OS=Homo sapiens OX=9606 GN=USP5 PE=1 SV=2	14	9	9	0	858	95.7	5.03
High	P04792	Heat shock protein beta-1 OS=Homo sapiens OX=9606 GN=HSPB1 PE=1 SV=2	49	9	9	0	205	22.8	6.4
High	P43490	Nicotinamide phosphoribosyltransferase OS=Homo sapiens OX=9606 GN=NAMPT PE=1 SV=1	22	10	10	0	491	55.5	7.15
High	Q9Y5X9	Endothelial lipase OS=Homo sapiens OX=9606 GN=LIPG PE=1 SV=1	15	7	7	0	500	56.8	7.93
High	P35580	Myosin-10 OS=Homo sapiens OX=9606 GN=MYH10 PE=1 SV=3	5	11	1	1	1976	228.9	5.54
High	P47895	Aldehyde dehydrogenase family 1 member A3 OS=Homo sapiens OX=9606 GN=ALDH1A3 PE=1 SV=2	26	12	12	0	512	56.1	7.25
High	P31150	Rab GDP dissociation inhibitor alpha OS=Homo sapiens OX=9606 GN=GDI1 PE=1 SV=2	22	10	3	0	447	50.6	5.14
High	Q7Z304	MAM domain-containing protein 2 OS=Homo sapiens OX=9606 GN=MAMDC2 PE=1 SV=3	16	11	11	0	686	77.5	5.16
High	H7BZI1	Nucleobindin-1 (Fragment) OS=Homo sapiens OX=9606 GN=NUCB1 PE=1 SV=1	22	6	5	0	295	34.9	5.21
High	Q13438	Protein OS-9 OS=Homo sapiens OX=9606 GN=OS9 PE=1 SV=1	14	9	9	0	667	75.5	4.87
High	Q96BH3	Epididymal sperm-binding protein 1 OS=Homo sapiens OX=9606 GN=ELSPBP1 PE=1 SV=2	31	5	5	0	223	26.1	6.62
High	P62820	Ras-related protein Rab-1A OS=Homo sapiens OX=9606 GN=RAB1A PE=1 SV=3	49	10	3	6	205	22.7	6.21
High	E7EQG2	RNA helicase OS=Homo sapiens OX=9606 GN=EIF4A2 PE=1 SV=1	19	5	1	0	362	41.3	5.64
High	A0A3B3IUC4	Alpha-galactosidase OS=Homo sapiens OX=9606 GN=GLA PE=1 SV=1	17	8	8	0	470	53.2	5.86
High	A0A087WVM2	CD177 antigen OS=Homo sapiens OX=9606 GN=CD177 PE=1 SV=1	18	6	6	0	437	46.4	6.01
High	O95716	Ras-related protein Rab-3D OS=Homo sapiens OX=9606 GN=RAB3D PE=1 SV=1	47	8	5	2	219	24.3	4.93
High	Q9H0U4	Ras-related protein Rab-1B OS=Homo sapiens OX=9606 GN=RAB1B PE=1 SV=1	52	9	2	0	201	22.2	5.73
High	O95757	Heat shock 70 kDa protein 4L OS=Homo sapiens OX=9606 GN=HSPA4L PE=1 SV=3	12	9	6	1	839	94.5	5.88
High	P10253	Lysosomal alpha-glucosidase OS=Homo sapiens OX=9606 GN=GAA PE=1 SV=4	12	11	11	0	952	105.3	6
High	P62258	14-3-3 protein epsilon OS=Homo sapiens OX=9606 GN=YWHAE PE=1 SV=1	47	12	10	2	255	29.2	4.74
High	P27797	Calreticulin OS=Homo sapiens OX=9606 GN=CALR PE=1 SV=1	20	9	9	0	417	48.1	4.44
High	A0A1B0GUN9	Espin OS=Homo sapiens OX=9606 GN=ESPN PE=1 SV=1	7	6	6	0	1362	147.3	6.62
High	O60568	Multifunctional procollagen lysine hydroxylase and glycosyltransferase LH3 OS=Homo sapiens OX=9606 GN=PLOD3 PE=1 SV=1	15	10	10	0	738	84.7	6.05
High	P04004	Vitronectin OS=Homo sapiens OX=9606 GN=VTN PE=1 SV=1	14	5	5	0	478	54.3	5.8
High	A0A494C0G1	Phosphoacetylglucosamine mutase OS=Homo sapiens OX=9606 GN=PGM3 PE=1 SV=1	16	10	10	0	501	55.4	6.42
High	P11413	Glucose-6-phosphate 1-dehydrogenase OS=Homo sapiens OX=9606 GN=G6PD PE=1 SV=4	25	12	12	0	515	59.2	6.84
High	Q9BWD1	Acetyl-CoA acetyltransferase, cytosolic OS=Homo sapiens OX=9606 GN=ACAT2 PE=1 SV=2	25	7	7	0	397	41.3	6.92

High	A0A5F9UP49	45 kDa calcium-binding protein OS=Homo sapiens OX=9606 GN=SDF4 PE=1 SV=1	21	8	8	0	355	40.9	4.86
High	J3QRN6	Unconventional myosin-Id OS=Homo sapiens OX=9606 GN=MYO1D PE=1 SV=1	13	12	12	0	961	111.2	9.39
High	P09668	Pro-cathepsin H OS=Homo sapiens OX=9606 GN=CTSH PE=1 SV=4	23	7	7	0	335	37.4	8.07
High	P52565	Rho GDP-dissociation inhibitor 1 OS=Homo sapiens OX=9606 GN=ARHGDIA PE=1 SV=3	35	8	8	0	204	23.2	5.11
High	P26641	Elongation factor 1-gamma OS=Homo sapiens OX=9606 GN=EEF1G PE=1 SV=3	20	8	8	0	437	50.1	6.67
High	A0A590UK22	Unconventional myosin-VI (Fragment) OS=Homo sapiens OX=9606 GN=MYO6 PE=1 SV=1	12	9	9	0	878	100.4	8.56
High	H7BZJ3	Protein disulfide-isomerase A3 (Fragment) OS=Homo sapiens OX=9606 GN=PDIA3 PE=1 SV=1	51	7	1	0	123	13.5	7.3
High	P35527	Keratin, type I cytoskeletal 9 OS=Homo sapiens OX=9606 GN=KRT9 PE=1 SV=3	14	9	8	0	623	62	5.24
High	P02743	Serum amyloid P-component OS=Homo sapiens OX=9606 GN=APCS PE=1 SV=2	26	7	7	0	223	25.4	6.54
High	A0A7I2V3T0	CD9 antigen OS=Homo sapiens OX=9606 GN=CD9 PE=4 SV=1	27	5	5	0	216	24	5.38
High	Q02790	Peptidyl-prolyl cis-trans isomerase FKBP4 OS=Homo sapiens OX=9606 GN=FKBP4 PE=1 SV=3	24	8	8	0	459	51.8	5.43
High	A0A7I2YQK5	Inosine-5'-monophosphate dehydrogenase 2 OS=Homo sapiens OX=9606 GN=IMPDH2 PE=4 SV=1	16	7	6	1	513	55.5	7.02
High	Q8WXA2	Prostate and testis expressed protein 1 OS=Homo sapiens OX=9606 GN=PATE1 PE=1 SV=1	40	5	5	0	126	14.3	7.93
High	P40227	T-complex protein 1 subunit zeta OS=Homo sapiens OX=9606 GN=CCT6A PE=1 SV=3	20	11	10	1	531	58	6.68
High	O14773	Tripeptidyl-peptidase 1 OS=Homo sapiens OX=9606 GN=TPP1 PE=1 SV=2	18	7	7	0	563	61.2	6.48
High	Q14974	Importin subunit beta-1 OS=Homo sapiens OX=9606 GN=KPNB1 PE=1 SV=2	8	6	6	0	876	97.1	4.78
High	Q06210	Glutamine--fructose-6-phosphate aminotransferase [isomerizing] 1 OS=Homo sapiens OX=9606 GN=GFPT1 PE=1 SV=3	18	12	12	0	699	78.8	7.11
High	A0A1W2PQR6	Lysosome membrane protein 2 OS=Homo sapiens OX=9606 GN=SCARB2 PE=1 SV=1	20	6	6	0	449	51	5.16
High	P10644	cAMP-dependent protein kinase type I-alpha regulatory subunit OS=Homo sapiens OX=9606 GN=PRKAR1A PE=1 SV=1	22	7	7	0	381	43	5.35
High	P20142	Gastricsin OS=Homo sapiens OX=9606 GN=PGC PE=1 SV=1	10	5	5	0	388	42.4	4.46
High	P60174	Triosephosphate isomerase OS=Homo sapiens OX=9606 GN=TPI1 PE=1 SV=4	44	9	9	0	249	26.7	6.9
High	E5RIA4	Aspartyl aminopeptidase OS=Homo sapiens OX=9606 GN=DNPEP PE=1 SV=1	17	6	6	0	471	52	7.42
High	Q16777	Histone H2A type 2-C OS=Homo sapiens OX=9606 GN=H2AC20 PE=1 SV=4	36	5	2	3	129	14	10.9
High	P23381	Tryptophan--tRNA ligase, cytoplasmic OS=Homo sapiens OX=9606 GN=WARS1 PE=1 SV=2	19	7	7	0	471	53.1	6.23
High	P49913	Cathelicidin antimicrobial peptide OS=Homo sapiens OX=9606 GN=CAMP PE=1 SV=1	22	5	5	0	170	19.3	9.41
High	Q9BR76	Coronin-1B OS=Homo sapiens OX=9606 GN=CORO1B PE=1 SV=1	18	9	9	0	489	54.2	5.88
High	P51159	Ras-related protein Rab-27A OS=Homo sapiens OX=9606 GN=RAB27A PE=1 SV=3	33	7	5	2	221	24.9	5.22
High	P16401	Histone H1.5 OS=Homo sapiens OX=9606 GN=H1-5 PE=1 SV=3	30	7	6	0	226	22.6	10.92
High	Q14914	Prostaglandin reductase 1 OS=Homo sapiens OX=9606 GN=PTGR1 PE=1 SV=2	22	7	7	0	329	35.8	8.29
High	P21810	Biglycan OS=Homo sapiens OX=9606 GN=BGN PE=1 SV=2	23	7	6	1	368	41.6	7.52
High	A0A024R571	EH domain-containing protein 1 OS=Homo sapiens OX=9606 GN=EHD1 PE=1 SV=1	17	8	7	0	548	61.9	6.71
High	P80723	Brain acid soluble protein 1 OS=Homo sapiens OX=9606 GN=BASP1 PE=1 SV=2	40	7	7	0	227	22.7	4.63
High	P33176	Kinesin-1 heavy chain OS=Homo sapiens OX=9606 GN=KIF5B PE=1 SV=1	9	8	4	3	963	109.6	6.51
High	P55884	Eukaryotic translation initiation factor 3 subunit B OS=Homo sapiens OX=9606 GN=EIF3B PE=1 SV=3	8	6	6	0	814	92.4	5
High	Q9BV36	Melanophilin OS=Homo sapiens OX=9606 GN=MLPH PE=1 SV=1	13	6	6	0	600	65.9	6.07
High	P61026	Ras-related protein Rab-10 OS=Homo sapiens OX=9606 GN=RAB10 PE=1 SV=1	39	8	5	1	200	22.5	8.38
High	G5E9W0	Phospholipase A1 member A OS=Homo sapiens OX=9606 GN=PLA1A PE=1 SV=1	16	7	7	0	440	48.1	7.72
High	Q99574	Neuroserpin OS=Homo sapiens OX=9606 GN=SERPINI1 PE=1 SV=1	13	7	7	0	410	46.4	4.91
High	P13647	Keratin, type II cytoskeletal 5 OS=Homo sapiens OX=9606 GN=KRT5 PE=1 SV=3	10	7	2	0	590	62.3	7.74
High	P31153	S-adenosylmethionine synthase isoform type-2 OS=Homo sapiens OX=9606 GN=MAT2A PE=1 SV=1	21	7	6	1	395	43.6	6.48
High	P63104	14-3-3 protein zeta/delta OS=Homo sapiens OX=9606 GN=YWHAZ PE=1 SV=1	29	9	4	4	245	27.7	4.79
High	O00339	Matrilin-2 OS=Homo sapiens OX=9606 GN=MATN2 PE=1 SV=4	12	10	10	0	956	106.8	6.27
High	P35998	26S proteasome regulatory subunit 7 OS=Homo sapiens OX=9606 GN=PSMC2 PE=1 SV=3	21	8	8	0	433	48.6	5.95
High	P32119	Peroxiredoxin-2 OS=Homo sapiens OX=9606 GN=PRDX2 PE=1 SV=5	33	10	10	0	198	21.9	5.97
High	E9PK47	Alpha-1,4 glucan phosphorylase OS=Homo sapiens OX=9606 GN=PYGL PE=1 SV=1	9	10	2	0	819	94	7.31
High	P17612	cAMP-dependent protein kinase catalytic subunit alpha OS=Homo sapiens OX=9606 GN=PRKACA PE=1 SV=2	15	8	8	0	351	40.6	8.79
High	P13798	Acylamino-acid-releasing enzyme OS=Homo sapiens OX=9606 GN=APEH PE=1 SV=4	8	6	6	0	732	81.2	5.48
High	P04066	Tissue alpha-L-fucosidase OS=Homo sapiens OX=9606 GN=FUCA1 PE=1 SV=4	15	7	7	0	466	53.7	6.84
High	Q9HB40	Retinoid-inducible serine carboxypeptidase OS=Homo sapiens OX=9606 GN=SCPEP1 PE=1 SV=1	19	7	7	0	452	50.8	5.81
High	H7C0V9	Amyloid-beta A4 protein (Fragment) OS=Homo sapiens OX=9606 GN=APP PE=1 SV=1	14	6	6	0	485	55.1	4.82
High	P01857	Immunoglobulin heavy constant gamma 1 OS=Homo sapiens OX=9606 GN=IGHG1 PE=1 SV=1	28	7	5	2	330	36.1	8.19
High	A0A5F9ZHM4	L-lactate dehydrogenase OS=Homo sapiens OX=9606 GN=LDHB PE=1 SV=1	26	8	7	0	341	37.4	6.25
High	C9JXA5	Guanine nucleotide-binding protein G(I)/G(S)/G(T) subunit beta-2 (Fragment) OS=Homo sapiens OX=9606 GN=GNB2 PE=1 SV=1	25	6	2	4	251	27.5	6.76
High	Q9NQ79	Cartilage acidic protein 1 OS=Homo sapiens OX=9606 GN=CRTAC1 PE=1 SV=2	14	10	10	0	661	71.4	5.12
High	P54578	Ubiquitin carboxyl-terminal hydrolase 14 OS=Homo sapiens OX=9606 GN=USP14 PE=1 SV=3	15	7	7	0	494	56	5.3
High	H0Y8C6	Importin-5 (Fragment) OS=Homo sapiens OX=9606 GN=IPO5 PE=1 SV=1	8	7	7	0	1099	123.8	5.06
High	Q92598	Heat shock protein 105 kDa OS=Homo sapiens OX=9606 GN=HSPH1 PE=1 SV=1	8	6	2	0	858	96.8	5.39
High	P50995	Annexin A11 OS=Homo sapiens OX=9606 GN=ANXA11 PE=1 SV=1	17	7	7	0	505	54.4	7.65
High	P02794	Ferritin heavy chain OS=Homo sapiens OX=9606 GN=FTH1 PE=1 SV=2	26	7	7	0	183	21.2	5.55
High	Q9H223	EH domain-containing protein 4 OS=Homo sapiens OX=9606 GN=EHD4 PE=1 SV=1	14	8	7	1	541	61.1	6.76
High	Q5T0I0	Macrophage-capping protein (Fragment) OS=Homo sapiens OX=9606 GN=GSN PE=1 SV=1	25	7	1	0	260	28.9	7.9
High	B1AK87	F-actin-capping protein subunit beta OS=Homo sapiens OX=9606 GN=CAPZB PE=1 SV=2	18	4	4	0	244	27.4	5.55
High	P19440	Glutathione hydrolase 1 proenzyme OS=Homo sapiens OX=9606 GN=GGT1 PE=1 SV=2	14	8	8	0	569	61.4	7.12
High	Q13618	Cullin-3 OS=Homo sapiens OX=9606 GN=CUL3 PE=1 SV=2	8	8	8	0	768	88.9	8.48
High	E9PBS1	AIR carboxylase (Fragment) OS=Homo sapiens OX=9606 GN=PAICS PE=1 SV=1	17	5	5	0	413	45.6	6.68
High	Q01518	Adenylyl cyclase-associated protein 1 OS=Homo sapiens OX=9606 GN=CAP1 PE=1 SV=5	14	6	6	0	475	51.9	8.06
High	P61981	14-3-3 protein gamma OS=Homo sapiens OX=9606 GN=YWHAG PE=1 SV=2	23	7	2	0	247	28.3	4.89
High	Q7L7L0	Histone H2A type 3 OS=Homo sapiens OX=9606 GN=H2AW PE=1 SV=3	35	4	1	0	130	14.1	11.05
High	G3V3U4	Proteasome subunit alpha type OS=Homo sapiens OX=9606 GN=PSMA6 PE=1 SV=1	48	5	5	0	107	11.6	9.1
High	P52788	Spermine synthase OS=Homo sapiens OX=9606 GN=SMS PE=1 SV=2	19	6	6	0	366	41.2	5.02
High	P28907	ADP-ribosyl cyclase/cyclic ADP-ribose hydrolase 1 OS=Homo sapiens OX=9606 GN=CD38 PE=1 SV=2	20	6	6	0	300	34.3	7.66
High	Q92526	T-complex protein 1 subunit zeta-2 OS=Homo sapiens OX=9606 GN=CCT6B PE=1 SV=5	11	6	5	0	530	57.8	7.24
High	A0A494C1B8	Tripeptidyl-peptidase 2 OS=Homo sapiens OX=9606 GN=TPP2 PE=1 SV=1	8	9	9	0	1173	130.2	6.29
High	J3QTR3	Ubiquitin-40S ribosomal protein S27a (Fragment) OS=Homo sapiens OX=9606 GN=RPS27A PE=1 SV=1	38	4	4	0	106	12.2	10.14

High	E9PQB7	Cofilin, non-muscle isoform (Fragment) OS=Homo sapiens OX=9606 GN=CFL1 PE=1 SV=1	55	6	5	0	122	13.9	8.72
High	P22748	Carbonic anhydrase 4 OS=Homo sapiens OX=9606 GN=CA4 PE=1 SV=2	23	7	7	0	312	35	7.83
High	Q92743	Serine protease HTRA1 OS=Homo sapiens OX=9606 GN=HTRA1 PE=1 SV=1	11	5	5	0	480	51.3	7.83
High	P47755	F-actin-capping protein subunit alpha-2 OS=Homo sapiens OX=9606 GN=CAPZA2 PE=1 SV=3	20	4	3	1	286	32.9	5.85
High	P09960	Leukotriene A-4 hydrolase OS=Homo sapiens OX=9606 GN=LTA4H PE=1 SV=2	14	7	7	0	611	69.2	6.18
High	P52209	6-phosphogluconate dehydrogenase, decarboxylating OS=Homo sapiens OX=9606 GN=PGD PE=1 SV=3	24	8	8	0	483	53.1	7.23
High	P07384	Calpain-1 catalytic subunit OS=Homo sapiens OX=9606 GN=CAPN1 PE=1 SV=1	11	8	8	0	714	81.8	5.67
High	P30086	Phosphatidylethanolamine-binding protein 1 OS=Homo sapiens OX=9606 GN=PEBP1 PE=1 SV=3	42	6	6	0	187	21	7.53
High	O00194	Ras-related protein Rab-27B OS=Homo sapiens OX=9606 GN=RAB27B PE=1 SV=4	26	6	4	0	218	24.6	5.52
High	O14556	Glyceraldehyde-3-phosphate dehydrogenase, testis-specific OS=Homo sapiens OX=9606 GN=GAPDHS PE=1 SV=2	16	5	4	0	408	44.5	8.19
High	P28074	Proteasome subunit beta type-5 OS=Homo sapiens OX=9606 GN=PSMB5 PE=1 SV=3	19	4	4	0	263	28.5	6.92
High	O75131	Copine-3 OS=Homo sapiens OX=9606 GN=CPNE3 PE=1 SV=1	12	6	6	0	537	60.1	5.85
High	P27348	14-3-3 protein theta OS=Homo sapiens OX=9606 GN=YWHAQ PE=1 SV=1	24	7	3	0	245	27.7	4.78
High	Q96TA1	Protein Niban 2 OS=Homo sapiens OX=9606 GN=NIBAN2 PE=1 SV=3	9	7	7	0	746	84.1	6.19
High	Q08629	Testican-1 OS=Homo sapiens OX=9606 GN=SPOCK1 PE=1 SV=1	11	5	4	1	439	49.1	6.1
High	F5GZS6	4F2 cell-surface antigen heavy chain OS=Homo sapiens OX=9606 GN=SLC3A2 PE=1 SV=1	11	7	7	0	599	64.8	5.1
High	P62873	Guanine nucleotide-binding protein G(I)/G(S)/G(T) subunit beta-1 OS=Homo sapiens OX=9606 GN=GNB1 PE=1 SV=3	15	5	1	0	340	37.4	6
High	P54802	Alpha-N-acetylglucosaminidase OS=Homo sapiens OX=9606 GN=NAGLU PE=1 SV=2	13	6	6	0	743	82.2	6.65
High	A0A0C4DGN4	Zymogen granule protein 16 homolog B OS=Homo sapiens OX=9606 GN=ZG16B PE=1 SV=1	26	5	5	0	178	19.6	5.95
High	P31946	14-3-3 protein beta/alpha OS=Homo sapiens OX=9606 GN=YWHAB PE=1 SV=3	19	7	1	2	246	28.1	4.83
High	O95793	Double-stranded RNA-binding protein Staufen homolog 1 OS=Homo sapiens OX=9606 GN=STAU1 PE=1 SV=2	9	5	5	0	577	63.1	9.44
High	A0A7I2V492	Kinesin heavy chain isoform 5C OS=Homo sapiens OX=9606 GN=KIF5C PE=4 SV=1	7	6	2	0	956	109.3	6.14
High	Q96IU4	Protein ABHD14B OS=Homo sapiens OX=9606 GN=ABHD14B PE=1 SV=1	16	3	3	0	210	22.3	6.4
High	O00391	Sulfhydryl oxidase 1 OS=Homo sapiens OX=9606 GN=QSOX1 PE=1 SV=3	10	6	6	0	747	82.5	8.92
High	Q7Z406	Myosin-14 OS=Homo sapiens OX=9606 GN=MYH14 PE=1 SV=2	3	7	2	0	1995	227.7	5.6
High	O94813	Slit homolog 2 protein OS=Homo sapiens OX=9606 GN=SLIT2 PE=1 SV=1	5	8	8	0	1529	169.8	7.06
High	P20337	Ras-related protein Rab-3B OS=Homo sapiens OX=9606 GN=RAB3B PE=1 SV=2	33	7	4	0	219	24.7	5.02
High	A0AVT1	Ubiquitin-like modifier-activating enzyme 6 OS=Homo sapiens OX=9606 GN=UBA6 PE=1 SV=1	7	7	7	0	1052	117.9	6.14
High	Q9BY14	Testis-expressed protein 101 OS=Homo sapiens OX=9606 GN=TEX101 PE=1 SV=2	16	3	3	0	249	26.6	4.94
High	H0YCV9	CD44 antigen (Fragment) OS=Homo sapiens OX=9606 GN=CD44 PE=1 SV=2	14	4	4	0	277	30.6	5.3
High	P42224	Signal transducer and activator of transcription 1-alpha/beta OS=Homo sapiens OX=9606 GN=STAT1 PE=1 SV=2	8	5	5	0	750	87.3	6.05
High	P11279	Lysosome-associated membrane glycoprotein 1 OS=Homo sapiens OX=9606 GN=LAMP1 PE=1 SV=3	10	5	5	0	417	44.9	8.75
High	P17405	Sphingomyelin phosphodiesterase OS=Homo sapiens OX=9606 GN=SMPD1 PE=1 SV=5	14	8	8	0	631	69.9	7.28
High	P01871	Immunoglobulin heavy constant mu OS=Homo sapiens OX=9606 GN=IGHM PE=1 SV=4	11	4	4	0	453	49.4	6.77
High	P01876	Immunoglobulin heavy constant alpha 1 OS=Homo sapiens OX=9606 GN=IGHA1 PE=1 SV=2	20	7	2	4	353	37.6	6.51
High	P00390	Glutathione reductase, mitochondrial OS=Homo sapiens OX=9606 GN=GSR PE=1 SV=2	14	8	8	0	522	56.2	8.5
High	E9PDF6	Unconventional myosin-Ib OS=Homo sapiens OX=9606 GN=MYO1B PE=1 SV=1	6	6	5	0	1107	128.4	9.31
High	P62491	Ras-related protein Rab-11A OS=Homo sapiens OX=9606 GN=RAB11A PE=1 SV=3	33	7	7	0	216	24.4	6.57
High	P08253	72 kDa type IV collagenase OS=Homo sapiens OX=9606 GN=MMP2 PE=1 SV=2	11	7	7	0	660	73.8	5.47
High	P27824	Calnexin OS=Homo sapiens OX=9606 GN=CANX PE=1 SV=2	8	4	4	0	592	67.5	4.6
High	A0A0A0MR02	Outer mitochondrial membrane protein porin 2 (Fragment) OS=Homo sapiens OX=9606 GN=VDAC2 PE=1 SV=1	21	5	5	0	282	30.3	7.81
High	H0YBL1	Inositol-1-monophosphatase (Fragment) OS=Homo sapiens OX=9606 GN=IMPA1 PE=1 SV=1	31	7	7	0	265	28.6	6.21
High	Q15046	Lysine--tRNA ligase OS=Homo sapiens OX=9606 GN=KARS1 PE=1 SV=3	11	6	6	0	597	68	6.35
High	Q5JR08	Rho-related GTP-binding protein RhoC (Fragment) OS=Homo sapiens OX=9606 GN=RHOC PE=1 SV=8	30	5	1	0	188	21.5	6.58
High	P53621	Coatomer subunit alpha OS=Homo sapiens OX=9606 GN=COPA PE=1 SV=2	5	5	5	0	1224	138.3	7.66
High	P54136	Arginine--tRNA ligase, cytoplasmic OS=Homo sapiens OX=9606 GN=RARS1 PE=1 SV=2	10	6	6	0	660	75.3	6.68
High	A0A0G2JLB3	Glucosylceramidase OS=Homo sapiens OX=9606 GN=GBA PE=1 SV=1	8	4	4	0	536	59.6	7.42
High	H7BY55	Complement decay-accelerating factor OS=Homo sapiens OX=9606 GN=CD55 PE=1 SV=2	7	4	4	0	550	58.9	8.78
High	Q9H173	Nucleotide exchange factor SIL1 OS=Homo sapiens OX=9606 GN=SIL1 PE=1 SV=1	14	7	7	0	461	52.1	5.36
High	P62241	40S ribosomal protein S8 OS=Homo sapiens OX=9606 GN=RPS8 PE=1 SV=2	19	4	4	0	208	24.2	10.32
High	J3QRU1	Tyrosine-protein kinase OS=Homo sapiens OX=9606 GN=YES1 PE=1 SV=1	12	7	2	5	548	61.3	6.57
High	P07814	Bifunctional glutamate/proline--tRNA ligase OS=Homo sapiens OX=9606 GN=EPRS1 PE=1 SV=5	4	6	6	0	1512	170.5	7.33
High	P50502	Hsc70-interacting protein OS=Homo sapiens OX=9606 GN=ST13 PE=1 SV=2	14	6	6	0	369	41.3	5.27
High	P14384	Carboxypeptidase M OS=Homo sapiens OX=9606 GN=CPM PE=1 SV=2	17	7	7	0	443	50.5	7.36
High	Q15631	Translin OS=Homo sapiens OX=9606 GN=TSN PE=1 SV=1	16	3	3	0	228	26.2	6.44
High	P19012	Keratin, type I cytoskeletal 15 OS=Homo sapiens OX=9606 GN=KRT15 PE=1 SV=3	10	6	2	1	456	49.2	4.77
High	Q15084	Protein disulfide-isomerase A6 OS=Homo sapiens OX=9606 GN=PDIA6 PE=1 SV=1	10	3	3	0	440	48.1	5.08
High	P08727	Keratin, type I cytoskeletal 19 OS=Homo sapiens OX=9606 GN=KRT19 PE=1 SV=4	11	6	1	1	400	44.1	5.14
High	A0A3B3ITK7	Phosphoglucomutase-1 OS=Homo sapiens OX=9606 GN=PGM1 PE=1 SV=1	11	6	6	0	584	63.9	6.76
High	P28838	Cytosol aminopeptidase OS=Homo sapiens OX=9606 GN=LAP3 PE=1 SV=3	12	6	6	0	519	56.1	7.93
High	I3L1D5	Polyunsaturated fatty acid lipoxygenase ALOX15B OS=Homo sapiens OX=9606 GN=ALOX15B PE=1 SV=1	12	7	7	0	664	74.4	6.18
High	P25789	Proteasome subunit alpha type-4 OS=Homo sapiens OX=9606 GN=PSMA4 PE=1 SV=1	16	4	4	0	261	29.5	7.72
High	P01033	Metalloproteinase inhibitor 1 OS=Homo sapiens OX=9606 GN=TIMP1 PE=1 SV=1	13	2	2	0	207	23.2	8.1
High	P51153	Ras-related protein Rab-13 OS=Homo sapiens OX=9606 GN=RAB13 PE=1 SV=1	22	4	2	0	203	22.8	9.19
High	O43776	Asparagine--tRNA ligase, cytoplasmic OS=Homo sapiens OX=9606 GN=NARS1 PE=1 SV=1	5	2	2	0	548	62.9	6.25
High	P29401	Transketolase OS=Homo sapiens OX=9606 GN=TKT PE=1 SV=3	9	6	6	0	623	67.8	7.66
High	A0A1B0GTG2	Alpha-aminoadipic semialdehyde dehydrogenase OS=Homo sapiens OX=9606 GN=ALDH7A1 PE=1 SV=1	5	2	2	0	536	58.1	8.13
High	Q93099	Homogentisate 1,2-dioxygenase OS=Homo sapiens OX=9606 GN=HGD PE=1 SV=2	13	4	4	0	445	49.9	6.96
High	P61586	Transforming protein RhoA OS=Homo sapiens OX=9606 GN=RHOA PE=1 SV=1	30	5	1	4	193	21.8	6.1
High	O00204	Sulfotransferase 2B1 OS=Homo sapiens OX=9606 GN=SULT2B1 PE=1 SV=2	12	4	4	0	365	41.3	5.4
High	Q8N335	Glycerol-3-phosphate dehydrogenase 1-like protein OS=Homo sapiens OX=9606 GN=GPD1L PE=1 SV=1	21	7	7	0	351	38.4	7.02
High	Q15435	Protein phosphatase 1 regulatory subunit 7 OS=Homo sapiens OX=9606 GN=PPP1R7 PE=1 SV=1	16	7	7	0	360	41.5	4.91

High	Q9H3U7	SPARC-related modular calcium-binding protein 2 OS=Homo sapiens OX=9606 GN=SMOC2 PE=1 SV=2	10	4	4	0	446	49.6	8.46
High	Q9Y617	Phosphoserine aminotransferase OS=Homo sapiens OX=9606 GN=PSAT1 PE=1 SV=2	16	6	6	0	370	40.4	7.66
High	Q9Y490	Talin-1 OS=Homo sapiens OX=9606 GN=TLN1 PE=1 SV=3	2	5	5	0	2541	269.6	6.07
High	E9PH38	Serine/threonine-protein phosphatase 2A 65 kDa regulatory subunit A alpha isoform (Fragment) OS=Homo sapiens OX=9606 GN=PPP2R1A PE=1 SV=2	46	5	1	0	132	14.6	8.44
High	Q08209	Serine/threonine-protein phosphatase 2B catalytic subunit alpha isoform OS=Homo sapiens OX=9606 GN=PPP3CA PE=1 SV=1	11	7	4	2	521	58.7	5.86
High	P0C8F1	Prostate and testis expressed protein 4 OS=Homo sapiens OX=9606 GN=PATE4 PE=2 SV=2	32	5	5	0	98	11.4	8.62
High	P09211	Glutathione S-transferase P OS=Homo sapiens OX=9606 GN=GSTP1 PE=1 SV=2	32	5	5	0	210	23.3	5.64
High	Q99519	Sialidase-1 OS=Homo sapiens OX=9606 GN=NEU1 PE=1 SV=1	13	5	5	0	415	45.4	5.88
High	P49788	Retinoic acid receptor responder protein 1 OS=Homo sapiens OX=9606 GN=RARRES1 PE=1 SV=2	22	6	6	0	294	33.3	8.51
High	F6RFD5	Actin-depolymerizing factor OS=Homo sapiens OX=9606 GN=DSTN PE=1 SV=1	41	6	5	1	135	15.4	8.59
High	C9JH19	Cathepsin D OS=Homo sapiens OX=9606 GN=CTSD PE=1 SV=2	11	5	5	0	451	48.6	7.84
High	P35475	Alpha-L-iduronidase OS=Homo sapiens OX=9606 GN=IDUA PE=1 SV=2	11	6	6	0	653	72.6	9.14
High	P12931	Proto-oncogene tyrosine-protein kinase Src OS=Homo sapiens OX=9606 GN=SRC PE=1 SV=3	11	6	2	0	536	59.8	7.42
High	P62701	40S ribosomal protein S4, X isoform OS=Homo sapiens OX=9606 GN=RPS4X PE=1 SV=2	16	5	5	0	263	29.6	10.15
High	Q9BWS9	Chitinase domain-containing protein 1 OS=Homo sapiens OX=9606 GN=CHID1 PE=1 SV=1	11	4	4	0	393	44.9	8.63
High	Q9Y3R5	Protein dopey-2 OS=Homo sapiens OX=9606 GN=DOP1B PE=1 SV=5	2	6	6	0	2298	258.1	6.29
High	O00232	26S proteasome non-ATPase regulatory subunit 12 OS=Homo sapiens OX=9606 GN=PSMD12 PE=1 SV=3	15	8	8	0	456	52.9	7.65
High	Q13162	Peroxiredoxin-4 OS=Homo sapiens OX=9606 GN=PRDX4 PE=1 SV=1	9	3	1	0	271	30.5	6.29
High	P23284	Peptidyl-prolyl cis-trans isomerase B OS=Homo sapiens OX=9606 GN=PPIB PE=1 SV=2	27	6	6	0	216	23.7	9.41
High	H0YAF8	Receptor of-activated protein C kinase 1 (Fragment) OS=Homo sapiens OX=9606 GN=RACK1 PE=1 SV=1	19	3	3	0	198	22	6.33
High	Q92542	Nicastrin OS=Homo sapiens OX=9606 GN=NCSTN PE=1 SV=2	8	6	6	0	709	78.4	5.99
High	P30043	Flavin reductase (NADPH) OS=Homo sapiens OX=9606 GN=BLVRB PE=1 SV=3	11	3	3	0	206	22.1	7.65
High	Q9NZM1	Myoferlin OS=Homo sapiens OX=9606 GN=MYOF PE=1 SV=1	3	6	6	0	2061	234.6	6.18
High	A0A0A0MTJ2	Sparc/osteonectin, cwcv and kazal-like domains proteoglycan (Testican) 3, isoform CRA_b OS=Homo sapiens OX=9606 GN=SPOCK3 PE=1 SV=1	13	4	3	0	344	39.4	5.02
High	P47897	Glutamine--tRNA ligase OS=Homo sapiens OX=9606 GN=QARS1 PE=1 SV=1	9	7	7	0	775	87.7	7.15
High	Q96QK1	Vacuolar protein sorting-associated protein 35 OS=Homo sapiens OX=9606 GN=VPS35 PE=1 SV=2	7	5	5	0	796	91.6	5.49
High	O76054	SEC14-like protein 2 OS=Homo sapiens OX=9606 GN=SEC14L2 PE=1 SV=1	14	5	5	0	403	46.1	7.84
High	A0A0G2JMB2	Immunoglobulin heavy constant alpha 2 (Fragment) OS=Homo sapiens OX=9606 GN=IGHA2 PE=1 SV=1	19	6	1	0	340	36.5	6.1
High	P62753	40S ribosomal protein S6 OS=Homo sapiens OX=9606 GN=RPS6 PE=1 SV=1	22	4	4	0	249	28.7	10.84
High	A0A1B0GUE3	Acid ceramidase OS=Homo sapiens OX=9606 GN=ASAH1 PE=1 SV=1	15	3	3	0	330	37.4	6.79
High	A0A6Q8PHH9	Diadenosine tetraphosphate synthetase OS=Homo sapiens OX=9606 GN=GARS1 PE=1 SV=1	8	5	5	0	705	79.3	6.7
High	P00338	L-lactate dehydrogenase A chain OS=Homo sapiens OX=9606 GN=LDHA PE=1 SV=2	15	5	4	0	332	36.7	8.27
High	Q14894	Ketimine reductase mu-crystallin OS=Homo sapiens OX=9606 GN=CRYM PE=1 SV=1	13	4	4	0	314	33.8	5.14
High	A0A494C0X7	Integrin beta OS=Homo sapiens OX=9606 GN=ITGB2 PE=1 SV=1	6	4	4	0	769	84.7	6.98
High	Q5T8U3	60S ribosomal protein L7a (Fragment) OS=Homo sapiens OX=9606 GN=RPL7A PE=1 SV=1	17	4	4	0	191	21.5	11.02
High	Q96A08	Histone H2B type 1-A OS=Homo sapiens OX=9606 GN=H2BC1 PE=1 SV=3	32	5	3	0	127	14.2	10.32
High	Q92485	Acid sphingomyelinase-like phosphodiesterase 3b OS=Homo sapiens OX=9606 GN=SMPDL3B PE=1 SV=2	11	4	4	0	455	50.8	5.64
High	P14868	Aspartate--tRNA ligase, cytoplasmic OS=Homo sapiens OX=9606 GN=DARS1 PE=1 SV=2	14	6	6	0	501	57.1	6.55
High	H0YF32	40S ribosomal protein S3 (Fragment) OS=Homo sapiens OX=9606 GN=RPS3 PE=1 SV=1	39	4	4	0	123	13.5	8
High	B5MDF5	GTP-binding nuclear protein Ran OS=Homo sapiens OX=9606 GN=RAN PE=1 SV=1	27	6	6	0	233	26.2	7.01
High	Q5JWF2	Guanine nucleotide-binding protein G(s) subunit alpha isoforms XLas OS=Homo sapiens OX=9606 GN=GNAS PE=1 SV=2	6	6	5	1	1037	111	5.03
High	P10323	Acrosin OS=Homo sapiens OX=9606 GN=ACR PE=1 SV=4	12	5	5	0	421	45.8	9.07
High	P11908	Ribose-phosphate pyrophosphokinase 2 OS=Homo sapiens OX=9606 GN=PRPS2 PE=1 SV=2	12	4	4	0	318	34.7	6.61
High	P49257	Protein ERGIC-53 OS=Homo sapiens OX=9606 GN=LMAN1 PE=1 SV=2	4	3	3	0	510	57.5	6.77
High	C9J5Y1	Transmembrane protease serine 2 OS=Homo sapiens OX=9606 GN=TMPRSS2 PE=1 SV=2	6	5	5	0	452	49.7	8.34
High	K7ELW0	Parkinson disease protein 7 OS=Homo sapiens OX=9606 GN=PARK7 PE=1 SV=1	37	6	6	0	169	17.9	7.87
High	Q10567	AP-1 complex subunit beta-1 OS=Homo sapiens OX=9606 GN=AP1B1 PE=1 SV=2	7	7	2	5	949	104.6	5.06
High	P61769	Beta-2-microglobulin OS=Homo sapiens OX=9606 GN=B2M PE=1 SV=1	35	3	3	0	119	13.7	6.52
High	A0A1B0GVS3	Alpha-centractin OS=Homo sapiens OX=9606 GN=ACTR1A PE=1 SV=1	30	4	4	0	221	24.9	6.21
High	Q8NHP8	Putative phospholipase B-like 2 OS=Homo sapiens OX=9606 GN=PLBD2 PE=1 SV=2	10	6	6	0	589	65.4	6.8
High	Q02750	Dual specificity mitogen-activated protein kinase kinase 1 OS=Homo sapiens OX=9606 GN=MAP2K1 PE=1 SV=2	8	3	2	0	393	43.4	6.62
High	P01834	Immunoglobulin kappa constant OS=Homo sapiens OX=9606 GN=IGKC PE=1 SV=2	48	3	3	0	107	11.8	6.52
High	P11586	C-1-tetrahydrofolate synthase, cytoplasmic OS=Homo sapiens OX=9606 GN=MTHFD1 PE=1 SV=3	5	4	4	0	935	101.5	7.3
High	Q9BS26	Endoplasmic reticulum resident protein 44 OS=Homo sapiens OX=9606 GN=ERP44 PE=1 SV=1	13	4	3	1	406	46.9	5.26
High	Q6DN03	Putative histone H2B type 2-C OS=Homo sapiens OX=9606 GN=H2BC20P PE=5 SV=3	20	5	3	0	193	21.5	10.7
High	O43242	26S proteasome non-ATPase regulatory subunit 3 OS=Homo sapiens OX=9606 GN=PSMD3 PE=1 SV=2	7	4	4	0	534	60.9	8.44
High	F8W1S1	Keratin, type II cytoskeletal 74 OS=Homo sapiens OX=9606 GN=KRT74 PE=1 SV=1	6	4	1	0	543	59.4	7.44
High	G3V180	Dipeptidyl peptidase 3 OS=Homo sapiens OX=9606 GN=DPP3 PE=1 SV=1	10	5	5	0	757	84.3	5.03
High	O43865	S-adenosylhomocysteine hydrolase-like protein 1 OS=Homo sapiens OX=9606 GN=AHCYL1 PE=1 SV=2	9	5	4	0	530	58.9	6.89
High	P21399	Cytoplasmic aconitate hydratase OS=Homo sapiens OX=9606 GN=ACO1 PE=1 SV=3	8	6	6	0	889	98.3	6.68
High	P18669	Phosphoglycerate mutase 1 OS=Homo sapiens OX=9606 GN=PGAM1 PE=1 SV=2	18	4	4	0	254	28.8	7.18
High	Q14203	Dynactin subunit 1 OS=Homo sapiens OX=9606 GN=DCTN1 PE=1 SV=3	4	5	5	0	1278	141.6	5.81
High	P36507	Dual specificity mitogen-activated protein kinase kinase 2 OS=Homo sapiens OX=9606 GN=MAP2K2 PE=1 SV=1	8	3	2	1	400	44.4	6.55
High	P48637	Glutathione synthetase OS=Homo sapiens OX=9606 GN=GSS PE=1 SV=1	14	7	7	0	474	52.4	5.92
High	E7EWC2	Ras GTPase-activating-like protein IQGAP2 (Fragment) OS=Homo sapiens OX=9606 GN=IQGAP2 PE=1 SV=1	3	5	5	0	1402	160.2	5.39
High	A0A087WU43	Cadherin-1 OS=Homo sapiens OX=9606 GN=CDH1 PE=1 SV=1	5	4	4	0	647	71.2	4.94
High	F8VV59	Nucleosome assembly protein 1-like 1 OS=Homo sapiens OX=9606 GN=NAP1L1 PE=1 SV=1	8	2	2	0	327	38	4.68
High	P31939	Bifunctional purine biosynthesis protein ATIC OS=Homo sapiens OX=9606 GN=ATIC PE=1 SV=3	11	5	5	0	592	64.6	6.71
High	A0A7I2V4Y9	Dynactin subunit 2 OS=Homo sapiens OX=9606 GN=DCTN2 PE=4 SV=1	12	4	4	0	378	41.6	5.91
High	G3V3E8	Epididymal secretory protein E1 OS=Homo sapiens OX=9606 GN=NPC2 PE=1 SV=1	20	2	2	0	174	19.2	8.44

High	P15121	Aldo-keto reductase family 1 member B1 OS=Homo sapiens OX=9606 GN=AKR1B1 PE=1 SV=3	10	4	3	0	316	35.8	6.98
High	P63000	Ras-related C3 botulinum toxin substrate 1 OS=Homo sapiens OX=9606 GN=RAC1 PE=1 SV=1	28	5	5	0	192	21.4	8.5
High	P14543	Nidogen-1 OS=Homo sapiens OX=9606 GN=NID1 PE=1 SV=3	6	6	6	0	1247	136.3	5.29
High	O43747	AP-1 complex subunit gamma-1 OS=Homo sapiens OX=9606 GN=AP1G1 PE=1 SV=5	6	5	5	0	822	91.3	6.8
High	P40763	Signal transducer and activator of transcription 3 OS=Homo sapiens OX=9606 GN=STAT3 PE=1 SV=2	4	3	3	0	770	88	6.3
High	A0A3B3IRX2	Phospholipase A2 OS=Homo sapiens OX=9606 GN=PLA2G2A PE=1 SV=1	36	5	5	0	117	13	9.06
High	Q8N1G4	Leucine-rich repeat-containing protein 47 OS=Homo sapiens OX=9606 GN=LRRC47 PE=1 SV=1	8	4	4	0	583	63.4	8.28
High	Q7KZF4	Staphylococcal nuclease domain-containing protein 1 OS=Homo sapiens OX=9606 GN=SND1 PE=1 SV=1	5	5	5	0	910	101.9	7.17
High	P42785	Lysosomal Pro-X carboxypeptidase OS=Homo sapiens OX=9606 GN=PRCP PE=1 SV=1	8	4	4	0	496	55.8	7.21
High	P55259	Pancreatic secretory granule membrane major glycoprotein GP2 OS=Homo sapiens OX=9606 GN=GP2 PE=1 SV=3	7	3	3	0	537	59.4	5.24
High	Q16651	Prostasin OS=Homo sapiens OX=9606 GN=PRSS8 PE=1 SV=1	10	2	2	0	343	36.4	5.85
High	P40926	Malate dehydrogenase, mitochondrial OS=Homo sapiens OX=9606 GN=MDH2 PE=1 SV=3	16	5	5	0	338	35.5	8.68
High	Q5T4F7	Secreted frizzled-related protein 5 OS=Homo sapiens OX=9606 GN=SFRP5 PE=2 SV=3	8	3	3	0	317	35.5	8.56
High	Q13557	Calcium/calmodulin-dependent protein kinase type II subunit delta OS=Homo sapiens OX=9606 GN=CAMK2D PE=1 SV=3	9	4	4	0	499	56.3	7.25
High	P84243	Histone H3.3 OS=Homo sapiens OX=9606 GN=H3-3A PE=1 SV=2	21	4	1	3	136	15.3	11.27
High	P25786	Proteasome subunit alpha type-1 OS=Homo sapiens OX=9606 GN=PSMA1 PE=1 SV=1	15	5	5	0	263	29.5	6.61
High	Q8TC12	Retinol dehydrogenase 11 OS=Homo sapiens OX=9606 GN=RDH11 PE=1 SV=2	11	3	3	0	318	35.4	8.82
High	P62191	26S proteasome regulatory subunit 4 OS=Homo sapiens OX=9606 GN=PSMC1 PE=1 SV=1	10	4	3	1	440	49.2	6.21
High	C9JK28	Disintegrin and metalloproteinase domain-containing protein 7 OS=Homo sapiens OX=9606 GN=ADAM7 PE=1 SV=1	6	4	4	0	776	88.1	6.49
High	O60763	General vesicular transport factor p115 OS=Homo sapiens OX=9606 GN=USO1 PE=1 SV=2	7	7	7	0	962	107.8	4.91
High	O95861	3'(2'),5'-bisphosphate nucleotidase 1 OS=Homo sapiens OX=9606 GN=BPNT1 PE=1 SV=1	12	4	4	0	308	33.4	5.69
High	P28482	Mitogen-activated protein kinase 1 OS=Homo sapiens OX=9606 GN=MAPK1 PE=1 SV=3	8	3	3	0	360	41.4	6.98
High	P02649	Apolipoprotein E OS=Homo sapiens OX=9606 GN=APOE PE=1 SV=1	11	3	3	0	317	36.1	5.73
High	P52907	F-actin-capping protein subunit alpha-1 OS=Homo sapiens OX=9606 GN=CAPZA1 PE=1 SV=3	20	4	3	0	286	32.9	5.69
High	A0A0A0MSX9	Isoleucyl-tRNA synthetase OS=Homo sapiens OX=9606 GN=IARS1 PE=1 SV=1	5	6	6	0	1242	142.2	6.09
High	P25788	Proteasome subunit alpha type-3 OS=Homo sapiens OX=9606 GN=PSMA3 PE=1 SV=2	16	4	4	0	255	28.4	5.33
High	Q9Y371	Endophilin-B1 OS=Homo sapiens OX=9606 GN=SH3GLB1 PE=1 SV=1	9	3	3	0	365	40.8	6.04
High	F8VWK8	Tetraspanin (Fragment) OS=Homo sapiens OX=9606 GN=CD63 PE=1 SV=1	13	4	4	0	217	23.4	7.68
High	P54289	Voltage-dependent calcium channel subunit alpha-2/delta-1 OS=Homo sapiens OX=9606 GN=CACNA2D1 PE=1 SV=3	4	4	4	0	1103	124.5	5.27
High	P35573	Glycogen debranching enzyme OS=Homo sapiens OX=9606 GN=AGL PE=1 SV=3	3	4	4	0	1532	174.7	6.76
High	K7EMH1	60S ribosomal protein L22 (Fragment) OS=Homo sapiens OX=9606 GN=RPL22 PE=1 SV=1	35	4	4	0	89	10.4	9.66
High	O94985	Calsyntenin-1 OS=Homo sapiens OX=9606 GN=CLSTN1 PE=1 SV=1	4	4	4	0	981	109.7	4.91
High	H0Y3U6	Glypican-3 (Fragment) OS=Homo sapiens OX=9606 GN=GPC3 PE=1 SV=1	8	2	2	0	256	29.1	6.86
High	J3KR44	Ubiquitin thioesterase OS=Homo sapiens OX=9606 GN=OTUB1 PE=1 SV=2	15	4	4	0	272	31.4	5
High	A0A2U3TZL5	CD59 glycoprotein (Fragment) OS=Homo sapiens OX=9606 GN=CD59 PE=1 SV=1	28	4	4	0	120	13.3	6.25
High	Q9UIK5	Tomoregulin-2 OS=Homo sapiens OX=9606 GN=TMEFF2 PE=1 SV=1	11	4	4	0	374	41.4	5.15
High	Q9H0E2	Toll-interacting protein OS=Homo sapiens OX=9606 GN=TOLLIP PE=1 SV=1	13	3	3	0	274	30.3	5.97
High	O00231	26S proteasome non-ATPase regulatory subunit 11 OS=Homo sapiens OX=9606 GN=PSMD11 PE=1 SV=3	5	2	2	0	422	47.4	6.48
High	A0A087WUI8	Mannose-1-phosphate guanyltransferase alpha OS=Homo sapiens OX=9606 GN=GMPPA PE=1 SV=1	9	3	3	0	399	43.6	7.17
High	A0A286YFJ8	Immunoglobulin heavy constant gamma 4 (Fragment) OS=Homo sapiens OX=9606 GN=IGHG4 PE=1 SV=1	11	4	2	0	396	43.8	6.24
High	P04040	Catalase OS=Homo sapiens OX=9606 GN=CAT PE=1 SV=3	7	3	3	0	527	59.7	7.39
High	P11169	Solute carrier family 2, facilitated glucose transporter member 3 OS=Homo sapiens OX=9606 GN=SLC2A3 PE=1 SV=1	4	2	2	0	496	53.9	7.2
High	A0A087WZQ6	AP-2 complex subunit beta (Fragment) OS=Homo sapiens OX=9606 GN=AP2B1 PE=1 SV=1	8	6	1	0	677	75.2	5.12
High	Q02252	Methylmalonate-semialdehyde dehydrogenase [acylating], mitochondrial OS=Homo sapiens OX=9606 GN=ALDH6A1 PE=1 SV=2	7	4	4	0	535	57.8	8.5
High	I3L4C2	Brain-specific angiogenesis inhibitor 1-associated protein 2 OS=Homo sapiens OX=9606 GN=BAIAP2 PE=1 SV=1	10	6	6	0	553	61.3	8.94
High	O95967	EGF-containing fibulin-like extracellular matrix protein 2 OS=Homo sapiens OX=9606 GN=EFEMP2 PE=1 SV=3	9	4	4	0	443	49.4	4.94
High	P07711	Procathepsin L OS=Homo sapiens OX=9606 GN=CTSL PE=1 SV=2	12	3	3	0	333	37.5	5.45
High	O00560	Syntenin-1 OS=Homo sapiens OX=9606 GN=SDCBP PE=1 SV=1	13	4	4	0	298	32.4	7.53
High	Q99460	26S proteasome non-ATPase regulatory subunit 1 OS=Homo sapiens OX=9606 GN=PSMD1 PE=1 SV=2	4	4	4	0	953	105.8	5.39
High	Q9Y646	Carboxypeptidase Q OS=Homo sapiens OX=9606 GN=CPQ PE=1 SV=1	6	3	3	0	472	51.9	6.18
High	E9PRY8	Elongation factor 1-delta OS=Homo sapiens OX=9606 GN=EEF1D PE=1 SV=1	8	4	1	3	697	76.5	7.05
High	P16930	Fumarylacetoacetase OS=Homo sapiens OX=9606 GN=FAH PE=1 SV=2	10	4	4	0	419	46.3	6.95
High	P13716	Delta-aminolevulinic acid dehydratase OS=Homo sapiens OX=9606 GN=ALAD PE=1 SV=1	12	3	3	0	330	36.3	6.79
High	Q6BCY4	NADH-cytochrome b5 reductase 2 OS=Homo sapiens OX=9606 GN=CYB5R2 PE=1 SV=1	13	4	4	0	276	31.4	8.5
High	K7ELL7	Glucosidase 2 subunit beta OS=Homo sapiens OX=9606 GN=PRKCSH PE=1 SV=1	7	4	4	0	535	60.2	4.41
High	P08865	40S ribosomal protein SA OS=Homo sapiens OX=9606 GN=RPSA PE=1 SV=4	10	2	2	0	295	32.8	4.87
High	P54826	Growth arrest-specific protein 1 OS=Homo sapiens OX=9606 GN=GAS1 PE=2 SV=2	8	3	3	0	345	35.7	5.55
High	Q9Y678	Coatomer subunit gamma-1 OS=Homo sapiens OX=9606 GN=COPG1 PE=1 SV=1	5	5	5	0	874	97.7	5.47
High	K7ES25	Xaa-Pro dipeptidase (Fragment) OS=Homo sapiens OX=9606 GN=PEPD PE=1 SV=3	7	4	4	0	512	56.5	6.25
High	P43686	26S proteasome regulatory subunit 6B OS=Homo sapiens OX=9606 GN=PSMC4 PE=1 SV=2	10	4	4	0	418	47.3	5.21
High	P13807	Glycogen [starch] synthase, muscle OS=Homo sapiens OX=9606 GN=GYS1 PE=1 SV=2	3	2	2	0	737	83.7	6.18
High	P61158	Actin-related protein 3 OS=Homo sapiens OX=9606 GN=ACTR3 PE=1 SV=3	9	3	3	0	418	47.3	5.88
High	Q9BVM2	Protein DPCD OS=Homo sapiens OX=9606 GN=DPCD PE=1 SV=2	17	3	3	0	203	23.2	9.03
High	P61006	Ras-related protein Rab-8A OS=Homo sapiens OX=9606 GN=RAB8A PE=1 SV=1	18	4	1	0	207	23.7	9.07
High	P49223	Kunitz-type protease inhibitor 3 OS=Homo sapiens OX=9606 GN=SPINT3 PE=3 SV=3	29	2	2	0	89	10.2	6.44
High	P48454	Serine/threonine-protein phosphatase 2B catalytic subunit gamma isoform OS=Homo sapiens OX=9606 GN=PPP3CC PE=1 SV=3	7	4	1	0	512	58.1	6.98
High	O15144	Actin-related protein 2/3 complex subunit 2 OS=Homo sapiens OX=9606 GN=ARPC2 PE=1 SV=1	12	4	4	0	300	34.3	7.36
High	Q15418	Ribosomal protein S6 kinase alpha-1 OS=Homo sapiens OX=9606 GN=RPS6KA1 PE=1 SV=2	4	3	3	0	735	82.7	7.83
High	Q14166	Tubulin--tyrosine ligase-like protein 12 OS=Homo sapiens OX=9606 GN=TTLL12 PE=1 SV=2	7	3	3	0	644	74.4	5.53

High	P00568	Adenylate kinase isoenzyme 1 OS=Homo sapiens OX=9606 GN=AK1 PE=1 SV=3	13	2	2	0	194	21.6	8.63
High	P51659	Peroxisomal multifunctional enzyme type 2 OS=Homo sapiens OX=9606 GN=HSD17B4 PE=1 SV=3	9	5	5	0	736	79.6	8.84
High	Q9NP72	Ras-related protein Rab-18 OS=Homo sapiens OX=9606 GN=RAB18 PE=1 SV=1	15	3	3	0	206	23	5.24
High	O00462	Beta-mannosidase OS=Homo sapiens OX=9606 GN=MANBA PE=1 SV=3	4	4	3	0	879	100.8	5.52
High	P04899	Guanine nucleotide-binding protein G(i) subunit alpha-2 OS=Homo sapiens OX=9606 GN=GNAI2 PE=1 SV=3	9	3	2	0	355	40.4	5.54
High	P08246	Neutrophil elastase OS=Homo sapiens OX=9606 GN=ELANE PE=1 SV=1	7	2	2	0	267	28.5	9.35
High	O94760	N(G),N(G)-dimethylarginine dimethylaminohydrolase 1 OS=Homo sapiens OX=9606 GN=DDAH1 PE=1 SV=3	20	4	4	0	285	31.1	5.81
High	Q13596	Sorting nexin-1 OS=Homo sapiens OX=9606 GN=SNX1 PE=1 SV=3	7	3	2	0	522	59	5.15
High	Q9NSD9	Phenylalanine--tRNA ligase beta subunit OS=Homo sapiens OX=9606 GN=FARSB PE=1 SV=3	7	4	4	0	589	66.1	6.84
High	P25787	Proteasome subunit alpha type-2 OS=Homo sapiens OX=9606 GN=PSMA2 PE=1 SV=2	10	2	2	0	234	25.9	7.43
High	F8W6H5	Ectonucleotide pyrophosphatase/phosphodiesterase family member 3 OS=Homo sapiens OX=9606 GN=ENPP3 PE=1 SV=1	9	5	5	0	663	76	7.03
High	P60953	Cell division control protein 42 homolog OS=Homo sapiens OX=9606 GN=CDC42 PE=1 SV=2	14	2	2	0	191	21.2	6.55
High	P53618	Coatomer subunit beta OS=Homo sapiens OX=9606 GN=COPB1 PE=1 SV=3	5	3	3	0	953	107.1	6.05
High	Q9UBS3	DnaJ homolog subfamily B member 9 OS=Homo sapiens OX=9606 GN=DNAJB9 PE=1 SV=1	14	2	2	0	223	25.5	8.27
High	Q07960	Rho GTPase-activating protein 1 OS=Homo sapiens OX=9606 GN=ARHGAP1 PE=1 SV=1	11	4	4	0	439	50.4	6.29
High	A0A087X2B5	Basigin (Fragment) OS=Homo sapiens OX=9606 GN=BSG PE=1 SV=1	15	3	3	0	221	23.9	5.25
High	A0A024RCR6	BCL2-associated athanogene 6 OS=Homo sapiens OX=9606 GN=BAG6 PE=1 SV=1	3	3	3	0	1126	118.6	5.6
High	P17858	ATP-dependent 6-phosphofructokinase, liver type OS=Homo sapiens OX=9606 GN=PFKL PE=1 SV=6	6	4	3	0	780	85	7.5
High	P62195	26S proteasome regulatory subunit 8 OS=Homo sapiens OX=9606 GN=PSMC5 PE=1 SV=1	13	4	3	0	406	45.6	7.55
High	B9A041	Malate dehydrogenase, cytoplasmic OS=Homo sapiens OX=9606 GN=MDH1 PE=1 SV=1	19	4	4	0	210	23	7.4
High	Q00577	Transcriptional activator protein Pur-alpha OS=Homo sapiens OX=9606 GN=PURA PE=1 SV=2	11	4	4	0	322	34.9	6.44
High	P07948	Tyrosine-protein kinase Lyn OS=Homo sapiens OX=9606 GN=LYN PE=1 SV=3	7	4	2	0	512	58.5	7.11
High	P50148	Guanine nucleotide-binding protein G(q) subunit alpha OS=Homo sapiens OX=9606 GN=GNAQ PE=1 SV=4	11	4	4	0	359	42.1	5.68
High	P62280	40S ribosomal protein S11 OS=Homo sapiens OX=9606 GN=RPS11 PE=1 SV=3	18	3	3	0	158	18.4	10.3
High	F6TLX2	Glyoxalase domain-containing protein 4 OS=Homo sapiens OX=9606 GN=GLOD4 PE=1 SV=1	5	2	2	0	502	54.7	8.7
High	E7EUC7	UTP--glucose-1-phosphate uridylyltransferase OS=Homo sapiens OX=9606 GN=UGP2 PE=1 SV=1	11	6	6	0	517	57.8	8.13
High	A0A087X2I1	26S proteasome regulatory subunit 10B OS=Homo sapiens OX=9606 GN=PSMC6 PE=1 SV=1	7	2	2	0	403	45.8	7.78
High	P20151	Kallikrein-2 OS=Homo sapiens OX=9606 GN=KLK2 PE=1 SV=1	15	3	2	0	261	28.7	6.92
High	P14174	Macrophage migration inhibitory factor OS=Homo sapiens OX=9606 GN=MIF PE=1 SV=4	18	2	2	0	115	12.5	7.88
High	Q9NRF8	CTP synthase 2 OS=Homo sapiens OX=9606 GN=CTPS2 PE=1 SV=1	6	3	3	0	586	65.6	6.9
High	Q9UNM6	26S proteasome non-ATPase regulatory subunit 13 OS=Homo sapiens OX=9606 GN=PSMD13 PE=1 SV=2	9	4	4	0	376	42.9	5.81
High	P26639	Threonine--tRNA ligase 1, cytoplasmic OS=Homo sapiens OX=9606 GN=TARS1 PE=1 SV=3	4	3	3	0	723	83.4	6.67
High	A0A6Q8PHB5	Cytosolic purine 5'-nucleotidase OS=Homo sapiens OX=9606 GN=NT5C2 PE=1 SV=1	9	4	4	0	436	50.1	7.23
High	A0A7I2V649	Polyadenylate-binding protein 1 OS=Homo sapiens OX=9606 GN=PABPC1 PE=4 SV=1	6	3	3	0	590	65.1	9.36
High	O00743	Serine/threonine-protein phosphatase 6 catalytic subunit OS=Homo sapiens OX=9606 GN=PPP6C PE=1 SV=1	9	2	2	0	305	35.1	5.69
High	Q9UJ70	N-acetyl-D-glucosamine kinase OS=Homo sapiens OX=9606 GN=NAGK PE=1 SV=4	10	3	3	0	344	37.4	6.24
High	P31431	Syndecan-4 OS=Homo sapiens OX=9606 GN=SDC4 PE=1 SV=2	11	2	2	0	198	21.6	4.5
High	J3KN67	Tropomyosin alpha-3 chain OS=Homo sapiens OX=9606 GN=TPM3 PE=1 SV=1	13	3	3	0	285	33.2	4.77
High	P68402	Platelet-activating factor acetylhydrolase IB subunit alpha2 OS=Homo sapiens OX=9606 GN=PAFAH1B2 PE=1 SV=1	12	2	2	0	229	25.6	5.92
High	Q86TX2	Acyl-coenzyme A thioesterase 1 OS=Homo sapiens OX=9606 GN=ACOT1 PE=1 SV=1	8	3	3	0	421	46.2	7.34
High	P40121	Macrophage-capping protein OS=Homo sapiens OX=9606 GN=CAPG PE=1 SV=2	7	2	2	0	348	38.5	6.19
High	P26572	Alpha-1,3-mannosyl-glycoprotein 2-beta-N-acetylglucosaminyltransferase OS=Homo sapiens OX=9606 GN=MGAT1 PE=1 SV=2	8	4	4	0	445	50.8	9.16
High	F5H6T1	ARP2 actin-related protein 2 homolog (Yeast), isoform CRA_d OS=Homo sapiens OX=9606 GN=ACTR2 PE=1 SV=2	8	2	2	0	339	38.8	6.27
High	Q8NBZ7	UDP-glucuronic acid decarboxylase 1 OS=Homo sapiens OX=9606 GN=UXS1 PE=1 SV=1	8	3	3	0	420	47.5	8.94
High	Q14515	SPARC-like protein 1 OS=Homo sapiens OX=9606 GN=SPARCL1 PE=1 SV=2	4	2	2	0	664	75.2	4.81
High	K7EN91	Bifunctional coenzyme A synthase (Fragment) OS=Homo sapiens OX=9606 GN=COASY PE=1 SV=1	15	3	3	0	219	23.7	8.48
High	P13667	Protein disulfide-isomerase A4 OS=Homo sapiens OX=9606 GN=PDIA4 PE=1 SV=2	5	3	3	0	645	72.9	5.07
High	Q8NEU8	DCC-interacting protein 13-beta OS=Homo sapiens OX=9606 GN=APPL2 PE=1 SV=3	5	4	4	0	664	74.4	4.94
High	D6RG13	40S ribosomal protein S3a (Fragment) OS=Homo sapiens OX=9606 GN=RPS3A PE=1 SV=1	12	3	3	0	223	25.6	9.66
High	B5MCT8	40S ribosomal protein S9 OS=Homo sapiens OX=9606 GN=RPS9 PE=1 SV=1	19	4	4	0	139	16.6	11.06
High	P05783	Keratin, type I cytoskeletal 18 OS=Homo sapiens OX=9606 GN=KRT18 PE=1 SV=2	8	4	2	0	430	48	5.45
High	P29692	Elongation factor 1-delta OS=Homo sapiens OX=9606 GN=EEF1D PE=1 SV=5	18	4	1	0	281	31.1	5.01
High	Q9BUD6	Spondin-2 OS=Homo sapiens OX=9606 GN=SPON2 PE=1 SV=3	5	3	3	0	331	35.8	5.52
High	Q9NRD9	Dual oxidase 1 OS=Homo sapiens OX=9606 GN=DUOX1 PE=1 SV=1	3	4	4	0	1551	177.1	7.9
High	C9IZZ0	Ras-related protein Rab-7a OS=Homo sapiens OX=9606 GN=RAB7A PE=1 SV=2	15	2	2	0	156	17.6	7.94
High	P19801	Amiloride-sensitive amine oxidase [copper-containing] OS=Homo sapiens OX=9606 GN=AOC1 PE=1 SV=4	4	3	3	0	751	85.3	7.09
High	P30085	UMP-CMP kinase OS=Homo sapiens OX=9606 GN=CMPK1 PE=1 SV=3	19	4	4	0	196	22.2	5.57
High	E7ETN3	C3/C5 convertase OS=Homo sapiens OX=9606 PE=1 SV=1	2	2	2	0	1115	123.9	6.8
High	O14818	Proteasome subunit alpha type-7 OS=Homo sapiens OX=9606 GN=PSMA7 PE=1 SV=1	14	3	3	0	248	27.9	8.46
High	P21980	Protein-glutamine gamma-glutamyltransferase 2 OS=Homo sapiens OX=9606 GN=TGM2 PE=1 SV=2	7	5	4	0	687	77.3	5.22
High	P15289	Arylsulfatase A OS=Homo sapiens OX=9606 GN=ARSA PE=1 SV=3	3	1	1	0	507	53.6	6.07
High	P36578	60S ribosomal protein L4 OS=Homo sapiens OX=9606 GN=RPL4 PE=1 SV=5	5	2	2	0	427	47.7	11.06
High	P62906	60S ribosomal protein L10a OS=Homo sapiens OX=9606 GN=RPL10A PE=1 SV=2	6	1	1	0	217	24.8	9.94
High	Q96CN7	Isochorismatase domain-containing protein 1 OS=Homo sapiens OX=9606 GN=ISOC1 PE=1 SV=3	9	2	2	0	298	32.2	7.39
High	Q8N8N7	Prostaglandin reductase 2 OS=Homo sapiens OX=9606 GN=PTGR2 PE=1 SV=1	8	3	3	0	351	38.5	5.41
High	Q9NZL9	Methionine adenosyltransferase 2 subunit beta OS=Homo sapiens OX=9606 GN=MAT2B PE=1 SV=1	11	3	3	0	334	37.5	7.36
High	O60841	Eukaryotic translation initiation factor 5B OS=Homo sapiens OX=9606 GN=EIF5B PE=1 SV=4	2	2	2	0	1220	138.7	5.49
High	P05388	60S acidic ribosomal protein P0 OS=Homo sapiens OX=9606 GN=RPLP0 PE=1 SV=1	7	2	2	0	317	34.3	5.97
High	O60513	Beta-1,4-galactosyltransferase 4 OS=Homo sapiens OX=9606 GN=B4GALT4 PE=1 SV=1	9	3	3	0	344	40	9.07
High	P56192	Methionine--tRNA ligase, cytoplasmic OS=Homo sapiens OX=9606 GN=MARS1 PE=1 SV=2	4	3	3	0	900	101.1	6.16

High	P20339	Ras-related protein Rab-5A OS=Homo sapiens OX=9606 GN=RAB5A PE=1 SV=2	13	3	1	1	215	23.6	8.15
High	Q9H1B5	Xylosyltransferase 2 OS=Homo sapiens OX=9606 GN=XYLT2 PE=1 SV=2	4	3	3	0	865	96.7	8.22
High	H0YCV6	Oxysterol-binding protein 1 (Fragment) OS=Homo sapiens OX=9606 GN=OSBP PE=1 SV=1	8	1	1	0	171	19.6	6.01
High	P51148	Ras-related protein Rab-5C OS=Homo sapiens OX=9606 GN=RAB5C PE=1 SV=2	15	3	1	0	216	23.5	8.41
High	P26640	Valine--tRNA ligase OS=Homo sapiens OX=9606 GN=VARS1 PE=1 SV=4	2	3	3	0	1264	140.4	7.59
High	Q9GZM7	Tubulointerstitial nephritis antigen-like OS=Homo sapiens OX=9606 GN=TINAGL1 PE=1 SV=1	8	3	3	0	467	52.4	6.99
High	P59665	Neutrophil defensin 1 OS=Homo sapiens OX=9606 GN=DEFA1 PE=1 SV=1	20	3	3	0	94	10.2	6.99
High	P01034	Cystatin-C OS=Homo sapiens OX=9606 GN=CST3 PE=1 SV=1	19	2	2	0	146	15.8	8.75
High	B5ME19	Eukaryotic translation initiation factor 3 subunit C-like protein OS=Homo sapiens OX=9606 GN=EIF3CL PE=1 SV=1	3	3	3	0	914	105.4	5.64
High	Q969X5	Endoplasmic reticulum-Golgi intermediate compartment protein 1 OS=Homo sapiens OX=9606 GN=ERGIC1 PE=1 SV=1	11	3	3	0	290	32.6	7.06
High	P59998	Actin-related protein 2/3 complex subunit 4 OS=Homo sapiens OX=9606 GN=ARPC4 PE=1 SV=3	17	3	3	0	168	19.7	8.43
High	P50570	Dynamin-2 OS=Homo sapiens OX=9606 GN=DNM2 PE=1 SV=2	4	4	4	0	870	98	7.44
High	Q00341	Vigilin OS=Homo sapiens OX=9606 GN=HDLBP PE=1 SV=2	3	3	3	0	1268	141.4	6.87
High	P15531	Nucleoside diphosphate kinase A OS=Homo sapiens OX=9606 GN=NME1 PE=1 SV=1	25	3	3	0	152	17.1	6.19
High	F5GYQ1	V-type proton ATPase subunit OS=Homo sapiens OX=9606 GN=ATP6V0D1 PE=1 SV=1	8	3	3	0	392	44.6	5.14
High	Q9H2G2	STE20-like serine/threonine-protein kinase OS=Homo sapiens OX=9606 GN=SLK PE=1 SV=1	2	4	4	0	1235	142.6	5.15
High	A0A7I2V5F8	Cathepsin F OS=Homo sapiens OX=9606 GN=CTSF PE=4 SV=1	8	3	3	0	407	44.8	7.24
High	C9J9S3	Serine/threonine-protein phosphatase (Fragment) OS=Homo sapiens OX=9606 GN=PPP1CB PE=1 SV=1	17	2	2	0	125	14.3	5.27
High	A8MUD9	60S ribosomal protein L7 OS=Homo sapiens OX=9606 GN=RPL7 PE=1 SV=1	10	2	2	0	208	24.4	10.67
High	Q3KQV9	UDP-N-acetylhexosamine pyrophosphorylase-like protein 1 OS=Homo sapiens OX=9606 GN=UAP1L1 PE=1 SV=2	7	4	4	0	507	57	6.32
High	A0A087WXM8	Basal cell adhesion molecule OS=Homo sapiens OX=9606 GN=BCAM PE=1 SV=1	4	2	2	0	588	63.7	6.1
High	P35080	Profilin-2 OS=Homo sapiens OX=9606 GN=PFN2 PE=1 SV=3	16	2	2	0	140	15	6.99
High	P98095	Fibulin-2 OS=Homo sapiens OX=9606 GN=FBLN2 PE=1 SV=2	3	2	2	0	1184	126.5	4.82
High	P46976	Glycogenin-1 OS=Homo sapiens OX=9606 GN=GYG1 PE=1 SV=4	8	3	3	0	350	39.4	5.53
High	J9JIE0	G-protein-coupled receptor family C group 5 member C OS=Homo sapiens OX=9606 GN=GPRC5C PE=1 SV=1	10	1	1	0	126	13.9	7.4
High	Q13275	Semaphorin-3F OS=Homo sapiens OX=9606 GN=SEMA3F PE=2 SV=2	4	3	3	0	785	88.3	8.27
High	P02763	Alpha-1-acid glycoprotein 1 OS=Homo sapiens OX=9606 GN=ORM1 PE=1 SV=1	7	1	1	0	201	23.5	5.02
High	A0A7I2V2D2	Plasma protease C1 inhibitor OS=Homo sapiens OX=9606 GN=SERPING1 PE=4 SV=1	6	3	3	0	482	53.2	6.47
High	H0YHA7	60S ribosomal protein L18 (Fragment) OS=Homo sapiens OX=9606 GN=RPL18 PE=1 SV=1	14	2	2	0	167	19	11.78
High	Q04760	Lactoylglutathione lyase OS=Homo sapiens OX=9606 GN=GLO1 PE=1 SV=4	17	5	5	0	184	20.8	5.31
High	P09467	Fructose-1,6-bisphosphatase 1 OS=Homo sapiens OX=9606 GN=FBP1 PE=1 SV=5	7	2	2	0	338	36.8	6.99
High	O00622	CCN family member 1 OS=Homo sapiens OX=9606 GN=CCN1 PE=1 SV=1	6	3	3	0	381	42	8.21
High	P30038	Delta-1-pyrroline-5-carboxylate dehydrogenase, mitochondrial OS=Homo sapiens OX=9606 GN=ALDH4A1 PE=1 SV=3	5	3	3	0	563	61.7	8.07
High	P15170	Eukaryotic peptide chain release factor GTP-binding subunit ERF3A OS=Homo sapiens OX=9606 GN=GSPT1 PE=1 SV=1	6	3	3	0	499	55.7	5.62
High	Q6NT32	Carboxylesterase 5A OS=Homo sapiens OX=9606 GN=CES5A PE=2 SV=1	7	3	3	0	575	63.9	6.44
High	Q9Y5Z0	Beta-secretase 2 OS=Homo sapiens OX=9606 GN=BACE2 PE=1 SV=1	4	2	2	0	518	56.1	5.15
High	A0A669KBF5	Cell cycle control protein OS=Homo sapiens OX=9606 GN=TMEM30A PE=1 SV=1	7	2	2	0	289	32.5	9.26
High	Q9BTY2	Plasma alpha-L-fucosidase OS=Homo sapiens OX=9606 GN=FUCA2 PE=1 SV=2	7	3	3	0	467	54	6.25
High	P17980	26S proteasome regulatory subunit 6A OS=Homo sapiens OX=9606 GN=PSMC3 PE=1 SV=3	6	2	2	0	439	49.2	5.24
High	B7Z3Z9	Long-chain-fatty-acid--CoA ligase 1 OS=Homo sapiens OX=9606 GN=ACSL1 PE=1 SV=1	6	3	3	0	527	58.5	7.53
High	Q9Y3I0	RNA-splicing ligase RtcB homolog OS=Homo sapiens OX=9606 GN=RTCB PE=1 SV=1	8	4	4	0	505	55.2	7.23
High	A0A0U1RR22	Protein kinase C and casein kinase substrate in neurons protein 2 (Fragment) OS=Homo sapiens OX=9606 GN=PACSIN2 PE=1 SV=1	2	1	1	0	451	51.8	5.34
High	K7ENC0	4-galactosyl-N-acetylglucosaminide 3-alpha-L-fucosyltransferase FUT5 OS=Homo sapiens OX=9606 GN=FUT5 PE=3 SV=1	9	2	2	0	374	43	8.12
High	Q99985	Semaphorin-3C OS=Homo sapiens OX=9606 GN=SEMA3C PE=2 SV=2	3	2	2	0	751	85.2	8.69
High	P15880	40S ribosomal protein S2 OS=Homo sapiens OX=9606 GN=RPS2 PE=1 SV=2	10	3	3	0	293	31.3	10.24
High	P48735	Isocitrate dehydrogenase [NADP], mitochondrial OS=Homo sapiens OX=9606 GN=IDH2 PE=1 SV=2	5	2	1	0	452	50.9	8.69
High	I3L3P7	40S ribosomal protein S15a OS=Homo sapiens OX=9606 GN=RPS15A PE=1 SV=1	19	2	2	0	100	11.5	10.15
High	H0Y8K0	Heat shock 70kDa protein 9B (Mortalin-2), isoform CRA_a OS=Homo sapiens OX=9606 GN=HSPA9 PE=1 SV=2	6	2	2	0	458	50	9.38
High	B8ZWD1	Acyl-CoA-binding protein OS=Homo sapiens OX=9606 GN=DBI PE=1 SV=1	11	2	2	0	97	11.1	8.32
High	Q8IXK2	Polypeptide N-acetylgalactosaminyltransferase 12 OS=Homo sapiens OX=9606 GN=GALNT12 PE=1 SV=3	4	2	2	0	581	66.9	6.8
High	P27105	Stomatin OS=Homo sapiens OX=9606 GN=STOM PE=1 SV=3	10	3	3	0	288	31.7	7.88
High	P20338	Ras-related protein Rab-4A OS=Homo sapiens OX=9606 GN=RAB4A PE=1 SV=3	10	2	1	0	218	24.4	6.07
High	B7Z7P8	Eukaryotic peptide chain release factor subunit 1 OS=Homo sapiens OX=9606 GN=ETF1 PE=1 SV=1	5	2	2	0	423	47.4	5.57
High	Q8NBX0	Saccharopine dehydrogenase-like oxidoreductase OS=Homo sapiens OX=9606 GN=SCCPDH PE=1 SV=1	8	3	3	0	429	47.1	9.14
High	Q15008	26S proteasome non-ATPase regulatory subunit 6 OS=Homo sapiens OX=9606 GN=PSMD6 PE=1 SV=1	4	2	2	0	389	45.5	5.62
High	Q7L1Q6	Basic leucine zipper and W2 domain-containing protein 1 OS=Homo sapiens OX=9606 GN=BZW1 PE=1 SV=1	7	3	3	0	419	48	5.92
High	Q5TH30	N-myc downstream-regulated gene 3 protein OS=Homo sapiens OX=9606 GN=NDRG3 PE=1 SV=1	7	2	2	0	388	42.8	5.33
High	D6RDX1	Vesicular integral-membrane protein VIP36 (Fragment) OS=Homo sapiens OX=9606 GN=LMAN2 PE=1 SV=1	7	2	2	0	288	32.7	6.14
High	O60749	Sorting nexin-2 OS=Homo sapiens OX=9606 GN=SNX2 PE=1 SV=2	4	2	1	0	519	58.4	5.12
High	Q6IS14	Eukaryotic translation initiation factor 5A-1-like OS=Homo sapiens OX=9606 GN=EIF5AL1 PE=2 SV=2	12	2	2	0	154	16.8	5
High	I3L1R7	Ethanolamine-phosphate cytidylyltransferase OS=Homo sapiens OX=9606 GN=PCYT2 PE=1 SV=1	8	3	3	0	367	41.4	7.36
High	I3L4J1	Vesicle-fusing ATPase (Fragment) OS=Homo sapiens OX=9606 PE=1 SV=3	6	3	2	1	428	48	7.53
High	Q5TEC6	Histone HIST2H3PS2 OS=Homo sapiens OX=9606 GN=H3-2 PE=1 SV=1	21	4	1	0	136	15.4	11.27
High	A0A0D9SF54	Spectrin alpha chain, non-erythrocytic 1 OS=Homo sapiens OX=9606 GN=SPTAN1 PE=1 SV=1	1	2	2	0	2457	282.7	5.34
High	A6PVX3	26S proteasome non-ATPase regulatory subunit 4 (Fragment) OS=Homo sapiens OX=9606 GN=PSMD4 PE=1 SV=1	13	2	2	0	203	21.8	7.11
High	A0A6I8PL42	Leucyl-tRNA synthetase OS=Homo sapiens OX=9606 GN=LARS1 PE=1 SV=1	3	3	3	0	1130	129.1	7.87
High	Q16563	Synaptophysin-like protein 1 OS=Homo sapiens OX=9606 GN=SYPL1 PE=1 SV=1	8	2	2	0	259	28.5	8.43
High	Q9HDB5	Neurexin-3-beta OS=Homo sapiens OX=9606 GN=NRXN3 PE=1 SV=4	4	2	2	0	637	69.3	7.21

High	H3BP35	Diphosphomevalonate decarboxylase (Fragment) OS=Homo sapiens OX=9606 GN=MVD PE=1 SV=1	5	1	1	0	271	29.4	8.72
High	Q06323	Proteasome activator complex subunit 1 OS=Homo sapiens OX=9606 GN=PSME1 PE=1 SV=1	10	3	3	0	249	28.7	6.02
High	Q8TBX8	Phosphatidylinositol 5-phosphate 4-kinase type-2 gamma OS=Homo sapiens OX=9606 GN=PIP4K2C PE=1 SV=3	8	3	3	0	421	47.3	6.84
High	Q02878	60S ribosomal protein L6 OS=Homo sapiens OX=9606 GN=RPL6 PE=1 SV=3	9	3	3	0	288	32.7	10.58
High	Q8NEB7	Acrosin-binding protein OS=Homo sapiens OX=9606 GN=ACRBP PE=2 SV=1	3	2	2	0	543	61.3	5.16
High	H0YDU8	Serine/threonine-protein phosphatase (Fragment) OS=Homo sapiens OX=9606 GN=PPP5C PE=1 SV=1	3	1	1	0	485	55.2	6.2
High	A0MZ66	Shootin-1 OS=Homo sapiens OX=9606 GN=SHTN1 PE=1 SV=4	4	2	2	0	631	71.6	5.33
High	P05109	Protein S100-A8 OS=Homo sapiens OX=9606 GN=S100A8 PE=1 SV=1	12	1	1	0	93	10.8	7.03
High	E7ETU9	Procollagen-lysine 5-dioxygenase OS=Homo sapiens OX=9606 GN=PLOD2 PE=1 SV=1	3	2	2	0	703	81.1	6.65
High	Q9UBQ7	Glyoxylate reductase/hydroxypyruvate reductase OS=Homo sapiens OX=9606 GN=GRHPR PE=1 SV=1	7	1	1	0	328	35.6	7.39
High	A0A0A6YYA0	Protein TMED7-TICAM2 OS=Homo sapiens OX=9606 GN=TMED7-TICAM2 PE=3 SV=1	5	1	1	0	188	21.2	6.2
High	P18065	Insulin-like growth factor-binding protein 2 OS=Homo sapiens OX=9606 GN=IGFBP2 PE=1 SV=2	6	2	2	0	325	34.8	7.5
High	O14841	5-oxoprolinase OS=Homo sapiens OX=9606 GN=OPLAH PE=1 SV=3	2	2	2	0	1288	137.4	6.58
High	P05198	Eukaryotic translation initiation factor 2 subunit 1 OS=Homo sapiens OX=9606 GN=EIF2S1 PE=1 SV=3	7	2	2	0	315	36.1	5.08
High	P51888	Prolargin OS=Homo sapiens OX=9606 GN=PRELP PE=1 SV=1	4	1	1	0	382	43.8	9.38
High	M0QZQ3	Spectrin beta chain OS=Homo sapiens OX=9606 GN=SPTBN4 PE=1 SV=1	1	2	1	0	2002	225.4	5.62
High	H7C1K4	Syndecan-1 (Fragment) OS=Homo sapiens OX=9606 GN=SDC1 PE=1 SV=1	8	1	1	0	215	22	4.6
High	U3KQT1	S-formylglutathione hydrolase OS=Homo sapiens OX=9606 GN=ESD PE=1 SV=2	9	2	2	0	262	29	7.33
High	P08118	Beta-microseminoprotein OS=Homo sapiens OX=9606 GN=MSMB PE=1 SV=1	13	3	3	0	114	12.9	5.5
High	P54920	Alpha-soluble NSF attachment protein OS=Homo sapiens OX=9606 GN=NAPA PE=1 SV=3	7	2	2	0	295	33.2	5.36
High	P43251	Biotinidase OS=Homo sapiens OX=9606 GN=BTD PE=1 SV=2	2	1	1	0	543	61.1	6.25
High	H0YHX9	Nascent polypeptide-associated complex subunit alpha OS=Homo sapiens OX=9606 GN=NACA PE=1 SV=2	6	1	1	0	235	25.3	4.55
High	Q92973	Transportin-1 OS=Homo sapiens OX=9606 GN=TNPO1 PE=1 SV=2	2	2	2	0	898	102.3	4.98
High	P35625	Metalloproteinase inhibitor 3 OS=Homo sapiens OX=9606 GN=TIMP3 PE=1 SV=2	9	2	2	0	211	24.1	8.72
High	A0A0A0MT67	Mucin-15 OS=Homo sapiens OX=9606 GN=MUC15 PE=1 SV=1	6	2	2	0	361	39.3	5.67
High	P08697	Alpha-2-antiplasmin OS=Homo sapiens OX=9606 GN=SERPINF2 PE=1 SV=3	8	2	2	0	491	54.5	6.29
High	P21796	Voltage-dependent anion-selective channel protein 1 OS=Homo sapiens OX=9606 GN=VDAC1 PE=1 SV=2	11	3	3	0	283	30.8	8.54
High	F2Z2K0	NSFL1 cofactor p47 OS=Homo sapiens OX=9606 GN=NSFL1C PE=1 SV=1	6	1	1	0	274	30.2	5.1
High	Q9H0W9	Ester hydrolase C11orf54 OS=Homo sapiens OX=9606 GN=C11orf54 PE=1 SV=1	7	2	2	0	315	35.1	6.7
High	F8VZX2	Poly(rC)-binding protein 2 OS=Homo sapiens OX=9606 GN=PCBP2 PE=1 SV=1	7	2	2	0	321	33.8	8.24
High	A0A7I2V3I2	Eukaryotic translation initiation factor 3 subunit E OS=Homo sapiens OX=9606 GN=EIF3E PE=4 SV=1	3	2	2	0	396	46.7	5.54
High	Q13630	GDP-L-fucose synthase OS=Homo sapiens OX=9606 GN=GFUS PE=1 SV=1	6	2	2	0	321	35.9	6.6
High	P13473	Lysosome-associated membrane glycoprotein 2 OS=Homo sapiens OX=9606 GN=LAMP2 PE=1 SV=2	4	2	2	0	410	44.9	5.63
High	E9PRA8	Alpha-crystallin B chain OS=Homo sapiens OX=9606 GN=CRYAB PE=1 SV=1	14	2	2	0	155	17.9	6.68
High	F8W809	Thioredoxin-disulfide reductase OS=Homo sapiens OX=9606 GN=TXNRD1 PE=1 SV=1	2	1	1	0	498	54.6	6.47
High	Q7L2H7	Eukaryotic translation initiation factor 3 subunit M OS=Homo sapiens OX=9606 GN=EIF3M PE=1 SV=1	5	2	2	0	374	42.5	5.63
High	A0A087WVT8	Kielin/chordin-like protein OS=Homo sapiens OX=9606 GN=KCP PE=1 SV=1	1	2	2	0	1628	173.5	5.88
High	H0YM70	Proteasome activator complex subunit 2 OS=Homo sapiens OX=9606 GN=PSME2 PE=1 SV=1	10	2	2	0	228	26	5.92
High	Q13724	Mannosyl-oligosaccharide glucosidase OS=Homo sapiens OX=9606 GN=MOGS PE=1 SV=5	2	2	2	0	837	91.9	8.9
High	Q9NRX4	14 kDa phosphohistidine phosphatase OS=Homo sapiens OX=9606 GN=PHPT1 PE=1 SV=1	17	1	1	0	125	13.8	6.07
High	P49755	Transmembrane emp24 domain-containing protein 10 OS=Homo sapiens OX=9606 GN=TMED10 PE=1 SV=2	9	2	2	0	219	25	7.44
High	P21741	Midkine OS=Homo sapiens OX=9606 GN=MDK PE=1 SV=1	17	3	3	0	143	15.6	9.79
High	D6RH31	Nephronectin (Fragment) OS=Homo sapiens OX=9606 GN=NPNT PE=1 SV=1	3	2	2	0	609	66.9	8.4
High	Q9Y6E0	Serine/threonine-protein kinase 24 OS=Homo sapiens OX=9606 GN=STK24 PE=1 SV=1	5	2	2	0	443	49.3	5.69
High	Q8TB96	T-cell immunomodulatory protein OS=Homo sapiens OX=9606 GN=ITFG1 PE=1 SV=1	5	2	2	0	612	68.1	5.39
High	A0A087WZG8	Lipase member I OS=Homo sapiens OX=9606 GN=LIPI PE=1 SV=1	3	1	1	0	421	48.6	9.03
High	Q08257	Quinone oxidoreductase OS=Homo sapiens OX=9606 GN=CRYZ PE=1 SV=1	6	2	2	0	329	35.2	8.44
High	J3KP15	Serine/arginine-rich splicing factor 2 (Fragment) OS=Homo sapiens OX=9606 GN=SRSF2 PE=1 SV=8	14	2	2	0	133	15.4	10.95
High	Q9Y5P6	Mannose-1-phosphate guanyltransferase beta OS=Homo sapiens OX=9606 GN=GMPPB PE=1 SV=2	9	3	3	0	360	39.8	6.61
High	Q86X29	Lipolysis-stimulated lipoprotein receptor OS=Homo sapiens OX=9606 GN=LSR PE=1 SV=4	3	2	2	0	649	71.4	7.97
High	P36959	GMP reductase 1 OS=Homo sapiens OX=9606 GN=GMPR PE=1 SV=1	6	2	2	0	345	37.4	7.06
High	Q08722	Leukocyte surface antigen CD47 OS=Homo sapiens OX=9606 GN=CD47 PE=1 SV=1	9	3	3	0	323	35.2	7.21
High	Q8NCH0	Carbohydrate sulfotransferase 14 OS=Homo sapiens OX=9606 GN=CHST14 PE=1 SV=2	3	1	1	0	376	43	9.48
High	A0A2R8YDT1	Glutamine synthetase OS=Homo sapiens OX=9606 GN=GLUL PE=1 SV=1	5	3	3	0	507	57.1	8.37
High	F5H8H3	START domain-containing protein 10 OS=Homo sapiens OX=9606 GN=STARD10 PE=1 SV=1	12	3	3	0	262	29.7	6.54
High	Q9Y3F4	Serine-threonine kinase receptor-associated protein OS=Homo sapiens OX=9606 GN=STRAP PE=1 SV=1	7	2	2	0	350	38.4	5.12
High	P51178	1-phosphatidylinositol 4,5-bisphosphate phosphodiesterase delta-1 OS=Homo sapiens OX=9606 GN=PLCD1 PE=1 SV=2	1	1	1	0	756	85.6	6.7
High	P09466	Glycodelin OS=Homo sapiens OX=9606 GN=PAEP PE=1 SV=2	8	1	1	0	180	20.6	5.57
High	Q6P4A8	Phospholipase B-like 1 OS=Homo sapiens OX=9606 GN=PLBD1 PE=1 SV=2	2	1	1	0	553	63.2	9.06
High	A0A087WVV1	Proteasome subunit beta type-2 OS=Homo sapiens OX=9606 GN=PSMB2 PE=1 SV=1	23	2	2	0	84	9.6	8.78
High	A0A7I2YQN2	Heterogeneous nuclear ribonucleoprotein Q OS=Homo sapiens OX=9606 GN=SYNCRIP PE=4 SV=1	4	2	2	0	561	62.5	7.56
High	K7ERP4	Glutathione peroxidase (Fragment) OS=Homo sapiens OX=9606 GN=GPX4 PE=1 SV=1	9	1	1	0	155	17.6	7.71
High	P35270	Sepiapterin reductase OS=Homo sapiens OX=9606 GN=SPR PE=1 SV=1	6	1	1	0	261	28	8.05
High	P49411	Elongation factor Tu, mitochondrial OS=Homo sapiens OX=9606 GN=TUFM PE=1 SV=2	4	2	2	0	452	49.5	7.61
High	O00442	RNA 3'-terminal phosphate cyclase OS=Homo sapiens OX=9606 GN=RTCA PE=1 SV=1	5	2	2	0	366	39.3	7.85
High	O43490	Prominin-1 OS=Homo sapiens OX=9606 GN=PROM1 PE=1 SV=1	2	1	1	0	865	97.1	7.27
High	P04632	Calpain small subunit 1 OS=Homo sapiens OX=9606 GN=CAPNS1 PE=1 SV=1	8	2	2	0	268	28.3	5.2
High	Q9NS25	Sperm protein associated with the nucleus on the X chromosome B1 OS=Homo sapiens OX=9606 GN=SPANXB1 PE=1 SV=2	11	1	1	0	103	11.8	6.15
High	P02792	Ferritin light chain OS=Homo sapiens OX=9606 GN=FTL PE=1 SV=2	9	1	1	0	175	20	5.78
High	H0YE35	Synembryn-A (Fragment) OS=Homo sapiens OX=9606 GN=RIC8A PE=1 SV=1	10	1	1	0	147	16.2	4.61
High	Q92900	Regulator of nonsense transcripts 1 OS=Homo sapiens OX=9606 GN=UPF1 PE=1 SV=2	2	2	2	0	1129	124.3	6.61
High	P50591	Tumor necrosis factor ligand superfamily member 10 OS=Homo sapiens OX=9606 GN=TNFSF10 PE=1 SV=1	4	1	1	0	281	32.5	7.42

High	O95433	Activator of 90 kDa heat shock protein ATPase homolog 1 OS=Homo sapiens OX=9606 GN=AHSA1 PE=1 SV=1	6	2	2	0	338	38.3	5.53
High	P28072	Proteasome subunit beta type-6 OS=Homo sapiens OX=9606 GN=PSMB6 PE=1 SV=4	9	2	2	0	239	25.3	4.92
High	P10155	60 kDa SS-A/Ro ribonucleoprotein OS=Homo sapiens OX=9606 GN=RO60 PE=1 SV=2	2	1	1	0	538	60.6	8.03
High	Q15102	Platelet-activating factor acetylhydrolase IB subunit alpha1 OS=Homo sapiens OX=9606 GN=PAFAH1B3 PE=1 SV=1	8	2	2	0	231	25.7	6.84
High	O95336	6-phosphogluconolactonase OS=Homo sapiens OX=9606 GN=PGLS PE=1 SV=2	9	2	2	0	258	27.5	6.05
High	P61204	ADP-ribosylation factor 3 OS=Homo sapiens OX=9606 GN=ARF3 PE=1 SV=2	10	2	1	1	181	20.6	7.43
High	P08311	Cathepsin G OS=Homo sapiens OX=9606 GN=CTSG PE=1 SV=2	7	2	2	0	255	28.8	11.19
High	P18085	ADP-ribosylation factor 4 OS=Homo sapiens OX=9606 GN=ARF4 PE=1 SV=3	10	2	1	0	180	20.5	7.14
High	A0A087X0K1	Calcium-binding protein 39 OS=Homo sapiens OX=9606 GN=CAB39 PE=1 SV=1	5	2	2	0	339	39.4	7.11
High	H3BND8	Ubiquitin carboxyl-terminal hydrolase (Fragment) OS=Homo sapiens OX=9606 GN=USP7 PE=1 SV=1	3	3	3	0	909	105.5	5.58
High	F8VXU5	Vacuolar protein sorting-associated protein 29 OS=Homo sapiens OX=9606 GN=VPS29 PE=1 SV=1	5	1	1	0	214	24	8.18
High	Q9NTK5	Obg-like ATPase 1 OS=Homo sapiens OX=9606 GN=OLA1 PE=1 SV=2	3	1	1	0	396	44.7	7.81
High	A0A0J9YW33	Syntaxin-3 OS=Homo sapiens OX=9606 GN=STX3 PE=1 SV=1	6	2	2	0	287	32.9	5.45
High	B7Z6Z4	Myosin light polypeptide 6 OS=Homo sapiens OX=9606 GN=MYL6 PE=1 SV=1	9	2	2	0	238	26.7	5.08
High	Q8IYS1	Peptidase M20 domain-containing protein 2 OS=Homo sapiens OX=9606 GN=PM20D2 PE=1 SV=2	3	1	1	0	436	47.7	5.85
High	Q9HAB8	Phosphopantothenate--cysteine ligase OS=Homo sapiens OX=9606 GN=PPCS PE=1 SV=2	5	1	1	0	311	34	6.71
High	K7EMS3	Keratin, type I cytoskeletal 19 (Fragment) OS=Homo sapiens OX=9606 GN=KRT19 PE=1 SV=1	12	2	1	0	181	20.2	4.6
High	O94886	CSC1-like protein 1 OS=Homo sapiens OX=9606 GN=TMEM63A PE=1 SV=3	1	1	1	0	807	92.1	7.27
High	P00918	Carbonic anhydrase 2 OS=Homo sapiens OX=9606 GN=CA2 PE=1 SV=2	7	2	2	0	260	29.2	7.4
High	O60243	Heparan-sulfate 6-O-sulfotransferase 1 OS=Homo sapiens OX=9606 GN=HS6ST1 PE=1 SV=5	4	2	2	0	411	48.2	8.79
High	Q02952	A-kinase anchor protein 12 OS=Homo sapiens OX=9606 GN=AKAP12 PE=1 SV=4	1	1	1	0	1782	191.4	4.41
High	M0QX76	40S ribosomal protein S16 (Fragment) OS=Homo sapiens OX=9606 GN=RPS16 PE=1 SV=1	20	1	1	0	50	5.6	9.63
High	C9JIG9	Non-specific serine/threonine protein kinase OS=Homo sapiens OX=9606 GN=OXSR1 PE=1 SV=1	3	1	1	0	468	51.8	7.8
High	Q8N3R9	MAGUK p55 subfamily member 5 OS=Homo sapiens OX=9606 GN=MPP5 PE=1 SV=3	3	2	2	0	675	77.2	6.14
High	P43034	Platelet-activating factor acetylhydrolase IB subunit beta OS=Homo sapiens OX=9606 GN=PAFAH1B1 PE=1 SV=2	7	3	2	0	410	46.6	7.37
High	P00492	Hypoxanthine-guanine phosphoribosyltransferase OS=Homo sapiens OX=9606 GN=HPRT1 PE=1 SV=2	6	1	1	0	218	24.6	6.68
High	A0A087WZK9	Eukaryotic translation initiation factor 3 subunit H OS=Homo sapiens OX=9606 GN=EIF3H PE=1 SV=1	6	1	1	0	349	39.6	6.39
High	Q9Y696	Chloride intracellular channel protein 4 OS=Homo sapiens OX=9606 GN=CLIC4 PE=1 SV=4	6	1	1	0	253	28.8	5.59
High	Q15365	Poly(rC)-binding protein 1 OS=Homo sapiens OX=9606 GN=PCBP1 PE=1 SV=2	8	2	2	0	356	37.5	7.09
High	Q00266	S-adenosylmethionine synthase isoform type-1 OS=Homo sapiens OX=9606 GN=MAT1A PE=1 SV=2	5	2	1	0	395	43.6	6.29
High	A0A3B3IRL5	Inosine-5'-monophosphate dehydrogenase (Fragment) OS=Homo sapiens OX=9606 GN=IMPDH1 PE=1 SV=1	4	2	1	0	509	54.3	6.8
High	P31930	Cytochrome b-c1 complex subunit 1, mitochondrial OS=Homo sapiens OX=9606 GN=UQCRC1 PE=1 SV=3	5	2	2	0	480	52.6	6.37
High	O95631	Netrin-1 OS=Homo sapiens OX=9606 GN=NTN1 PE=1 SV=2	3	1	1	0	604	67.7	8.76
High	E9PRJ8	Tetraspanin (Fragment) OS=Homo sapiens OX=9606 GN=CD81 PE=1 SV=1	19	2	2	0	209	22.5	6.15
High	F6WIT2	Serine/threonine-protein phosphatase 2A activator OS=Homo sapiens OX=9606 GN=PTPA PE=1 SV=1	8	2	2	0	288	32.9	6.93
High	O75351	Vacuolar protein sorting-associated protein 4B OS=Homo sapiens OX=9606 GN=VPS4B PE=1 SV=2	5	2	1	0	444	49.3	7.23
High	Q9UL25	Ras-related protein Rab-21 OS=Homo sapiens OX=9606 GN=RAB21 PE=1 SV=3	4	1	1	0	225	24.3	7.94
High	A0A140T974	Flotillin (Fragment) OS=Homo sapiens OX=9606 GN=FLOT1 PE=1 SV=1	12	1	1	0	90	9.6	6
High	P35443	Thrombospondin-4 OS=Homo sapiens OX=9606 GN=THBS4 PE=1 SV=2	2	1	1	0	961	105.8	4.68
High	Q99538	Legumain OS=Homo sapiens OX=9606 GN=LGMN PE=1 SV=1	6	2	2	0	433	49.4	6.55
High	P20618	Proteasome subunit beta type-1 OS=Homo sapiens OX=9606 GN=PSMB1 PE=1 SV=2	4	1	1	0	241	26.5	8.13
High	H0YAC6	Lipopolysaccharide-responsive and beige-like anchor protein (Fragment) OS=Homo sapiens OX=9606 GN=LRBA PE=1 SV=1	1	1	1	0	1505	166.8	5.64
High	P54577	Tyrosine--tRNA ligase, cytoplasmic OS=Homo sapiens OX=9606 GN=YARS1 PE=1 SV=4	5	3	3	0	528	59.1	7.05
High	P30044	Peroxiredoxin-5, mitochondrial OS=Homo sapiens OX=9606 GN=PRDX5 PE=1 SV=4	12	2	2	0	214	22.1	8.7
High	P62714	Serine/threonine-protein phosphatase 2A catalytic subunit beta isoform OS=Homo sapiens OX=9606 GN=PPP2CB PE=1 SV=1	7	2	2	0	309	35.6	5.43
High	Q96DG6	Carboxymethylenebutenolidase homolog OS=Homo sapiens OX=9606 GN=CMBL PE=1 SV=1	9	2	2	0	245	28	7.18
High	Q9Y6G9	Cytoplasmic dynein 1 light intermediate chain 1 OS=Homo sapiens OX=9606 GN=DYNC1LI1 PE=1 SV=3	4	2	2	0	523	56.5	6.42
High	Q13421	Mesothelin OS=Homo sapiens OX=9606 GN=MSLN PE=1 SV=2	3	2	2	0	630	68.9	6.38
High	P26373	60S ribosomal protein L13 OS=Homo sapiens OX=9606 GN=RPL13 PE=1 SV=4	9	2	2	0	211	24.2	11.65
High	P41091	Eukaryotic translation initiation factor 2 subunit 3 OS=Homo sapiens OX=9606 GN=EIF2S3 PE=1 SV=3	2	1	1	0	472	51.1	8.4
High	C9JD32	60S ribosomal protein L23 (Fragment) OS=Homo sapiens OX=9606 GN=RPL23 PE=1 SV=1	20	2	2	0	91	9.7	11.49
High	P39023	60S ribosomal protein L3 OS=Homo sapiens OX=9606 GN=RPL3 PE=1 SV=2	5	2	2	0	403	46.1	10.18
High	Q5W0H4	Translationally-controlled tumor protein OS=Homo sapiens OX=9606 GN=TPT1 PE=1 SV=1	9	2	2	0	188	21.5	5.49
High	Q93100	Phosphorylase b kinase regulatory subunit beta OS=Homo sapiens OX=9606 GN=PHKB PE=1 SV=3	2	2	2	0	1093	124.8	6.95
High	A0A087WVC6	Protein-tyrosine-phosphatase OS=Homo sapiens OX=9606 GN=PTPRJ PE=1 SV=1	2	2	2	0	1342	146.5	5.62
High	P45877	Peptidyl-prolyl cis-trans isomerase C OS=Homo sapiens OX=9606 GN=PPIC PE=1 SV=1	10	3	3	0	212	22.7	8.4
High	Q05655	Protein kinase C delta type OS=Homo sapiens OX=9606 GN=PRKCD PE=1 SV=2	3	2	2	0	676	77.5	7.75
High	Q8NBF2	NHL repeat-containing protein 2 OS=Homo sapiens OX=9606 GN=NHLRC2 PE=1 SV=1	3	2	2	0	726	79.4	5.55
High	O95460	Matrilin-4 OS=Homo sapiens OX=9606 GN=MATN4 PE=1 SV=3	4	2	2	0	622	68.4	6.1
High	Q6PGP7	Tetratricopeptide repeat protein 37 OS=Homo sapiens OX=9606 GN=TTC37 PE=1 SV=1	1	1	1	0	1564	175.4	7.53
High	H0YJ56	Protein NDRG2 (Fragment) OS=Homo sapiens OX=9606 GN=NDRG2 PE=1 SV=1	11	1	1	0	139	15.4	5.36
High	P78542	Hexokinase 1 (Fragment) OS=Homo sapiens OX=9606 GN=HK1 PE=1 SV=1	33	1	1	0	30	3.4	5.83
High	O14745	Na(+)/H(+) exchange regulatory cofactor NHE-RF1 OS=Homo sapiens OX=9606 GN=SLC9A3R1 PE=1 SV=4	7	2	2	0	358	38.8	5.77
High	D6RF35	Gc-globulin OS=Homo sapiens OX=9606 GN=GC PE=1 SV=1	4	2	2	0	476	53	5.52
High	P07585	Decorin OS=Homo sapiens OX=9606 GN=DCN PE=1 SV=1	5	2	1	0	359	39.7	8.54
High	E7EWE1	UFM1-activating enzyme OS=Homo sapiens OX=9606 GN=UBA5 PE=1 SV=1	3	1	1	0	347	38.7	5.47
High	P01116	GTPase KRas OS=Homo sapiens OX=9606 GN=KRAS PE=1 SV=1	6	1	1	0	189	21.6	6.77
High	G5E9D8	Inter-alpha (Globulin) inhibitor H5, isoform CRA_e OS=Homo sapiens OX=9606 GN=ITIH5 PE=1 SV=1	3	2	2	0	702	78.4	9.01
High	J3KSD8	Bleomycin hydrolase (Fragment) OS=Homo sapiens OX=9606 GN=BLMH PE=1 SV=8	4	1	1	0	262	30.2	7.46
High	Q8IV36	Protein HID1 OS=Homo sapiens OX=9606 GN=HID1 PE=1 SV=1	2	2	2	0	788	88.7	6.06
High	P49908	Selenoprotein P OS=Homo sapiens OX=9606 GN=SELENOP PE=1 SV=3	3	1	1	0	381	43.2	7.87
High	P00505	Aspartate aminotransferase, mitochondrial OS=Homo sapiens OX=9606 GN=GOT2 PE=1 SV=3	2	1	1	0	430	47.5	9.01

High	E9PLX7	60S ribosomal protein L27a OS=Homo sapiens OX=9606 GN=RPL27A PE=1 SV=1	10	1	1	0	113	12.5	11.44
High	P30740	Leukocyte elastase inhibitor OS=Homo sapiens OX=9606 GN=SERPINB1 PE=1 SV=1	3	1	1	0	379	42.7	6.28
High	H0YDT6	Eukaryotic translation initiation factor 3 subunit F OS=Homo sapiens OX=9606 GN=EIF3F PE=1 SV=2	3	1	1	0	359	37.7	6.06
High	J3QKW7	Developmentally-regulated GTP-binding protein 2 OS=Homo sapiens OX=9606 GN=DRG2 PE=1 SV=1	4	1	1	0	244	26.6	9.5
High	A0A1B0GW23	N-acyl-L-amino-acid amidohydrolase (Fragment) OS=Homo sapiens OX=9606 GN=ABHD14A-ACY1 PE=3 SV=1	2	1	1	0	586	65.4	6.67
High	O00203	AP-3 complex subunit beta-1 OS=Homo sapiens OX=9606 GN=AP3B1 PE=1 SV=3	1	1	1	0	1094	121.2	6.04
High	C9JQB3	Small monomeric GTPase (Fragment) OS=Homo sapiens OX=9606 GN=RALB PE=1 SV=1	6	1	1	0	171	19.2	5
High	Q14764	Major vault protein OS=Homo sapiens OX=9606 GN=MVP PE=1 SV=4	2	2	2	0	893	99.3	5.48
High	B1AHF3	NADH-cytochrome b5 reductase 3 (Fragment) OS=Homo sapiens OX=9606 GN=CYB5R3 PE=1 SV=1	7	1	1	0	147	16.7	9.73
High	P08263	Glutathione S-transferase A1 OS=Homo sapiens OX=9606 GN=GSTA1 PE=1 SV=3	7	2	2	0	222	25.6	8.88
High	A0A2R8YFQ7	Lambda-crystallin homolog OS=Homo sapiens OX=9606 GN=CRYL1 PE=1 SV=1	7	2	2	0	229	25.3	7.31
High	Q15147	1-phosphatidylinositol 4,5-bisphosphate phosphodiesterase beta-4 OS=Homo sapiens OX=9606 GN=PLCB4 PE=1 SV=3	1	1	1	0	1175	134.4	6.9
High	K7EN36	V-type proton ATPase subunit a (Fragment) OS=Homo sapiens OX=9606 GN=ATP6V0A1 PE=1 SV=1	5	1	1	0	195	22.4	4.86
High	P31689	DnaJ homolog subfamily A member 1 OS=Homo sapiens OX=9606 GN=DNAJA1 PE=1 SV=2	3	1	1	0	397	44.8	7.08
High	O95833	Chloride intracellular channel protein 3 OS=Homo sapiens OX=9606 GN=CLIC3 PE=1 SV=2	5	1	1	0	236	26.6	6.43
High	Q96G03	Phosphoglucomutase-2 OS=Homo sapiens OX=9606 GN=PGM2 PE=1 SV=4	4	2	2	0	612	68.2	6.73
High	B4DIW9	Serine/threonine-protein phosphatase 6 regulatory ankyrin repeat subunit A OS=Homo sapiens OX=9606 GN=ANKRD28 PE=1 SV=1	1	1	1	0	722	77.7	6.99
High	Q5GLZ8	Probable E3 ubiquitin-protein ligase HERC4 OS=Homo sapiens OX=9606 GN=HERC4 PE=1 SV=1	1	1	1	0	1057	118.5	6.19
High	O14950	Myosin regulatory light chain 12B OS=Homo sapiens OX=9606 GN=MYL12B PE=1 SV=2	6	1	1	0	172	19.8	4.84
High	Q13564	NEDD8-activating enzyme E1 regulatory subunit OS=Homo sapiens OX=9606 GN=NAE1 PE=1 SV=1	2	1	1	0	534	60.2	5.4
High	O00571	ATP-dependent RNA helicase DDX3X OS=Homo sapiens OX=9606 GN=DDX3X PE=1 SV=3	2	1	1	0	662	73.2	7.18
High	Q15904	V-type proton ATPase subunit S1 OS=Homo sapiens OX=9606 GN=ATP6AP1 PE=1 SV=2	3	2	2	0	470	52	6.14
High	O43681	ATPase GET3 OS=Homo sapiens OX=9606 GN=GET3 PE=1 SV=2	5	2	2	0	348	38.8	4.91
High	O43581	Synaptotagmin-7 OS=Homo sapiens OX=9606 GN=SYT7 PE=1 SV=3	5	2	2	0	403	45.5	9.22
High	F5GZQ3	Trifunctional enzyme subunit beta, mitochondrial OS=Homo sapiens OX=9606 GN=HADHB PE=1 SV=1	2	1	1	0	459	49.6	9.42
High	P0DOY2	Immunoglobulin lambda constant 2 OS=Homo sapiens OX=9606 GN=IGLC2 PE=1 SV=1	9	1	1	0	106	11.3	7.24
High	Q96HC4	PDZ and LIM domain protein 5 OS=Homo sapiens OX=9606 GN=PDLIM5 PE=1 SV=5	3	2	2	0	596	63.9	8.21
High	A0A7I2YQF8	Eukaryotic translation initiation factor 3 subunit I OS=Homo sapiens OX=9606 GN=EIF3I PE=4 SV=1	3	1	1	0	362	40.5	5.64
High	F5GX71	Mannose-6-phosphate isomerase OS=Homo sapiens OX=9606 GN=MPI PE=1 SV=1	3	1	1	0	373	41.2	6.09
High	P48444	Coatomer subunit delta OS=Homo sapiens OX=9606 GN=ARCN1 PE=1 SV=1	5	2	2	0	511	57.2	6.21
High	O43653	Prostate stem cell antigen OS=Homo sapiens OX=9606 GN=PSCA PE=1 SV=2	9	1	1	0	114	12	4.94
High	O75503	Ceroid-lipofuscinosis neuronal protein 5 OS=Homo sapiens OX=9606 GN=CLN5 PE=1 SV=2	3	1	1	0	358	41.5	7.4
High	H7BXG7	Protein transport protein Sec31A OS=Homo sapiens OX=9606 GN=SEC31A PE=1 SV=1	1	1	1	0	969	105.6	8.37
High	P37837	Transaldolase OS=Homo sapiens OX=9606 GN=TALDO1 PE=1 SV=2	5	2	2	0	337	37.5	6.81
High	A0A6Q8PHE5	Ubiquitin-protein ligase E3A OS=Homo sapiens OX=9606 GN=UBE3A PE=1 SV=1	1	1	1	0	813	93.5	5.14
High	Q9NZ08	Endoplasmic reticulum aminopeptidase 1 OS=Homo sapiens OX=9606 GN=ERAP1 PE=1 SV=3	1	1	1	0	941	107.2	6.46
High	Q96FJ2	Dynein light chain 2, cytoplasmic OS=Homo sapiens OX=9606 GN=DYNLL2 PE=1 SV=1	15	1	1	0	89	10.3	7.37
High	O14744	Protein arginine N-methyltransferase 5 OS=Homo sapiens OX=9606 GN=PRMT5 PE=1 SV=4	3	2	2	0	637	72.6	6.29
High	A0A286YFF8	Protein MON2 homolog OS=Homo sapiens OX=9606 GN=MON2 PE=1 SV=1	1	2	2	0	1718	190.4	6.1
High	Q9BV20	Methylthioribose-1-phosphate isomerase OS=Homo sapiens OX=9606 GN=MRI1 PE=1 SV=1	3	1	1	0	369	39.1	6.3
High	Q9P2R3	Rabankyrin-5 OS=Homo sapiens OX=9606 GN=ANKFY1 PE=1 SV=2	1	1	1	0	1169	128.3	6.1
High	P09525	Annexin A4 OS=Homo sapiens OX=9606 GN=ANXA4 PE=1 SV=4	3	1	1	0	319	35.9	6.13
High	P61978	Heterogeneous nuclear ribonucleoprotein K OS=Homo sapiens OX=9606 GN=HNRNPK PE=1 SV=1	2	1	1	0	463	50.9	5.54
High	A0A087WTB8	Ubiquitin carboxyl-terminal hydrolase OS=Homo sapiens OX=9606 GN=UCHL3 PE=1 SV=1	5	1	1	0	194	21.9	4.93
High	A0A7I2V5H3	Interferon-inducible double-stranded RNA-dependent protein kinase activator A OS=Homo sapiens OX=9606 GN=PRKRA PE=4 SV=1	5	1	1	0	191	21.5	8.09
High	H0YD57	CD82 antigen (Fragment) OS=Homo sapiens OX=9606 GN=CD82 PE=1 SV=1	16	1	1	0	57	6.5	6.76
High	Q13508	Ecto-ADP-ribosyltransferase 3 OS=Homo sapiens OX=9606 GN=ART3 PE=1 SV=2	3	1	1	0	389	43.9	6.06
High	P30050	60S ribosomal protein L12 OS=Homo sapiens OX=9606 GN=RPL12 PE=1 SV=1	11	2	2	0	165	17.8	9.42
High	P18206	Vinculin OS=Homo sapiens OX=9606 GN=VCL PE=1 SV=4	2	2	2	0	1134	123.7	5.66
High	Q14507	Epididymal secretory protein E3-alpha OS=Homo sapiens OX=9606 GN=EDDM3A PE=1 SV=2	11	2	2	0	147	17.6	8.35
High	Q4R9M9	Kinesin family member 1Bbeta isoform II OS=Homo sapiens OX=9606 GN=KIF1B PE=1 SV=1	1	1	1	0	1809	203.5	5.55
High	K7ERA3	Endoplasmic reticulum protein SC65 (Fragment) OS=Homo sapiens OX=9606 GN=P3H4 PE=1 SV=1	5	1	1	0	221	26	5.48
High	H3BUB6	Secretory carrier-associated membrane protein (Fragment) OS=Homo sapiens OX=9606 GN=SCAMP2 PE=1 SV=1	5	1	1	0	254	28.6	6.55
High	P14735	Insulin-degrading enzyme OS=Homo sapiens OX=9606 GN=IDE PE=1 SV=4	2	2	2	0	1019	117.9	6.61
High	Q9Y570	Protein phosphatase methylesterase 1 OS=Homo sapiens OX=9606 GN=PPME1 PE=1 SV=3	3	1	1	0	386	42.3	5.97
High	Q96BJ3	Axin interactor, dorsalization-associated protein OS=Homo sapiens OX=9606 GN=AIDA PE=1 SV=1	4	1	1	0	306	35	6.55
High	P09972	Fructose-bisphosphate aldolase C OS=Homo sapiens OX=9606 GN=ALDOC PE=1 SV=2	8	2	1	0	364	39.4	6.87
High	Q9NUQ8	ATP-binding cassette sub-family F member 3 OS=Homo sapiens OX=9606 GN=ABCF3 PE=1 SV=2	1	1	1	0	709	79.7	6.34
High	E9PEX6	Dihydrolipoyl dehydrogenase OS=Homo sapiens OX=9606 GN=DLD PE=1 SV=1	4	2	2	0	486	51.8	7.96
High	F8WEW9	Unconventional myosin-Ig OS=Homo sapiens OX=9606 GN=MYO1G PE=1 SV=1	9	1	1	0	210	23.6	6.8
High	P20073	Annexin A7 OS=Homo sapiens OX=9606 GN=ANXA7 PE=1 SV=3	2	1	1	0	488	52.7	5.68
High	Q9BZV1	UBX domain-containing protein 6 OS=Homo sapiens OX=9606 GN=UBXN6 PE=1 SV=1	5	2	2	0	441	49.7	6.89
High	K7EJV9	60S ribosomal protein L23a (Fragment) OS=Homo sapiens OX=9606 GN=RPL23A PE=1 SV=1	5	1	1	0	170	19.4	10.48
High	A0A0G2JRG0	Glutathione S-transferase theta-1 OS=Homo sapiens OX=9606 GN=GSTT1 PE=1 SV=1	35	2	2	0	46	5.1	6.4
High	H0Y938	Coatomer subunit beta' OS=Homo sapiens OX=9606 GN=COPB2 PE=1 SV=2	1	1	1	0	898	101.5	5.82
High	J3KMZ9	Low-density lipoprotein receptor (Fragment) OS=Homo sapiens OX=9606 GN=LDLR PE=1 SV=1	1	1	1	0	945	104.6	5.27
High	A0A087WZF1	Lipoma-preferred partner OS=Homo sapiens OX=9606 GN=LPP PE=1 SV=1	2	1	1	0	590	63.3	7.23
High	H0Y579	UV excision repair protein RAD23 homolog B (Fragment) OS=Homo sapiens OX=9606 GN=RAD23B PE=1 SV=1	14	2	2	0	114	12.2	5.52
High	P55010	Eukaryotic translation initiation factor 5 OS=Homo sapiens OX=9606 GN=EIF5 PE=1 SV=2	2	1	1	0	431	49.2	5.58
High	O14967	Calmegin OS=Homo sapiens OX=9606 GN=CLGN PE=1 SV=1	3	2	2	0	610	70	4.69
High	B0QY90	Eukaryotic translation initiation factor 3 subunit L OS=Homo sapiens OX=9606 GN=EIF3L PE=1 SV=1	2	1	1	0	466	55.1	8.95
High	Q53EL6	Programmed cell death protein 4 OS=Homo sapiens OX=9606 GN=PDCD4 PE=1 SV=2	4	1	1	0	469	51.7	5.21
High	Q9UBN7	Histone deacetylase 6 OS=Homo sapiens OX=9606 GN=HDAC6 PE=1 SV=2	1	1	1	0	1215	131.3	5.3

High	P40879	Chloride anion exchanger OS=Homo sapiens OX=9606 GN=SLC26A3 PE=1 SV=1	1	1	1	0	764	84.5	8.69
High	Q9BTE6	Alanyl-tRNA editing protein Aarsd1 OS=Homo sapiens OX=9606 GN=AARSD1 PE=1 SV=2	4	2	2	0	412	45.5	6.42
High	A0A087X142	Septin OS=Homo sapiens OX=9606 GN=SEPTIN8 PE=1 SV=1	2	1	1	0	426	49.3	6.2
High	Q13451	Peptidyl-prolyl cis-trans isomerase FKBP5 OS=Homo sapiens OX=9606 GN=FKBP5 PE=1 SV=2	4	2	2	0	457	51.2	5.9
High	Q15181	Inorganic pyrophosphatase OS=Homo sapiens OX=9606 GN=PPA1 PE=1 SV=2	3	1	1	0	289	32.6	5.86
High	Q5T3I3	NAD(P)H-hydrate epimerase OS=Homo sapiens OX=9606 GN=NAXE PE=1 SV=1	5	1	1	0	260	28.3	8.19
High	H3BQZ9	Adenine phosphoribosyltransferase OS=Homo sapiens OX=9606 GN=APRT PE=1 SV=1	14	2	2	0	153	16.6	5.59
High	O76013	Keratin, type I cuticular Ha6 OS=Homo sapiens OX=9606 GN=KRT36 PE=1 SV=1	5	2	1	0	467	52.2	4.94
High	P04049	RAF proto-oncogene serine/threonine-protein kinase OS=Homo sapiens OX=9606 GN=RAF1 PE=1 SV=1	2	1	1	0	648	73	9.2
High	O00429	Dynamin-1-like protein OS=Homo sapiens OX=9606 GN=DNM1L PE=1 SV=2	1	1	1	0	736	81.8	6.81
High	A0A087WVN4	Farnesyl pyrophosphate synthase (Fragment) OS=Homo sapiens OX=9606 GN=FDPS PE=1 SV=1	5	1	1	0	274	31.8	7.28
High	A8MTF8	Protein FAM3B OS=Homo sapiens OX=9606 GN=FAM3B PE=1 SV=2	4	1	1	0	258	28.3	8.73
High	Q5VW32	BRO1 domain-containing protein BROX OS=Homo sapiens OX=9606 GN=BROX PE=1 SV=1	3	1	1	0	411	46.4	7.65
High	P35442	Thrombospondin-2 OS=Homo sapiens OX=9606 GN=THBS2 PE=1 SV=2	1	1	1	0	1172	129.9	4.83
High	O15484	Calpain-5 OS=Homo sapiens OX=9606 GN=CAPN5 PE=1 SV=2	3	1	1	0	640	73.1	7.64
High	O00505	Importin subunit alpha-4 OS=Homo sapiens OX=9606 GN=KPNA3 PE=1 SV=2	2	1	1	0	521	57.8	4.94
High	Q9NTJ4	Alpha-mannosidase 2C1 OS=Homo sapiens OX=9606 GN=MAN2C1 PE=1 SV=1	2	2	2	0	1040	115.8	6.57
High	P62070	Ras-related protein R-Ras2 OS=Homo sapiens OX=9606 GN=RRAS2 PE=1 SV=1	5	1	1	0	204	23.4	6.01
High	O14494	Phospholipid phosphatase 1 OS=Homo sapiens OX=9606 GN=PLPP1 PE=1 SV=1	3	1	1	0	284	32.1	7.97
High	Q5T5C7	Seryl-tRNA synthetase OS=Homo sapiens OX=9606 GN=SARS1 PE=1 SV=1	2	1	1	0	536	61.3	7.06
High	J3KR23	IST1 homolog (Fragment) OS=Homo sapiens OX=9606 GN=IST1 PE=1 SV=1	3	1	1	0	289	31.2	4.75
High	O75886	Signal transducing adapter molecule 2 OS=Homo sapiens OX=9606 GN=STAM2 PE=1 SV=1	2	1	1	0	525	58.1	5.07
High	P20160	Azurocidin OS=Homo sapiens OX=9606 GN=AZU1 PE=1 SV=3	7	2	2	0	251	26.9	9.5
High	F8VZ29	Ubiquitin-conjugating enzyme E2 N OS=Homo sapiens OX=9606 GN=UBE2N PE=1 SV=1	11	1	1	0	87	9.7	7.88
High	Q14990	Outer dense fiber protein 1 OS=Homo sapiens OX=9606 GN=ODF1 PE=1 SV=2	9	2	2	0	250	28.3	8.03
High	Q86VQ3	Thioredoxin domain-containing protein 2 OS=Homo sapiens OX=9606 GN=TXNDC2 PE=1 SV=4	3	1	1	0	553	60.4	4.87
High	C9JEY1	Actin-related protein 2/3 complex subunit 1B (Fragment) OS=Homo sapiens OX=9606 GN=ARPC1B PE=1 SV=2	5	1	1	0	334	36.9	8.22
High	A0A0C4DGV8	Semaphorin-3B OS=Homo sapiens OX=9606 GN=SEMA3B PE=1 SV=1	2	1	1	0	754	83.6	8.91
High	E9PKZ0	60S ribosomal protein L8 (Fragment) OS=Homo sapiens OX=9606 GN=RPL8 PE=1 SV=1	5	1	1	0	205	22.4	10.76
High	P52888	Thimet oligopeptidase OS=Homo sapiens OX=9606 GN=THOP1 PE=1 SV=2	1	1	1	0	689	78.8	6.05
High	P22307	Sterol carrier protein 2 OS=Homo sapiens OX=9606 GN=SCP2 PE=1 SV=2	3	2	2	0	547	59	6.89
High	Q9NQX4	Unconventional myosin-Vc OS=Homo sapiens OX=9606 GN=MYO5C PE=1 SV=2	0	1	1	0	1742	202.7	7.71
High	Q687X5	Metalloreductase STEAP4 OS=Homo sapiens OX=9606 GN=STEAP4 PE=1 SV=1	2	1	1	0	459	51.9	9.29
High	D3YTI2	Acid phosphatase OS=Homo sapiens OX=9606 GN=ACP1 PE=1 SV=1	11	1	1	0	85	9.4	8.35
High	P10515	Dihydrolipoyllysine-residue acetyltransferase component of pyruvate dehydrogenase complex, mitochondrial OS=Homo sapiens OX=9606 GN=DLAT PE=1 SV=3	2	2	2	0	647	69	7.84
High	P78417	Glutathione S-transferase omega-1 OS=Homo sapiens OX=9606 GN=GSTO1 PE=1 SV=2	4	1	1	0	241	27.5	6.6
High	Q8TDZ2	[F-actin]-monooxygenase MICAL1 OS=Homo sapiens OX=9606 GN=MICAL1 PE=1 SV=2	1	1	1	0	1067	117.8	6.4
High	P48556	26S proteasome non-ATPase regulatory subunit 8 OS=Homo sapiens OX=9606 GN=PSMD8 PE=1 SV=2	2	1	1	0	350	39.6	9.7
High	P11215	Integrin alpha-M OS=Homo sapiens OX=9606 GN=ITGAM PE=1 SV=2	1	1	1	0	1152	127.1	7.23
High	Q96C24	Synaptotagmin-like protein 4 OS=Homo sapiens OX=9606 GN=SYTL4 PE=1 SV=2	3	1	1	0	671	76	8.98
High	Q8IUA0	WAP four-disulfide core domain protein 8 OS=Homo sapiens OX=9606 GN=WFDC8 PE=2 SV=2	6	2	2	0	241	27.8	8.02
High	P04080	Cystatin-B OS=Homo sapiens OX=9606 GN=CSTB PE=1 SV=2	12	1	1	0	98	11.1	7.56
High	P39656	Dolichyl-diphosphooligosaccharide--protein glycosyltransferase 48 kDa subunit OS=Homo sapiens OX=9606 GN=DDOST PE=1 SV=4	2	1	1	0	456	50.8	6.55
High	A0A2R8Y358	Testis-expressed protein 15 OS=Homo sapiens OX=9606 GN=TEX15 PE=1 SV=1	1	2	1	0	3172	358.6	6.39
High	Q9NPC4	Lactosylceramide 4-alpha-galactosyltransferase OS=Homo sapiens OX=9606 GN=A4GALT PE=1 SV=1	3	1	1	0	353	40.5	8.95
High	Q8IW45	ATP-dependent (S)-NAD(P)H-hydrate dehydratase OS=Homo sapiens OX=9606 GN=NAXD PE=1 SV=1	3	1	1	0	347	36.6	8.06
High	I3L2R6	Thioredoxin domain-containing protein 17 OS=Homo sapiens OX=9606 GN=TXNDC17 PE=1 SV=1	9	1	1	0	98	11.3	5.21
High	E9PLT1	Glycoprotein IIIb OS=Homo sapiens OX=9606 GN=CD36 PE=1 SV=1	2	1	1	0	396	44.7	8.05
High	Q969L2	Protein MAL2 OS=Homo sapiens OX=9606 GN=MAL2 PE=1 SV=1	6	1	1	0	176	19.1	6.24
High	J3KT09	Calcium-binding tyrosine phosphorylation-regulated protein (Fragment) OS=Homo sapiens OX=9606 GN=CABYR PE=1 SV=1	10	1	1	0	84	9.6	9.66
High	B7Z5N7	Sec1 family domain-containing protein 1 OS=Homo sapiens OX=9606 GN=SCFD1 PE=2 SV=1	4	1	1	0	457	51.5	6.96
High	P25325	3-mercaptopyruvate sulfurtransferase OS=Homo sapiens OX=9606 GN=MPST PE=1 SV=3	4	1	1	0	297	33.2	6.6
High	O60884	DnaJ homolog subfamily A member 2 OS=Homo sapiens OX=9606 GN=DNAJA2 PE=1 SV=1	2	1	1	0	412	45.7	6.48
High	Q92747	Actin-related protein 2/3 complex subunit 1A OS=Homo sapiens OX=9606 GN=ARPC1A PE=1 SV=2	3	1	1	0	370	41.5	8.18
High	P01833	Polymeric immunoglobulin receptor OS=Homo sapiens OX=9606 GN=PIGR PE=1 SV=4	1	1	1	0	764	83.2	5.74
High	P09417	Dihydropteridine reductase OS=Homo sapiens OX=9606 GN=QDPR PE=1 SV=2	9	2	2	0	244	25.8	7.37
High	Q15075	Early endosome antigen 1 OS=Homo sapiens OX=9606 GN=EEA1 PE=1 SV=2	1	1	1	0	1411	162.4	5.68
High	A0A2U3TZV8	Phosphoinositide phospholipase C OS=Homo sapiens OX=9606 GN=PLCH1 PE=1 SV=1	0	1	1	0	1685	188.6	7.84
High	Q9BUN1	Protein MENT OS=Homo sapiens OX=9606 GN=MENT PE=1 SV=1	2	1	1	0	341	36.7	8.59
High	Q86UD1	Out at first protein homolog OS=Homo sapiens OX=9606 GN=OAF PE=2 SV=1	3	1	1	0	273	30.7	6.84
High	O60547	GDP-mannose 4,6 dehydratase OS=Homo sapiens OX=9606 GN=GMDS PE=1 SV=1	2	1	1	0	372	41.9	7.31
High	Q9Y5K6	CD2-associated protein OS=Homo sapiens OX=9606 GN=CD2AP PE=1 SV=1	2	1	1	0	639	71.4	6.4
High	P62851	40S ribosomal protein S25 OS=Homo sapiens OX=9606 GN=RPS25 PE=1 SV=1	8	1	1	0	125	13.7	10.11
High	P54709	Sodium/potassium-transporting ATPase subunit beta-3 OS=Homo sapiens OX=9606 GN=ATP1B3 PE=1 SV=1	4	1	1	0	279	31.5	8.35
High	O15498	Synaptobrevin homolog YKT6 OS=Homo sapiens OX=9606 GN=YKT6 PE=1 SV=1	4	1	1	0	198	22.4	6.92
High	A0A2R8Y738	Ribosomal protein L15 (Fragment) OS=Homo sapiens OX=9606 GN=RPL15 PE=1 SV=1	7	1	1	0	138	16.3	10.87
High	F8WBH5	Proteasome activator complex subunit 4 OS=Homo sapiens OX=9606 GN=PSME4 PE=1 SV=1	3	1	1	0	571	65.6	7.91
High	F5H283	Testican-2 OS=Homo sapiens OX=9606 GN=SPOCK2 PE=1 SV=2	3	1	1	0	423	46.6	4.91
High	Q8TC07	TBC1 domain family member 15 OS=Homo sapiens OX=9606 GN=TBC1D15 PE=1 SV=2	1	1	1	0	691	79.4	5.67
High	Q99436	Proteasome subunit beta type-7 OS=Homo sapiens OX=9606 GN=PSMB7 PE=1 SV=1	3	1	1	0	277	29.9	7.68
High	P40429	60S ribosomal protein L13a OS=Homo sapiens OX=9606 GN=RPL13A PE=1 SV=2	7	2	2	0	203	23.6	10.93
High	F8VR77	Proliferation-associated protein 2G4 OS=Homo sapiens OX=9606 GN=PA2G4 PE=1 SV=1	3	1	1	0	284	31.4	6.8
High	P61353	60S ribosomal protein L27 OS=Homo sapiens OX=9606 GN=RPL27 PE=1 SV=2	6	1	1	0	136	15.8	10.56
High	Q9BQA1	Methylosome protein 50 OS=Homo sapiens OX=9606 GN=WDR77 PE=1 SV=1	2	1	1	0	342	36.7	5.17

High	P04217	Alpha-1B-glycoprotein OS=Homo sapiens OX=9606 GN=A1BG PE=1 SV=4	2	1	1	0	495	54.2	5.86
High	F6U784	Ras-related protein Rap-2a OS=Homo sapiens OX=9606 GN=RAP2A PE=4 SV=1	10	1	1	0	108	12.3	5.52
High	P28066	Proteasome subunit alpha type-5 OS=Homo sapiens OX=9606 GN=PSMA5 PE=1 SV=3	4	1	1	0	241	26.4	4.79
High	H3BNT7	26S proteasome non-ATPase regulatory subunit 7 OS=Homo sapiens OX=9606 GN=PSMD7 PE=1 SV=1	5	1	1	0	177	20.1	5.57
High	E7ET01	Cytoplasmic dynein 1 intermediate chain 2 (Fragment) OS=Homo sapiens OX=9606 GN=DYNC1I2 PE=1 SV=1	8	1	1	0	173	19.3	5.29
High	H3BS53	Disintegrin and metalloproteinase domain-containing protein 10 (Fragment) OS=Homo sapiens OX=9606 GN=ADAM10 PE=1 SV=2	19	1	1	0	58	6.6	4.83
High	F5GXS0	C4a anaphylatoxin OS=Homo sapiens OX=9606 GN=C4B PE=1 SV=1	1	1	1	0	1698	187.6	7.33
High	F5GX62	Ras-related protein Rap-1b (Fragment) OS=Homo sapiens OX=9606 GN=RAP1B PE=1 SV=1	8	1	1	0	139	15.7	4.74
High	Q15274	Nicotinate-nucleotide pyrophosphorylase [carboxylating] OS=Homo sapiens OX=9606 GN=QPRT PE=1 SV=3	3	1	1	0	297	30.8	6.21
High	Q9Y266	Nuclear migration protein nudC OS=Homo sapiens OX=9606 GN=NUDC PE=1 SV=1	3	1	1	0	331	38.2	5.38
High	Q71UM5	40S ribosomal protein S27-like OS=Homo sapiens OX=9606 GN=RPS27L PE=1 SV=3	10	1	1	0	84	9.5	9.45
High	Q9BV40	Vesicle-associated membrane protein 8 OS=Homo sapiens OX=9606 GN=VAMP8 PE=1 SV=1	14	1	1	0	100	11.4	7.34
High	O43182	Rho GTPase-activating protein 6 OS=Homo sapiens OX=9606 GN=ARHGAP6 PE=1 SV=3	1	1	1	0	974	105.9	7.36
High	P57735	Ras-related protein Rab-25 OS=Homo sapiens OX=9606 GN=RAB25 PE=1 SV=2	5	1	1	0	213	23.5	5.96
High	Q8WTS6	Histone-lysine N-methyltransferase SETD7 OS=Homo sapiens OX=9606 GN=SETD7 PE=1 SV=1	2	1	1	0	366	40.7	4.63
High	K7EKQ2	Hsp90 co-chaperone Cdc37 (Fragment) OS=Homo sapiens OX=9606 GN=CDC37 PE=1 SV=1	2	1	1	0	353	41.6	6.64
High	S4R3Q6	Vacuolar protein sorting-associated protein 26A OS=Homo sapiens OX=9606 GN=VPS26A PE=1 SV=1	5	1	1	0	226	26.2	6.24
High	A8MXL6	GATOR complex protein SEC13 OS=Homo sapiens OX=9606 GN=SEC13 PE=1 SV=1	4	1	1	0	284	31.6	5.82
High	P15291	Beta-1,4-galactosyltransferase 1 OS=Homo sapiens OX=9606 GN=B4GALT1 PE=1 SV=5	2	1	1	0	398	43.9	8.65
High	A6NFX8	ADP-sugar pyrophosphatase OS=Homo sapiens OX=9606 GN=NUDT5 PE=1 SV=1	4	1	1	0	232	25.9	5.19
High	H3BQ15	Uncharacterized protein (Fragment) OS=Homo sapiens OX=9606 PE=4 SV=1	7	1	1	0	152	16	6.64
High	O43633	Charged multivesicular body protein 2a OS=Homo sapiens OX=9606 GN=CHMP2A PE=1 SV=1	4	1	1	0	222	25.1	5.97
High	M0R3D6	60S ribosomal protein L18a (Fragment) OS=Homo sapiens OX=9606 GN=RPL18A PE=1 SV=1	6	1	1	0	141	16.7	10.77
High	Q9UHL4	Dipeptidyl peptidase 2 OS=Homo sapiens OX=9606 GN=DPP7 PE=1 SV=3	2	1	1	0	492	54.3	6.32
High	Q14749	Glycine N-methyltransferase OS=Homo sapiens OX=9606 GN=GNMT PE=1 SV=3	3	1	1	0	295	32.7	7.02
High	P22061	Protein-L-isoaspartate(D-aspartate) O-methyltransferase OS=Homo sapiens OX=9606 GN=PCMT1 PE=1 SV=4	4	1	1	0	227	24.6	7.21
High	P19075	Tetraspanin-8 OS=Homo sapiens OX=9606 GN=TSPAN8 PE=1 SV=1	4	1	1	0	237	26	5.6
High	H0YC99	Latent-transforming growth factor beta-binding protein 3 (Fragment) OS=Homo sapiens OX=9606 GN=LTBP3 PE=1 SV=1	1	1	1	0	907	98.8	5.4
High	Q5QPQ0	Acyl-protein thioesterase 2 OS=Homo sapiens OX=9606 GN=LYPLA2 PE=1 SV=1	5	1	1	0	164	17.6	7.42
High	P62269	40S ribosomal protein S18 OS=Homo sapiens OX=9606 GN=RPS18 PE=1 SV=3	7	1	1	0	152	17.7	10.99
High	K7ER96	Thioredoxin-like protein 1 (Fragment) OS=Homo sapiens OX=9606 GN=TXNL1 PE=1 SV=1	2	1	1	0	281	31.4	4.83
High	P53004	Biliverdin reductase A OS=Homo sapiens OX=9606 GN=BLVRA PE=1 SV=2	3	1	1	0	296	33.4	6.44
High	D6RE99	Histidine triad nucleotide-binding protein 1 OS=Homo sapiens OX=9606 GN=HINT1 PE=1 SV=1	9	1	1	0	78	8.6	5.39
High	A0A2R8YDP2	Casein kinase II subunit alpha OS=Homo sapiens OX=9606 GN=CSNK2A1 PE=1 SV=1	2	1	1	0	354	40.6	8.03
High	P28331	NADH-ubiquinone oxidoreductase 75 kDa subunit, mitochondrial OS=Homo sapiens OX=9606 GN=NDUFS1 PE=1 SV=3	1	1	1	0	727	79.4	6.23
High	A0A590UK80	Uncharacterized protein (Fragment) OS=Homo sapiens OX=9606 PE=3 SV=1	1	1	1	0	1090	121	6.84
High	Q5VVC8	60S ribosomal protein L11 OS=Homo sapiens OX=9606 GN=RPL11 PE=1 SV=2	4	1	1	0	167	19	9.8
High	P17655	Calpain-2 catalytic subunit OS=Homo sapiens OX=9606 GN=CAPN2 PE=1 SV=6	1	1	1	0	700	79.9	4.98
High	P56851	Epididymal secretory protein E3-beta OS=Homo sapiens OX=9606 GN=EDDM3B PE=1 SV=2	5	1	1	0	147	17.6	6.99
High	A0A075B6R0	T cell receptor gamma variable 2 OS=Homo sapiens OX=9606 GN=TRGV2 PE=1 SV=1	9	1	1	0	118	13.3	7.97
High	Q6X4U4	Sclerostin domain-containing protein 1 OS=Homo sapiens OX=9606 GN=SOSTDC1 PE=1 SV=2	4	1	1	0	206	23.3	9.74
High	P46783	40S ribosomal protein S10 OS=Homo sapiens OX=9606 GN=RPS10 PE=1 SV=1	6	1	1	0	165	18.9	10.15
High	G3V582	Molybdopterin molybdenumtransferase OS=Homo sapiens OX=9606 GN=GPHN PE=1 SV=1	3	1	1	0	380	41.3	6.71
High	E9PQM1	Cathepsin B OS=Homo sapiens OX=9606 GN=CTSB PE=1 SV=2	4	1	1	0	283	31.3	6.76
High	O75683	Surfeit locus protein 6 OS=Homo sapiens OX=9606 GN=SURF6 PE=1 SV=3	2	1	1	0	361	41.4	10.64
High	Q5VWZ2	Lysophospholipase-like protein 1 OS=Homo sapiens OX=9606 GN=LYPLAL1 PE=1 SV=3	4	1	1	0	237	26.3	7.84
High	B4DKY1	Cysteinyl-tRNA synthetase OS=Homo sapiens OX=9606 GN=CARS1 PE=1 SV=1	1	1	1	0	739	84.2	7.05
High	P10599	Thioredoxin OS=Homo sapiens OX=9606 GN=TXN PE=1 SV=3	10	1	1	0	105	11.7	4.92
High	O75610	Left-right determination factor 1 OS=Homo sapiens OX=9606 GN=LEFTY1 PE=2 SV=1	2	1	1	0	366	40.9	8.27
High	A0A286YF97	Mitogen-activated protein kinase OS=Homo sapiens OX=9606 GN=MAPK10 PE=1 SV=1	2	1	1	0	464	52.3	6.54
High	D6RFN0	COP9 signalosome complex subunit 4 OS=Homo sapiens OX=9606 GN=COPS4 PE=1 SV=1	3	1	1	0	438	49.7	5.81
High	A0A7I2V562	Cytospin-B OS=Homo sapiens OX=9606 GN=SPECC1 PE=4 SV=1	2	1	1	0	834	92.5	5.83
High	Q14195	Dihydropyrimidinase-related protein 3 OS=Homo sapiens OX=9606 GN=DPYSL3 PE=1 SV=1	2	1	1	0	570	61.9	6.49
High	P48147	Prolyl endopeptidase OS=Homo sapiens OX=9606 GN=PREP PE=1 SV=2	1	1	1	0	710	80.6	5.86
High	Q13131	5'-AMP-activated protein kinase catalytic subunit alpha-1 OS=Homo sapiens OX=9606 GN=PRKAA1 PE=1 SV=4	1	1	1	0	559	64	8.12
High	Q5T371	Arrestin domain-containing protein 1 (Fragment) OS=Homo sapiens OX=9606 GN=ARRDC1 PE=1 SV=1	8	1	1	0	177	19.1	9.63
High	Q9BSH5	Haloacid dehalogenase-like hydrolase domain-containing protein 3 OS=Homo sapiens OX=9606 GN=HDHD3 PE=1 SV=1	4	1	1	0	251	28	6.71
High	P62277	40S ribosomal protein S13 OS=Homo sapiens OX=9606 GN=RPS13 PE=1 SV=2	5	1	1	0	151	17.2	10.54
High	A6NDG6	Glycerol-3-phosphate phosphatase OS=Homo sapiens OX=9606 GN=PGP PE=1 SV=1	3	1	1	0	321	34	6.14
High	Q86X76	Deaminated glutathione amidase OS=Homo sapiens OX=9606 GN=NIT1 PE=1 SV=2	2	1	1	0	327	35.9	7.74
High	E9PIB2	Gasdermin-D (Fragment) OS=Homo sapiens OX=9606 GN=GSDMD PE=1 SV=1	4	1	1	0	277	30.8	9.04
High	A8MQB8	Synaptic functional regulator FMR1 OS=Homo sapiens OX=9606 GN=FMR1 PE=1 SV=1	1	1	1	0	582	65.8	7.62
High	H0Y380	Protein-tyrosine-phosphatase (Fragment) OS=Homo sapiens OX=9606 GN=PTPRF PE=1 SV=1	1	1	1	0	979	111.1	6.61
High	F8VQQ3	MYG1 exonuclease OS=Homo sapiens OX=9606 GN=MYG1 PE=1 SV=1	3	1	1	0	301	33.8	7.02
Medium	D3YTI8	60S ribosomal protein L32 (Fragment) OS=Homo sapiens OX=9606 GN=RPL32 PE=1 SV=1	18	1	1	0	62	7.5	12.09
Medium	E9PNF5	Glutamine-dependent NAD(+) synthetase (Fragment) OS=Homo sapiens OX=9606 GN=NADSYN1 PE=1 SV=1	7	1	1	0	149	16.1	7.72
Medium	F8WF69	Clathrin light chain OS=Homo sapiens OX=9606 GN=CLTA PE=1 SV=1	3	1	1	0	260	27.8	4.91
Medium	Q9BXJ1	Complement C1q tumor necrosis factor-related protein 1 OS=Homo sapiens OX=9606 GN=C1QTNF1 PE=1 SV=1	4	1	1	0	281	31.7	6.9
Medium	Q13206	Probable ATP-dependent RNA helicase DDX10 OS=Homo sapiens OX=9606 GN=DDX10 PE=1 SV=2	1	1	1	0	875	100.8	8.63
Medium	P49207	60S ribosomal protein L34 OS=Homo sapiens OX=9606 GN=RPL34 PE=1 SV=3	6	1	1	0	117	13.3	11.47

Medium	B7Z2U2	TOM1-like protein 2 OS=Homo sapiens OX=9606 GN=TOM1L2 PE=1 SV=1	2	1	1	0	389	42.9	4.79
Medium	P00966	Argininosuccinate synthase OS=Homo sapiens OX=9606 GN=ASS1 PE=1 SV=2	2	1	1	0	412	46.5	8.02
Medium	Q9P225	Dynein heavy chain 2, axonemal OS=Homo sapiens OX=9606 GN=DNAH2 PE=1 SV=3	0	1	1	0	4427	507.4	6.37
Medium	P41220	Regulator of G-protein signaling 2 OS=Homo sapiens OX=9606 GN=RGS2 PE=1 SV=1	4	1	1	0	211	24.4	8.91
Medium	E7ESK6	Syndecan OS=Homo sapiens OX=9606 GN=SDC2 PE=1 SV=1	5	1	1	0	165	18.4	6.05
Medium	Q00169	Phosphatidylinositol transfer protein alpha isoform OS=Homo sapiens OX=9606 GN=PITPNA PE=1 SV=2	5	1	1	0	270	31.8	6.55
Medium	H0Y542	Breast carcinoma-amplified sequence 1 (Fragment) OS=Homo sapiens OX=9606 GN=BCAS1 PE=1 SV=1	2	1	1	0	476	51.1	7.93
Medium	P09936	Ubiquitin carboxyl-terminal hydrolase isozyme L1 OS=Homo sapiens OX=9606 GN=UCHL1 PE=1 SV=2	4	1	1	0	223	24.8	5.48
Medium	Q9Y6Q5	AP-1 complex subunit mu-2 OS=Homo sapiens OX=9606 GN=AP1M2 PE=1 SV=4	2	1	1	0	423	48.1	8.22
Medium	C9J1W3	AT-rich interactive domain-containing protein 4A (Fragment) OS=Homo sapiens OX=9606 GN=ARID4A PE=1 SV=1	3	1	1	0	265	30	4.73
Medium	Q99732	Lipopolysaccharide-induced tumor necrosis factor-alpha factor OS=Homo sapiens OX=9606 GN=LITAF PE=1 SV=2	4	1	1	0	161	17.1	6.44
Medium	Q13126	S-methyl-5'-thioadenosine phosphorylase OS=Homo sapiens OX=9606 GN=MTAP PE=1 SV=2	6	1	1	0	283	31.2	7.18
Medium	P00441	Superoxide dismutase [Cu-Zn] OS=Homo sapiens OX=9606 GN=SOD1 PE=1 SV=2	5	1	1	0	154	15.9	6.13
Medium	Q9H0R4	Haloacid dehalogenase-like hydrolase domain-containing protein 2 OS=Homo sapiens OX=9606 GN=HDHD2 PE=1 SV=1	3	1	1	0	259	28.5	6.24
Medium	Q9H993	Damage-control phosphatase ARMT1 OS=Homo sapiens OX=9606 GN=ARMT1 PE=1 SV=1	2	1	1	0	441	51.1	5.76
Medium	P08236	Beta-glucuronidase OS=Homo sapiens OX=9606 GN=GUSB PE=1 SV=2	1	1	1	0	651	74.7	7.02
Medium	G3V3R6	Galectin OS=Homo sapiens OX=9606 GN=LGALS3 PE=1 SV=1	3	1	1	0	233	24.1	9.39
Medium	Q5TFJ7	Importin subunit alpha-7 (Fragment) OS=Homo sapiens OX=9606 GN=KPNA6 PE=1 SV=1	3	1	1	0	321	35.4	5.43
Medium	H0Y512	Adipocyte plasma membrane-associated protein (Fragment) OS=Homo sapiens OX=9606 GN=APMAP PE=1 SV=1	2	1	1	0	409	45.4	5.66
Medium	P05060	Secretogranin-1 OS=Homo sapiens OX=9606 GN=CHGB PE=1 SV=2	2	1	1	0	677	78.2	5.07
Medium	A0A6Q8PFF8	Charged multivesicular body protein 1a OS=Homo sapiens OX=9606 GN=CHMP1A PE=1 SV=1	3	1	1	0	265	27.9	7.59
Medium	J3KRJ4	COP9 signalosome complex subunit 1 (Fragment) OS=Homo sapiens OX=9606 GN=GPS1 PE=1 SV=1	3	1	1	0	253	27.8	4.91
Medium	P23763	Vesicle-associated membrane protein 1 OS=Homo sapiens OX=9606 GN=VAMP1 PE=1 SV=1	6	1	1	0	118	12.9	6.65
Medium	Q15750	TGF-beta-activated kinase 1 and MAP3K7-binding protein 1 OS=Homo sapiens OX=9606 GN=TAB1 PE=1 SV=1	2	1	1	0	504	54.6	5.52
Medium	D6RD47	40S ribosomal protein S23 OS=Homo sapiens OX=9606 GN=RPS23 PE=1 SV=1	8	1	1	0	134	14.8	10.23
Medium	Q96JB2	Conserved oligomeric Golgi complex subunit 3 OS=Homo sapiens OX=9606 GN=COG3 PE=1 SV=3	2	1	1	0	828	94	5.57
Medium	Q15785	Mitochondrial import receptor subunit TOM34 OS=Homo sapiens OX=9606 GN=TOMM34 PE=1 SV=2	4	1	1	0	309	34.5	8.98
Medium	P10827	Thyroid hormone receptor alpha OS=Homo sapiens OX=9606 GN=THRA PE=1 SV=1	2	1	1	0	490	54.8	6.86
Medium	A0A7I2V4U6	60S ribosomal protein L19 OS=Homo sapiens OX=9606 GN=RPL19 PE=4 SV=1	7	1	1	0	95	10.9	11.84
Medium	E7EUI6	Fibronectin receptor subunit beta OS=Homo sapiens OX=9606 GN=ITGB1 PE=1 SV=2	1	1	1	0	801	88.7	5.44
Medium	A0A0A0MSB8	Exocyst complex component 7 OS=Homo sapiens OX=9606 GN=EXOC7 PE=1 SV=1	1	1	1	0	693	78.8	6.55
Medium	E7ESC6	Exportin-7 OS=Homo sapiens OX=9606 GN=XPO7 PE=1 SV=1	1	1	1	0	1088	124	6.48
Medium	H3BP71	E3 ubiquitin protein ligase OS=Homo sapiens OX=9606 GN=RNF40 PE=1 SV=2	1	1	1	0	961	109	6.55
Medium	P48449	Lanosterol synthase OS=Homo sapiens OX=9606 GN=LSS PE=1 SV=1	2	1	1	0	732	83.3	6.61
Medium	A0A1B0GV03	Golgin subfamily A member 6-like protein 7 OS=Homo sapiens OX=9606 GN=GOLGA6L7 PE=3 SV=1	1	1	1	0	622	75.5	5.34
Medium	P03973	Antileukoproteinase OS=Homo sapiens OX=9606 GN=SLPI PE=1 SV=2	6	1	1	0	132	14.3	8.75
Medium	E9PLX4	Granzyme B OS=Homo sapiens OX=9606 GN=GZMB PE=4 SV=1	30	1	1	0	44	4.9	5.1
Medium	Q96HU1	Small G protein signaling modulator 3 OS=Homo sapiens OX=9606 GN=SGSM3 PE=1 SV=1	1	1	1	0	749	85.3	6
Medium	E7EX73	Eukaryotic translation initiation factor 4 gamma 1 OS=Homo sapiens OX=9606 GN=EIF4G1 PE=1 SV=1	1	1	1	0	1436	158.5	5.21
Medium	Q96LB3	Intraflagellar transport protein 74 homolog OS=Homo sapiens OX=9606 GN=IFT74 PE=1 SV=1	2	1	1	0	600	69.2	6
Medium	A8MUM1	EARP and GARP complex-interacting protein 1 OS=Homo sapiens OX=9606 GN=EIPR1 PE=1 SV=2	2	1	1	0	414	46.3	5.07
Medium	Q5EBL4	RILP-like protein 1 OS=Homo sapiens OX=9606 GN=RILPL1 PE=1 SV=1	2	1	1	0	403	47.1	5.21
Medium	H7BZF5	Uncharacterized protein C2orf80 (Fragment) OS=Homo sapiens OX=9606 GN=C2orf80 PE=4 SV=1	6	1	1	0	139	15.7	9.83
Medium	Q5XKR4	Homeobox protein orthopedia OS=Homo sapiens OX=9606 GN=OTP PE=1 SV=1	5	1	1	0	325	34.1	9.47
Medium	A0A7N4I390	NTPase KAP family P-loop domain-containing protein 1 OS=Homo sapiens OX=9606 GN=NKPD1 PE=4 SV=1	1	1	1	0	832	91.8	8.9
Medium	Q12929	Epidermal growth factor receptor kinase substrate 8 OS=Homo sapiens OX=9606 GN=EPS8 PE=1 SV=1	1	1	1	0	822	91.8	7.5
Medium	O75477	Erlin-1 OS=Homo sapiens OX=9606 GN=ERLIN1 PE=1 SV=2	2	1	1	0	348	39.1	7.87
Medium	F5H815	Importin-8 OS=Homo sapiens OX=9606 GN=IPO8 PE=1 SV=1	34	1	1	0	35	4	5.02
Medium	A0A2R8Y688	Trifunctional enzyme subunit alpha, mitochondrial OS=Homo sapiens OX=9606 GN=HADHA PE=1 SV=1	5	1	1	0	357	39.2	9.22
Medium	H7C2Y5	DnaJ_C domain-containing protein (Fragment) OS=Homo sapiens OX=9606 PE=1 SV=8	5	1	1	0	167	19.2	5.24
Medium	Q6UX06	Olfactomedin-4 OS=Homo sapiens OX=9606 GN=OLFM4 PE=1 SV=1	1	1	1	0	510	57.2	5.69
Medium	Q92954	Proteoglycan 4 OS=Homo sapiens OX=9606 GN=PRG4 PE=1 SV=3	0	1	1	0	1404	151	9.51
Medium	Q5JSZ5	Protein PRRC2B OS=Homo sapiens OX=9606 GN=PRRC2B PE=1 SV=2	0	1	1	0	2229	242.8	8.34
Medium	C9J590	Replication initiator 1 OS=Homo sapiens OX=9606 GN=REPIN1 PE=4 SV=2	10	1	1	0	136	15.3	11.55
Medium	Q13155	Aminoacyl tRNA synthase complex-interacting multifunctional protein 2 OS=Homo sapiens OX=9606 GN=AIMP2 PE=1 SV=2	4	1	1	0	320	35.3	8.22
Medium	E7ENN3	Nesprin-1 OS=Homo sapiens OX=9606 GN=SYNE1 PE=1 SV=2	0	1	1	0	8392	964.2	5.54
Medium	A0A2R8Y7Q1	Nucleosome-remodeling factor subunit BPTF OS=Homo sapiens OX=9606 GN=BPTF PE=1 SV=1	0	1	1	0	2965	330.6	6.1
Medium	A0A087WZI5	Isoaspartyl peptidase/L-asparaginase (Fragment) OS=Homo sapiens OX=9606 GN=ASRGL1 PE=1 SV=6	8	1	1	0	133	13.9	5.15
Medium	Q8N392	Rho GTPase-activating protein 18 OS=Homo sapiens OX=9606 GN=ARHGAP18 PE=1 SV=3	1	1	1	0	663	74.9	6.44
Medium	A0A024R0K5	Carcinoembryonic antigen-related cell adhesion molecule 5 OS=Homo sapiens OX=9606 GN=CEACAM5 PE=1 SV=1	1	1	1	0	702	76.7	5.69
Medium	H0YHF8	5'-AMP-activated protein kinase subunit gamma-1 (Fragment) OS=Homo sapiens OX=9606 GN=PRKAG1 PE=1 SV=1	12	1	1	0	93	10.7	5.2
Medium	F8W0I7	Netrin-4 (Fragment) OS=Homo sapiens OX=9606 GN=NTN4 PE=1 SV=1	9	1	1	0	176	19.9	6.87
Medium	A0A0A0MRF6	A-kinase anchor protein 9 OS=Homo sapiens OX=9606 GN=AKAP9 PE=1 SV=2	0	1	1	0	3931	455.6	5
Medium	M0R2L2	TBC1 domain family member 17 (Fragment) OS=Homo sapiens OX=9606 GN=TBC1D17 PE=1 SV=2	2	1	1	0	480	53.9	5.72
Medium	Q16222	UDP-N-acetylhexosamine pyrophosphorylase OS=Homo sapiens OX=9606 GN=UAP1 PE=1 SV=3	1	1	1	0	522	58.7	6.33
Medium	P50747	Biotin--protein ligase OS=Homo sapiens OX=9606 GN=HLCS PE=1 SV=1	1	1	1	0	726	80.7	5.62
Medium	Q9NR48	Histone-lysine N-methyltransferase ASH1L OS=Homo sapiens OX=9606 GN=ASH1L PE=1 SV=2	0	1	1	0	2969	332.6	9.39
Medium	E9PRZ4	Membrane-anchored junction protein OS=Homo sapiens OX=9606 GN=MAJIN PE=1 SV=1	10	1	1	0	114	13.5	7.37
Medium	Q9H7F0	Probable cation-transporting ATPase 13A3 OS=Homo sapiens OX=9606 GN=ATP13A3 PE=1 SV=4	1	1	1	0	1226	138	6.64
Medium	Q8WW22	DnaJ homolog subfamily A member 4 OS=Homo sapiens OX=9606 GN=DNAJA4 PE=1 SV=1	2	1	1	0	397	44.8	7.59
Medium	Q9NXV6	CDKN2A-interacting protein OS=Homo sapiens OX=9606 GN=CDKN2AIP PE=1 SV=3	3	1	1	0	580	61.1	9.01

Medium	Q09666	Neuroblast differentiation-associated protein AHNAK OS=Homo sapiens OX=9606 GN=AHNAK PE=1 SV=2	0	1	1	0	5890	628.7	6.15
Medium	O95067	G2/mitotic-specific cyclin-B2 OS=Homo sapiens OX=9606 GN=CCNB2 PE=1 SV=1	2	1	1	0	398	45.3	8.9
Medium	K7EKW9	Flotillin (Fragment) OS=Homo sapiens OX=9606 GN=FLOT2 PE=1 SV=1	4	1	1	0	249	28	4.91
Medium	Q96L34	MAP/microtubule affinity-regulating kinase 4 OS=Homo sapiens OX=9606 GN=MARK4 PE=1 SV=1	1	1	1	0	752	82.5	9.66
Medium	E7ENQ1	Mitogen-activated protein kinase kinase kinase kinase 4 OS=Homo sapiens OX=9606 GN=MAP4K4 PE=1 SV=1	1	1	1	0	1154	132.1	7.53
Medium	P0C7P3	Protein SLFN14 OS=Homo sapiens OX=9606 GN=SLFN14 PE=1 SV=2	1	1	1	0	912	103.8	8.24
Medium	P49917	DNA ligase 4 OS=Homo sapiens OX=9606 GN=LIG4 PE=1 SV=2	2	1	1	0	911	103.9	7.96
Medium	K7EL88	Beclin-1 (Fragment) OS=Homo sapiens OX=9606 GN=BECN1 PE=1 SV=1	25	1	1	0	55	6.3	7.44
Medium	P15924	Desmoplakin OS=Homo sapiens OX=9606 GN=DSP PE=1 SV=3	0	1	1	0	2871	331.6	6.81
Medium	H3BSV3	Cadherin-16 OS=Homo sapiens OX=9606 GN=CDH16 PE=1 SV=1	4	1	1	0	248	27.3	5.31
Medium	Q5VU57	Cytosolic carboxypeptidase 6 OS=Homo sapiens OX=9606 GN=AGBL4 PE=2 SV=3	2	1	1	0	503	58.2	8.41
Medium	A0A087X2D0	Serine/arginine-rich-splicing factor 3 OS=Homo sapiens OX=9606 GN=SRSF3 PE=1 SV=1	9	1	1	0	95	10.3	5.14
Medium	A0A3B3ISX3	Translation initiation factor eIF-2B subunit epsilon OS=Homo sapiens OX=9606 GN=EIF2B5 PE=1 SV=1	1	1	1	0	530	58.3	6.61
Medium	A5YKK6	CCR4-NOT transcription complex subunit 1 OS=Homo sapiens OX=9606 GN=CNOT1 PE=1 SV=2	1	1	1	0	2376	266.8	7.11
Medium	Q9NXZ2	Probable ATP-dependent RNA helicase DDX43 OS=Homo sapiens OX=9606 GN=DDX43 PE=1 SV=2	2	1	1	0	648	72.8	8.75
Medium	Q6PCB7	Long-chain fatty acid transport protein 1 OS=Homo sapiens OX=9606 GN=SLC27A1 PE=1 SV=1	1	1	1	0	646	71.1	8.53
Medium	O75165	DnaJ homolog subfamily C member 13 OS=Homo sapiens OX=9606 GN=DNAJC13 PE=1 SV=5	0	1	1	0	2243	254.3	6.74
Medium	Q8WY64	E3 ubiquitin-protein ligase MYLIP OS=Homo sapiens OX=9606 GN=MYLIP PE=1 SV=2	3	1	1	0	445	49.9	7.24
Medium	D6RC51	Serine protease inhibitor Kazal-type 2 OS=Homo sapiens OX=9606 GN=SPINK2 PE=1 SV=1	8	1	1	0	119	12.4	8.81
Medium	Q86V21	Acetoacetyl-CoA synthetase OS=Homo sapiens OX=9606 GN=AACS PE=1 SV=1	1	1	1	0	672	75.1	6.24
Medium	F2Z2Y4	Pyridoxal kinase OS=Homo sapiens OX=9606 GN=PDXK PE=1 SV=1	4	1	1	0	272	30.6	6.65
Medium	Q9Y6W3	Calpain-7 OS=Homo sapiens OX=9606 GN=CAPN7 PE=1 SV=1	3	1	1	0	813	92.6	7.65
Medium	Q8NHQ9	ATP-dependent RNA helicase DDX55 OS=Homo sapiens OX=9606 GN=DDX55 PE=1 SV=3	1	1	1	0	600	68.5	9.25
Medium	P29966	Myristoylated alanine-rich C-kinase substrate OS=Homo sapiens OX=9606 GN=MARCKS PE=1 SV=4	2	1	1	0	332	31.5	4.45
Medium	Q9NVE7	4'-phosphopantetheine phosphatase OS=Homo sapiens OX=9606 GN=PANK4 PE=1 SV=1	1	1	1	0	773	85.9	6.28
Medium	F5H365	Protein transport protein SEC23 OS=Homo sapiens OX=9606 GN=SEC23A PE=1 SV=1	1	1	1	0	736	82.9	7.46
Medium	P19113	Histidine decarboxylase OS=Homo sapiens OX=9606 GN=HDC PE=1 SV=2	2	1	1	0	662	74.1	8
Medium	Q96JH7	Deubiquitinating protein VCPIP1 OS=Homo sapiens OX=9606 GN=VCPIP1 PE=1 SV=2	1	1	1	0	1222	134.2	7.2
Medium	Q6OI27	ALS2 C-terminal-like protein OS=Homo sapiens OX=9606 GN=ALS2CL PE=1 SV=1	1	1	1	0	953	107.7	6.15
Medium	E5RIT6	60S ribosomal protein L26-like 1 (Fragment) OS=Homo sapiens OX=9606 GN=RPL26L1 PE=1 SV=1	5	1	1	0	128	15.2	11.09
High	E7EQB2	Lactotransferrin (Fragment) OS=Homo sapiens OX=9606 GN=LTF PE=1 SV=1	33	25	25	0	696	76.6	8.02
High	P12273	Prolactin-inducible protein OS=Homo sapiens OX=9606 GN=PIP PE=1 SV=1	69	11	11	0	146	16.6	8.05
High	P63261	Actin, cytoplasmic 2 OS=Homo sapiens OX=9606 GN=ACTG1 PE=1 SV=1	49	13	13	0	375	41.8	5.48
High	P02751	Fibronectin OS=Homo sapiens OX=9606 GN=FN1 PE=1 SV=5	6	10	10	0	2477	272.2	5.5
High	P02768	Albumin OS=Homo sapiens OX=9606 GN=ALB PE=1 SV=2	21	10	10	0	609	69.3	6.28
High	P04279	Semenogelin-1 OS=Homo sapiens OX=9606 GN=SEMG1 PE=1 SV=2	16	5	3	2	462	52.1	9.29
High	P10909	Clusterin OS=Homo sapiens OX=9606 GN=CLU PE=1 SV=1	16	10	10	0	449	52.5	6.27
High	P15309	Prostatic acid phosphatase OS=Homo sapiens OX=9606 GN=ACP3 PE=1 SV=3	13	5	5	0	386	44.5	6.24
High	P12277	Creatine kinase B-type OS=Homo sapiens OX=9606 GN=CKB PE=1 SV=1	10	3	3	0	381	42.6	5.59
High	Q02383	Semenogelin-2 OS=Homo sapiens OX=9606 GN=SEMG2 PE=1 SV=1	15	7	5	2	582	65.4	9.07
High	P13796	Plastin-2 OS=Homo sapiens OX=9606 GN=LCP1 PE=1 SV=6	7	4	2	2	627	70.2	5.43
High	A0A0A0MSQ0	Plastin-3 OS=Homo sapiens OX=9606 GN=PLS3 PE=1 SV=1	6	3	1	0	617	69.3	5.94
High	P07900	Heat shock protein HSP 90-alpha OS=Homo sapiens OX=9606 GN=HSP90AA1 PE=1 SV=5	6	3	3	0	732	84.6	5.02
High	E9PKE3	Heat shock cognate 71 kDa protein OS=Homo sapiens OX=9606 GN=HSPA8 PE=1 SV=1	5	2	2	0	627	68.8	5.52
High	P15311	Ezrin OS=Homo sapiens OX=9606 GN=EZR PE=1 SV=4	4	2	2	0	586	69.4	6.27
High	P06858	Lipoprotein lipase OS=Homo sapiens OX=9606 GN=LPL PE=1 SV=1	6	2	2	0	475	53.1	8.15
High	P08670	Vimentin OS=Homo sapiens OX=9606 GN=VIM PE=1 SV=4	4	1	1	0	466	53.6	5.12
High	M0QZF9	Prostate-specific antigen (Fragment) OS=Homo sapiens OX=9606 GN=KLK3 PE=1 SV=1	16	2	2	0	172	19.1	6.98
High	P54107	Cysteine-rich secretory protein 1 OS=Homo sapiens OX=9606 GN=CRISP1 PE=1 SV=1	8	1	1	0	249	28.5	5.91
High	P78371	T-complex protein 1 subunit beta OS=Homo sapiens OX=9606 GN=CCT2 PE=1 SV=4	4	1	1	0	535	57.5	6.46
High	A0A0J9YX77	Maltase-glucoamylase, intestinal (Fragment) OS=Homo sapiens OX=9606 GN=MGAM PE=1 SV=1	1	1	1	0	1114	126.5	5.24
High	Q66K79	Carboxypeptidase Z OS=Homo sapiens OX=9606 GN=CPZ PE=1 SV=2	2	1	1	0	652	73.6	7.97
High	P80303	Nucleobindin-2 OS=Homo sapiens OX=9606 GN=NUCB2 PE=1 SV=3	2	1	1	0	420	50.2	5.12
High	P13473	Lysosome-associated membrane glycoprotein 2 OS=Homo sapiens OX=9606 GN=LAMP2 PE=1 SV=2	2	1	1	0	410	44.9	5.63
High	P62805	Histone H4 OS=Homo sapiens OX=9606 GN=H4C1 PE=1 SV=2	8	1	1	0	103	11.4	11.36
High	G5E9W0	Phospholipase A1 member A OS=Homo sapiens OX=9606 GN=PLA1A PE=1 SV=1	5	1	1	0	440	48.1	7.72
High	Q16778	Histone H2B type 2-E OS=Homo sapiens OX=9606 GN=H2BC21 PE=1 SV=3	7	1	1	0	126	13.9	10.32
High	E9PCA1	CCT-epsilon OS=Homo sapiens OX=9606 GN=CCT5 PE=1 SV=1	5	1	1	0	520	57.1	5.55

Table S5 - Process network analysis (MetaCore) of NORMO EVs protein cargo by GO

Process networks	p-value	FDR
Cytoskeleton_Regulation of cytoskeleton rearrangement	1.034E-15	1.561E-13
Immune response_Phagosome in antigen presentation	3.928E-15	2.965E-13
Protein folding_Folding in normal condition	6.918E-14	3.482E-12
Cell adhesion_Integrin-mediated cell-matrix adhesion	1.332E-10	5.027E-09
Cytoskeleton_Intermediate filaments	5.503E-10	1.662E-08
Cytoskeleton_Actin filaments	7.292E-10	1.835E-08
Translation_Elongation-Termination	6.212E-09	1.340E-07
Proteolysis_Ubiquitin-proteasomal proteolysis	2.699E-08	5.095E-07
Immune response_Antigen presentation	3.553E-08	5.962E-07
Protein folding_Response to unfolded proteins	4.009E-08	6.054E-07
Development_Neurogenesis_Axonal guidance	3.605E-07	4.948E-06
Translation_Translation initiation	6.269E-07	7.708E-06
Reproduction_Progesterone signaling	6.636E-07	7.708E-06
Development_Neuromuscular junction	7.858E-07	8.475E-06
Cell adhesion_Cell junctions	2.161E-06	2.176E-05
Reproduction_Spermatogenesis, motility and copulation	3.077E-06	2.904E-05
Cell cycle_G2-M	4.801E-06	4.264E-05
Protein folding_ER and cytoplasm	5.503E-06	4.616E-05
Cell adhesion_Integrin priming	1.836E-05	1.459E-04
Cytoskeleton_Macropinocytosis and its regulation	6.206E-05	4.686E-04
Signal Transduction_Cholecystokinin signaling	1.118E-04	7.578E-04
Cytoskeleton_Cytoplasmic microtubules	1.149E-04	7.578E-04
Reproduction_FSH-beta signaling pathway	1.154E-04	7.578E-04
Reproduction_Male sex differentiation	1.271E-04	7.999E-04
Proteolysis_ECM remodeling	2.144E-04	1.295E-03
Cell cycle_Mitosis	2.573E-04	1.495E-03
Immune response_Phagocytosis	3.502E-04	1.958E-03
Signal transduction_WNT signaling	4.755E-04	2.564E-03
Inflammation_IL-6 signaling	5.253E-04	2.735E-03
Response to hypoxia and oxidative stress	7.313E-04	3.681E-03
Inflammation_Kallikrein-kinin system	1.173E-03	5.713E-03
Reproduction_Feeding and Neurohormone signaling	1.373E-03	6.480E-03
Protein folding_Protein folding nucleus	1.920E-03	8.785E-03
Cell adhesion_Amyloid proteins	2.446E-03	1.086E-02
Translation_Regulation of initiation	2.927E-03	1.241E-02
Signal transduction_CREM pathway	3.027E-03	1.241E-02
Cell adhesion_Platelet aggregation	3.042E-03	1.241E-02
Immune response_IL-5 signalling	4.005E-03	1.592E-02
Proliferation_Positive regulation cell proliferation	5.702E-03	2.208E-02
Neurophysiological process_Corticoliberin signaling	6.052E-03	2.285E-02
Inflammation_Amphoterin signaling	7.526E-03	2.706E-02

Proteolysis_Connective tissue degradation	7.526E-03	2.706E-02
Cell adhesion_Glycoconjugates	8.636E-03	3.033E-02
Cell adhesion_Cell-matrix interactions	1.157E-02	3.970E-02
Signal transduction_ERBB-family signaling	1.418E-02	4.599E-02
Inflammation_Histamine signaling	1.436E-02	4.599E-02
Inflammation_Neutrophil activation	1.436E-02	4.599E-02
Development_Blood vessel morphogenesis	1.480E-02	4.599E-02
Cell cycle_Meiosis	1.492E-02	4.599E-02
Neurophysiological process_Circadian rhythm	1.558E-02	4.705E-02
